# How Complementary and Alternative Medicine Practitioners Use PubMed

**DOI:** 10.2196/jmir.9.2.e19

**Published:** 2007-06-29

**Authors:** John Willinsky, Mia Quint-Rapoport

**Affiliations:** ^2^University of TorontoTorontoONCanada; ^1^University of British ColumbiaVancouverBCCanada

**Keywords:** PubMed, research dissemination, complementary and alternative medicine, open access, professional development, information retrieval, information management, literacy

## Abstract

**Background:**

PubMed is the largest bibliographic index in the life sciences. It is freely available online and is used by professionals and the public to learn more about medical research. While primarily intended to serve researchers, PubMed provides an array of tools and services that can help a wider readership in the location, comprehension, evaluation, and utilization of medical research.

**Objective:**

This study sought to establish the potential contributions made by a range of PubMed tools and services to the use of the database by complementary and alternative medicine practitioners.

**Methods:**

In this study, 10 chiropractors, 7 registered massage therapists, and a homeopath (N = 18), 11 with prior research training and 7 without, were taken through a 2-hour introductory session with PubMed. The 10 PubMed tools and services considered in this study can be divided into three functions: (1) information retrieval (Boolean Search, Limits, Related Articles, Author Links, MeSH), (2) information access (Publisher Link, LinkOut, Bookshelf ), and (3) information management (History, Send To, Email Alert). Participants were introduced to between six and 10 of these tools and services. The participants were asked to provide feedback on the value of each tool or service in terms of their information needs, which was ranked as positive, positive with emphasis, negative, or indifferent.

**Results:**

The participants in this study expressed an interest in the three types of PubMed tools and services (information retrieval, access, and management), with less well-regarded tools including MeSH Database and Bookshelf. In terms of their comprehension of the research, the tools and services led the participants to reflect on their understanding as well as their critical reading and use of the research. There was universal support among the participants for greater access to complete articles, beyond the approximately 15% that are currently open access. The abstracts provided by PubMed were felt to be necessary in selecting literature to read but entirely inadequate for both evaluating and learning from the research. Thus, the restrictions and fees the participants faced in accessing full-text articles were points of frustration.

**Conclusions:**

The study found strong indications of PubMed’s potential value in the professional development of these complementary and alternative medicine practitioners in terms of engaging with and understanding research. It provides support for the various initiatives intended to increase access, including a recommendation that the National Library of Medicine tap into the published research that is being archived by authors in institutional archives and through other websites.

## Introduction

This study considers the use of PubMed (www.pubmed.gov) by practitioners of complementary and alternative medicine (CAM). In 2001, Americans invested US $50 billion in this form of health care, according to John Weeks, editor of *The Integrator*, a newsletter tracking “the business of alternative medicine,” with 40% of American adults turning at some point to alternative medicine [1]. While some in the medical research community have argued that “there is no alternative medicine” as “there is only scientifically proven, evidence-based medicine supported by solid data, or unproven medicine for which there is no scientific evidence” [2], the US National Center for Complementary and Alternative Medicine (one of the National Institutes of Health) sponsors research in this field and serves as a clearinghouse for clinical trials and research summaries.

Within the scope of its standing as the largest bibliographic index on the life sciences, PubMed (containing Medline, formerly known as Index Medicus, as a subset) indexes medical research articles and includes material useful for physicians and pharmacists, as well as traditional Chinese medicine practitioners [3,4]. A study of nurses, health care assistants, midwives, and health visitors (registered nurses or midwives who promote mental, physical, and social well-being in the community) has shown that, among those who had sufficient access to online sources of health information, the access to journal literature was felt to have led to improved patient care [5].

A prerequisite for the successful pursuit of health information is what Norman and Skinner identify as eHealth literacy, or the skills that enable people “to seek, find, understand, and appraise health information” [6]. This study focuses on how, among a group of CAM practitioners, those abilities are supported by an array of PubMed tools and services. Up to this point, investigators have recognized that users of online health services are not a monolithic group. Studies have been conducted on the reading levels demanded of cancer patients [7], the overwhelming quantity of information available for first-time mothers [8], the limited search skills of college students studying health [9], the growing use of electronic resources by nurses with limited search skills [10,11], and the growing confidence among doctors in their search strategies, although few use this skill on a real-time basis with patients [12].

The variety of approaches that these studies apply testifies to the idea that reading health literature online is situation specific and calls for context, and that investigations into eHealth literacy are fruitful when specific populations are delineated. Information retrieval studies have been done on citation retrieval [13] and literature searching [14] of PubMed, as well as on the use of the medical subject heading (MeSH) and Limits services [15]. Work has also been done in advising users how to best conduct CAM searches [16,17] with at least one study finding that PubMed indexing is not yet adequate for this field of medicine, especially with CAM clinical trials [17].

This study is intended to complement the more common focus among information science researchers on users’ information retrieval strategies with indexing services such as PubMed [18,19]. It addresses the value of PubMed’s specific tools and services for CAM practitioners, as these tools are introduced to them in the context of their own interests in the life sciences and their interest in learning about the use of PubMed. In focusing on the value and role of the tools and services, we are drawing on literacy and learning research in the subject domains, or “domain knowledge” as Alexander and others refer to it [20]. Alexander has established the degree to which a combination of personal interest and background knowledge contribute to the degree of learning in domain areas such as biology and physics, and she holds, in particular, that “one’s knowledge base is a scaffold that supports the construction of all future learning” [21].

In this sense, PubMed’s provision of tools and services (such as Related Articles and Bookshelf) can be seen to help users easily and immediately augment, in a limited way, shortcomings in their knowledge base. The tools and services compensate for shortcomings not only in searching for articles but also when comprehending, evaluating, and utilizing abstracts and full-text articles. The tools and services (such as Full-Text Access, Email Alert, Send To) also appear to be poised to support the user’s interest in and engagement with the indexed material (which is the other critical factor Alexander has identified for domain learning). By introducing CAM practitioners to the tools and services, one by one, and gaining from them a sense of the perceived value and contribution of each, this study seeks to determine the potential contribution of these tools and services to the knowledge-base scaffolding and the personal interest of health care workers who lie outside of PubMed’s originally intended audience of researchers and physicians. This work has obvious implications for the design of the tools, in PubMed’s effort to serve as broad an audience of health care practitioners as possible, and as a way, in turn, of extending the impact and benefits of life science research.

## Methods

### Sample

The participants in this study were drawn from among those practising CAM. They were recruited from three sources: (1) participants 1-5 were drawn from among students taking an online professional development course at a community college on research literacy for the health professions, (2) participants 6-9 were recruited from the Massage Therapy Association of British Columbia, and (3) participants 10-18 were drawn from the Canadian Memorial Chiropractic College. The eight women and 10 men who agreed to participate in the study ranged in age from their 20s to their 50s. Of the 18 participants, 10 were pursuing or possessed a chiropractic degree, seven were registered massage therapists, and one practised homeopathy. A total of 13 participants had a university degree, with one holding a master’s degree and another, a PhD. In terms of background with online research, participants 1-5 were taking a course on research literacy, while participants 13-18 had been introduced to the topic in a session with a librarian, with some one-on-one follow-up.

### Design

Participants were offered an opportunity to learn how to use PubMed in one-on-one sessions with the researcher (MQ-R) in exchange for helping the researchers understand the value of PubMed’s various features to users such as themselves. The 10 PubMed tools and services considered in this study (Table 1) can be divided into three functions: information retrieval (Boolean Search, Limits, Related Articles, Author Links, MeSH), information access (Publisher Link, LinkOut, Bookshelf), and information management (History, Send To, Email Alert). The sessions with the participants took place between April 2004 and December 2006 and averaged 2 hours in length. Sessions held with participants 1-9 were conducted over the phone, with both participant and researcher accessing PubMed through the Internet, while sessions with participants 10-18 were conducted face-to-face with a single computer connected to the Internet, which the participant operated. Participants were introduced to between six and 10 PubMed tools. The participants in the phone sessions covered 7.1 items on average compared to 7.7 items covered by participants in the face-to-face sessions.

Prior to their PubMed training session, the researcher asked participants by email to identify a health science topic of interest to them. The researcher then found a suitable article based on the interest of the 14 participants who responded and sent it to them in advance of the PubMed session (Multimedia Appendix 1). The participants were asked to read the paper prior to the session. The starting point for each session, after introductions and preliminary questions about the participant’s background, was an initial search of PubMed on a topic of interest. Participants were then guided through a range of six to 10 PubMed tools and services depending on the pace of the 2-hour session and where the interests of the participants led (Multimedia Appendix 2). For each tool and service, they were introduced to how the feature worked, using concepts and papers of interest to them, and were asked to provide their thoughts on its potential value for their use of PubMed.

**Table 1:** PubMed tools and services introduced to participants

Tool/Service	Function
Boolean Search	Search terms can be entered using Boolean operators AND, OR, NOT.
Search Limits	Allows the user to limit the search by language; type of journal; gender; human or animal subjects; age; type of article; date; inclusion into PubMed.
Related Articles	This appears as a button/link with each citation and brings up a list of matching articles based on the original article.
Full-Text Access	This is provided through the publisher’s link on the abstract page and on the LinkOut page, with publisher, aggregator, PubMed Central, and/or subscribing libraries providing open access for roughly 15% of articles.
Send To	Send To allows users to email citations, convert citations to a text file, or save citations for editing and further action.
Search History	This tracks terms, time, and number of results in searches, while allowing Boolean searches combining different searches from the Search History.
Bookshelf	This provides links to relevant passages in a set of full-text life science books that range from basic science to clinical practices.
MeSH Vocab	MeSH is a controlled vocabulary database for ascertaining the most commonly used terms in PubMed.
Email Alert	The National Center for Biological Information (NCBI) of the National Library of Medicine (NLM) offers email notification of new work in specified topics.
Author Links	Authors’ names have links leading to a listing of all of their work in PubMed.

### Analysis

The sessions were transcribed (Multimedia Appendix 3), and the two researchers created a summary table of the results by rating the responses for each tool as (+) positive (“that’s valuable”), (++) positive with emphasis (“I’d really use that”), or (−) negative or indifferent (“that makes sense”). Because of the session’s 2-hour time constraint and the semistructured handling of the sessions, not every tool was covered with every participant. As a follow-up to the PubMed session, the researcher emailed the participants 2 weeks later, asking them to describe their current use, if any, of PubMed and other Internet resources.

## Results

What follows is a detailed qualitative analysis of the participants’ responses to each of the 10 PubMed tools and services covered in this study, with the aim of better understanding how such aspects of PubMed might be seen as contributing to its use by the participants. Differences in the responses among the participants (on the basis of training or university degree) was not the focus of the study, although, as might be expected, those with prior training and with university degrees tended to value PubMed tools and services more highly. Participants with prior training in the use of online research literature thought that, on average, 6.0 out of the 7.5 tools and services that were introduced to them would be valuable in their use of PubMed, while those without such training responded positively to 4.6 of the 7.1 items introduced to them (Table 2). Similarly, those with a university degree responded positively to 5.7 out of 7.4 items, compared to those without a university degree, who gave a positive evaluation to 4.8 out of 7.4 items.

**Table 2:** Value of PubMed tools and services for participants (N = 18)

Participants			PubMed Tools and Services^‡^										Further Use of PubMed
Code^†^	University Degree	Prior Online Research Training	Boolean Search	Search Limits	Related Articles	Full-Text Access	Send To	Search History	Bookshelf	MeSH Vocab	Email Alert	Author Links	
MF1	yes	yes	+^*^	+^*^	+	++	+	++^*^	−	−^*^	+^*^		yes
CM2	yes	yes	++	+	++	+	+^*^		−				yes
HF3	no	yes	++^*^	+^*^	++^*^	+	+	++	−			+	no
MF4	yes	yes	+^*^	+^*^	+^*^	+^*^	++		++				
MF5	yes	yes	+^*^	+	+	++			++	+			
MM6	no	no	+	++	++	++^*^	−		+	−		−	
MF7	no	no	++	−	++	+	+		++				yes
MF8	no	no	−	−	+	++	−		−	+		−	yes
MM9	yes	no	+	−	−	+	−			++		−	no
CF10	no	no	++	++	−	+	−	+		−			yes
CM11	yes	no	++	−	−	++	−	++		+	++	+	yes
CF12	yes	no	+	+	−	++	++			++			yes
CM13	yes	yes	+	−	+	+	−	+	−		+	+	no
CM14	yes	yes	+	−	+^*^	+	+^*^	++^*^	−		+		
CM15	yes	yes	−	++	++	++	++	+^*^	+	−			yes
CM16	yes	yes	+^*^	+	++	+^*^	++	+	−				
CM17	yes	yes	+^*^	+^*^	+	++^*^	++	+	−	−^*^	+		
CM18	yes	yes	−	++	−^*^	+^*^	+	−	+				

^*^Prior familiarity with tool or service.

^†^First letter of code: M=registered massage therapist; C=chiropractor; H=homeopath. Second letter of code: M=male; F=female.

^‡^ +: positive evaluation of tool or service; ++: additional positive emphasis; −: negative or indifferent response to tool or service Blank cells indicate that tool or service was not covered in the participant’s session or participant did not respond to email follow-up.

### Boolean Search

The basic starting point with PubMed, as with any online index or database, is the search, which typically allows the combining of search terms with AND, OR, or NOT for more accurate searching. These Boolean operators were familiar to a number of participants, but were a little vague for others: “I might have heard of it” (CF12). Learning about them made a considerable impression on a number of participants:

CM2 I will use the Boolean search methods even when using the different features of the database. This is incredibly important to know, use, and understand. It’s like the building blocks of searches. I’ve got to practise that one afterwards.

At least one participant recognized that the search results were profoundly influenced by the terms of the search:

CM11 The difficulties is [*sic*] the prejudice you take to your search question. Maybe you missed something because you describe[d] it badly.

### Search Limits

**Figure 1:**
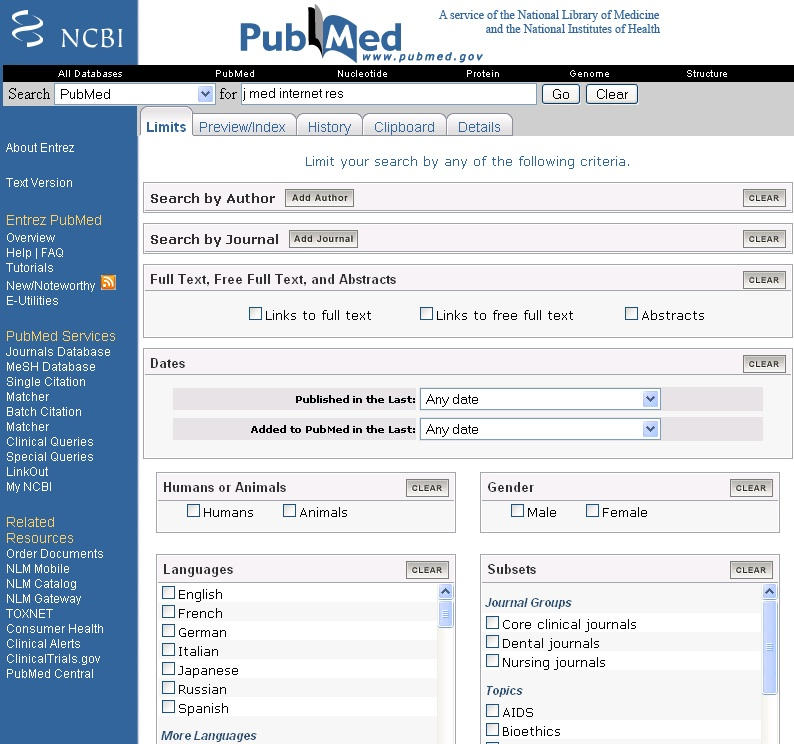
Limits tab on PubMed

A further search strategy that the participants found useful was the ability to apply Limits to the searches, including dates, author, links to free full-text articles, language, gender, human subjects, type of article, and so on (Figure 1): “I have used Limits with dates mainly...and if I am searching for a particular author” (MF1). Limits seemed useful for participants for finding specific information, such as when they needed to answer a particular question: “I think I would use this one [Limits] to look for something very specific, but if I wanted to browse around I would use Related Articles” (MF5), and “I think I would use the Limits if I have a problem patient who comes at me with a Google paper, like if she was a 45-year-old female who found something and said, ‘Why don’t I try this?’” (CF10). The participants found Limits useful because it allowed them to be selective about the information they would find, such as limiting their searches specifically to include only core clinical journals. This is because “they [core clinical journals] are well recognized in the medical community” (MF7), and, as another participant explains, “Because sometimes I don’t want to know...what a really alternative journal says, I want to read the larger stuff” (CF12). 

### Related Articles

**Figure 2:**
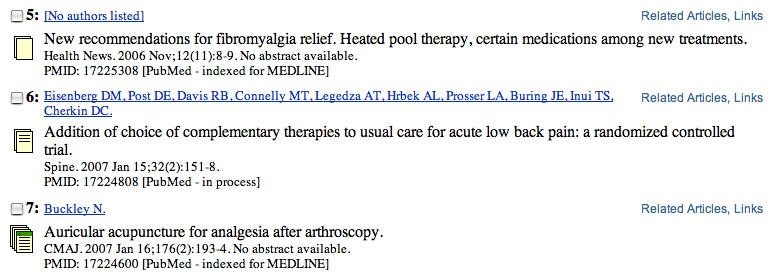
Partial results of a PubMed search on “complementary medicine,” showing icons (from top to bottom) for “no abstract,” “abstract only,” and “open access to full-text article” (although no abstract is available) and hyperlinks to “Related Articles” and “Links” (to the full text on publisher and/or library websites)

The ability to identify one very promising article and then find all of the related articles by clicking on a hyperlink (Figure 2) was highly valued by five participants: “I really like Related Articles; I saw it in the other database, and it is useful because it brings me to things that I would probably not have found otherwise, outside my own searching” (CM2). However, participants also intuited how Related Articles could be used in relation to Boolean searches to achieve a very efficient focus on the desired research: “These look really great.… I could spend hours just seeing what comes up from this feature after starting from one search” (HF3), and “This list [based on Related Articles] makes more sense to me than the original list that came up” (MF4). It did seem to a number of the participants that “Related Articles worked perfectly” (MF7). Yet, Related Articles did not work for everyone, as one chiropractor commented, “What [PubMed] thinks is similar is not what I think is similar” (CM18).

### Full-Text Access

PubMed provides access to the complete articles that it indexes through a publisher’s link on the article’s abstract page (see Figure 2), as well as a link to PubMed Central if the journal’s contents have been placed in this open-access repository. In addition, LinkOut provides links to sites where the article can be accessed, including sites of the publisher, journal aggregators such as EBSCO and ProQuest, and university libraries that hold print and/or electronic editions of the article (which are accessible to members of those libraries). Many of the publishers and the aggregators provide nonsubscribers the ability to purchase access. For example, Elsevier’s Science Direct changes US $30 to purchase an article in any of its 2200 journals. In addition, PubMed clearly identifies that a small proportion (roughly 15%) of the literature has been made “open access” by its publisher, either immediately upon publication or after a certain period (“moving wall” model), typically from 6 to 24 months following publication.

While the article’s abstract was typically included in PubMed’s indexing of an article, it was judged insufficient by participants as a way of both understanding and evaluating the research, whether they had had very little experience with the research or were well oriented to their field’s literature. As one first-time PubMed user stated as she looked at an abstract, “[I] definitely need to read the article. It [abstract] does not tell us enough” (MF8). This response is consistent with previous studies on the shortcomings of abstracts [22-24].

For most participants, the abstract was necessary as a starting point but was insufficient as source of information on its own. It served as a screen, as it would for a researcher:

CM2 So an abstract is really useful. If I felt the abstract was interesting, I would definitely need the full text to actually find the article useful to my work. An abstract only provides enough information to pass the first screening test.

This chiropractor initially felt, on first looking through the list of PubMed search results, that “This information is not what I am looking for; it is too technical, too specialized.” However, by the time he had had the chance to download and look at an open-access article, he was expressing regrets: “This is incredible; it is so easy; I wish I could just finish reading the article right now” (CM2). Frustration over not being able to view full-text articles was a common theme: “There was an article that I was interested in looking at, and it said that it would not be available for about a year or 6 months” (CM14). “The only problem,” as a registered massage therapist put it, “is that the ones that are free are not the ones I was wanting” (MF8).

For some, visiting a research library was not an option, and the cost of purchasing a single article posed a serious obstacle: “It deters me, big time, having to pay for articles” (MF8), and “Oh, you can order papers; I don’t have money to order papers” (CM14). For others, using a credit card online was an issue: “I don’t think that that’s a safe measure, with my credit card online” (MF5). Yet the interest in seeing an article was sufficient that a number of participants were prepared to give serious consideration to paying to download it: “It is incredibly important to have access to the full text of the article. I would even pay to access a full-text article” (CM2); “I think I’ll look for mostly free articles so I could actually read them. If I was desperate to find information and couldn’t find anything, then I might actually buy it” (HF3), and “depending on how valuable the article is I might be willing to do that” (MF7).

For one chiropractor, who administered a professional chiropractic organization, having access to the full-text article was a must. He felt that the full text offered a more accurate picture of the research and provided the language he needed to use for his own work, which was to lobby the Canadian government to further integrate chiropractic into standard public health policies. As he explained,

CM11 No one who has ever done research has ever done an abstract that exactly covers the detail of what’s done inside. So when you actually read the research paper you see more…. The abstract…never provides that robust meaty quote that I need.

He went on to give an example from the *New England Journal of Medicine* in which the results reported in the body of the article placed the chiropractor’s treatment of childhood asthma in a far better light than the abstract did.

Another chiropractor conveyed the frustration of coming across a very good source of research, only to find access to the resource restricted. In this case, it was a systematic review conducted by the Cochrane Collaboration, which are not freely available:

CM14 It says [reading from the abstract] “evidence which is based on the Cochrane review of the subject.” This review is like topnotch.... It’s called a Cochrane collaboration, and we don’t have free access to that.... It would be phenomenal if we had access to that.... It’s all based on a strict protocol, like how many people were in the study.... If you want to know the vast information about a subject, you should go to Cochrane reviews.

Even in the case of a chiropractor who felt that “often times I get most of the information I need from the abstract” (CM17) and thus did not need the full text, there was a recanting after a few moments:

CM17 Well, that’s not true. I do [need access to the full text]. Often times you are stuck because they tell you “in the article we describe” such and such. And then you do everything to get it. And we have different ways to get articles.

A registered massage therapist noted how much she appreciated it when the researcher sent, prior to her session, the full text of an article of interest:

MF7 I especially valued that it was not just a summary.... It is very relevant to my work as a therapist, and as an instructor. [I] also found the references very helpful.

And finally, a registered massage therapist who had recently obtained her MSc from a Canadian university, and thus had lost her ready access to the scholarly literature upon graduation, had learned about the issue of access in terms of how libraries were signing a new type of agreement with the journal publishers, which carefully controlled access:

MF1 The contracts that the academic institutions sign with the publishers, limiting access to students and faculty only, mean less access for the public. When hard copies were available, anyone could read, digest, copy relative sections, etc. Now, depending on the institutional policy, it may be difficult even to get access to view a full text. Needless to say, the cost of individual articles is exorbitant.

The library would allow the public access to its online journals at a small number of “public” terminals (for those designated as “walk-ins” in the contract), but as this participant noted,

MF1 [The university libraries] control [public access] very, very, very tightly. I went in and asked if I could use the computer.... I had 6 articles I wanted to see, and they let me do that.

She concluded that this represented “the publisher’s stranglehold on the universities” (MF1). Another participant had caught wind of a hopeful agreement that would greatly increase access to medical research, not only for him, but the public at large:

MM6 I was talking to a librarian here at the public library and she said in [British Columbia, Canada] they were...trying to link the public library with the university library so that anybody that has a library card [could view the journals].

It was PubMed’s clear identification of, as well as links to, the open-access articles that was the one aspect which participants consistently valued. The participants felt strongly about the importance of being able to read the full text, and while a few had access to research libraries (McGill and University of British Columbia cited), others were willing to consider paying for that access. But in all cases, the value of open access to this literature was judged as a critical aspect in becoming better informed about what the research had to offer their professional practice.

### Send-To Tools

Users are able to select, organize, and save the citations they find by sending the selected citations to a file, text, printer, clipboard, email, or RSS feed. Five of the participants were very impressed by how this tool allowed one to build a personal set of research resources:

CM2 I’ve done this before [email search results to self] when I have found research on the Web. I think it’s great; it is very easy, and I like to keep things in my inbox. This is a great tool that I would use.

Another chiropractor commented on how it enabled him to integrate PubMed into his work:

CM11 Over the course of the day, I can sort it and then I have my list in order. Now I can take that display list and send it to text.

### Search History

PubMed keeps a record of each search that a user conducts, under History, allowing users to not only review their previous searches but to combine them using Boolean operators. “Wow. That’s amazing. If I ever want to go back to what I did, this is how. It’s like you don’t have to write anything down because it is all recorded automatically. This is really, really great” (HF3). While this tool only made it into the sessions of 10 participants, only one of those participants proved relatively indifferent to its powers: “That makes sense” (CM18).

### Bookshelf

PubMed has integrated a number of full-text medical and life science books that can be searched separately or in conjunction with an article abstract to gain background information about key terms in the abstract (which are highlighted with links to the relevant passages in the books). Only one participant saw how access to the books might be used for providing background: “I really like it; if I am writing a thesis or a paper, I would see this as very useful” (MF5). More frequently, the participants in the study made a number of observations about the currency and focus of the research articles as the reasons they preferred them over books: “Well that’s interesting [referring to Bookshelf], but I like articles because they are more current” (MF1), and “I like journal articles because they are more specific” (HF3).

A chiropractor reinforced this idea, to a degree that suggested how the background role of PubMed’s Bookshelf program was being missed by some:

CM14 That’s interesting [referring to Bookshelf]. But...once you get into reading research articles, they are much better than reading books. Books are always behind a few years.... There is a lot of bias behind the author of the book—reading an article, you get the actual truth.

Such responses suggest that the participants were picking up ideas about the importance of currency and the leading edge role of journals in the life sciences, ideas that need to be refined, certainly, but that are not far removed from common thinking within the research culture.

### MeSH Vocabulary Database

The MeSH Database enables readers to look up the controlled vocabulary used to index the research literature. Among the 10 participants introduced to MeSH, there were challenges initially in comprehending it, to a degree not experienced with the other tools and services: “I don’t understand; what is this [MeSH Database]? Is this everything with the keywords?” (CF12), and “Well, I have taken the tutorial [on the MeSH Database], and I’ve tried to use it, and I know it provides you with headings” (MF1). However, with further explanation and a chance to work with it, at least one participant found it “totally awesome and very useful” (MF8), and another appreciated that “it helps to distinguish things, and so you can go in one direction or another depending on what you want” (MM9). However, that same participant pointed out that what he felt was an element of bias:

MM9 The word “subluxation”...means something quite different to a medical doctor than it does to a chiropractor when it comes to small tiny restriction in motion or small tiny misalignment. But the medical profession still doesn’t believe such things can occur, so I see here that there is no such thing here as subluxation in the sense that the chiropractor means it—very interesting.

PubMed’s sharing of its vocabulary used for indexing also spoke to the value of this service as a source of engagement and reflection.

### Email Alert and Notification Services

The National Center for Biotechnology Information (NCBI) of the National Library of Medicine (NLM) is responsible for PubMed and offers users a personalized service that includes email alerts when materials of interest to them are indexed. While this tool came up in only five of the sessions, there was recognition of its value by all the participants: “I have an automatic referral for [from] NCBI every so often about updates of new research in this area” (MF1). A chiropractor had signed up with a similar service, PubCrawler, developed by a genetics research lab at Trinity College, Dublin, for alerting readers to new articles in areas of interest: “I’ve signed up to PubCrawler, which alerts me to new research in my area, and so [I] quite often scan these [almost daily]” (CM11).

### Author Links

The ability to find other works by the same author, simply by clicking on the author’s name, was not generally valued by the participants with the exception of the homeopath and one of the chiropractors: “So I have heard of him before. I would think it would be interesting; it would be interesting to know what his perspective was on other things” (CF12). With the six participants who encountered this feature divided between interest in and indifference toward it, it may seem that this way of focusing on the work of an individual researcher is not as valued as the other means of tracking work.

### Comprehending Research

In the course of reflecting on PubMed tools and services, some of the participants reflected on their understanding, as well as their critical reading and use, of the research they were searching. For example, a number of participants commented on the value of reading the abstract as a necessary first step in reading research: “I wouldn’t read a paper without an abstract” (HF3). By the same token, the abstract was not enough: “Definitely need to read the article. [Abstract] does not tell us enough here” (MF8). A chiropractor noted that the figures and illustrations available in the full text were needed to make sense of the orthopedic tests used in the study:

CM15 [The abstract is] a good summary, but...if the article had pictures and stuff, because maybe these are new tests that have come out that the school hasn’t taught us yet, and they might be better than the ones we’ve learned...[then access] would be important.

Another chiropractor spoke of the importance of seeing the research methodology described in detail:

CM14 The abstracts usually don’t show enough about the methods of an article.... It depends on the method, if the paper is actually good.

Similarly, a third chiropractor, who was satisfied with the abstract alone when it came to “advice or recommendations,” felt that when the “treatment” of a client was at issue, then more than the abstract was needed:

CM18 I can’t break down the methods they used [with the abstract alone], and if there’s any flaws in it...I won’t get it from the abstract. So I would like to see the whole thing.

The participants in this study were aware of their limits in understanding the research, which concerned specific domains of knowledge, as this chiropractor made clear: “I am trying to find one that is not as ‘biochemistry’ to read; those ones are terrible to read.” Yet, within a given search, the same participant was able to find research studies which, in dealing with “exercise for treating fibromyalgia,” were extremely helpful and comprehensible to the point where he “would want to prescribe” those exercises to his clients (CM16). More than one registered massage therapist was prepared to challenge the limits on her own understanding: “There’s a lot of qualitative research out there on complementary therapies, but not a lot of quantitative [work], so I am looking for quantitative-oriented stuff” (MF5). However, she realized that she was not clear on what “the *p* factor” (*sic*) was and stated that “I’m not really up with statistics.... I can understand some but not all” (MF5). A second registered massage therapist was willing to push her learning beyond the concerns of clinical practice: “Well, it’s not useful for me, for what I do, but it does tell me some information that I didn’t know before: the cell type that they are talking about, the engagement of it” (MF7).

If participants felt somewhat overwhelmed by the research at times—“It’s a little above my head” (CM13)—they also knew how to direct their reading in relation to their strengths. As a registered massage therapist put it: “This is a little too theoretical and general...[while I’m] looking for protocols for treating low back pain.... I’d be more on ‘protocol,’ ‘orthopedic,’ ‘massage therapy’” (MM9). Or, as a chiropractor explained: “It’s a bit too heavy; it is going to go beyond what I need clinically” (CF10). What was perhaps most notable about the participants’ responses to the research was their perception of the value of this public resource to their professional practice: “It’s pretty encouraging to find this [material in PubMed]; it’s not intimidating” (MF7), and “I use the database and feel quite comfortable with it” (CF10).

### A Growing Research Culture

This research on CAM practitioners’ use of a life sciences research index reflects a growing influence of a research culture among the health professions and the public. Two participants made reference to their clients bringing in online health information:

MF5 My problem is telling [my clients] that what they are finding in Google is not that sound scientific information because any practitioner out there, anybody can put something out onto Google or their office website, but it’s not necessarily valid. I always have clients who are asking me about information that has come out on certain treatments, so that is primarily what I would tend to use this [PubMed] for.

That same increased interest in recent research had been expressed within the CAM community as well, as the administrator of a chiropractic organization stated:

CM11 Our membership expects us to know things instantly if they are in the public domain. They say, "Why don’t you know? Everyone else knows”.

The increasing prominence of research in the health services sector was also reflected in the training received by graduates of the Canadian Memorial Chiropractic College:

CM17 This school is very evidence-based. So a lot of the assignments are pretty much looking up research: sensitivity, specificity, tests, prognosis.... If you are efficient with any of these search engines, you can find the information quite quickly. I have used it in university as well, but not to a great extent. When I got here I had to use it much more. It depends on the clinician that you get; some are really, really, really, evidence-based, meaning they follow everything the research says.

Another participant noted:

CM16 They [at the Canadian Memorial Chiropractic College] talk so much about evidence-based care in medicine and chiropractic, and you have to have research articles in order to attempt to that. You need a way to do it and PubMed is the only way I know.

He went on to add that “it is free.” This was further confirmed by a classmate: “They [the school] thrust that down our throats: ‘Make sure you are up to date on that’” (CM15).

In turn, CAM practitioners are participating in the debates on the growing prominence of certain forms of medical research but in the following example also demonstrate limited understanding of the methodology:

CM11 I wrote an article about [how]...it is more or less impossible to do a [randomized clinical trial] of chiropractic. You can’t blind a practitioner; it’s more or less impossible to blind the patient, so any effect of randomization is nonsense.

Overall, there is a growing global awareness of the important role played by research in the life sciences, and these CAM practitioners are learning that this body of knowledge has much to offer, with PubMed providing one means of obtaining access to it: “There’s a lot going on in Europe and Asia that I know nothing about unless they publish in *Lymphology* or unless they present at the ISL [International Society of Lymphology]” (MF7).

### Continuing Use of PubMed

In response to follow-up emails that were sent out some weeks after the session with the researcher, seven participants affirmed that they were using or were planning to use PubMed:

CM2 Yes, I have used PubMed since our last conversation. I use it on a weekly basis. I use it to find information for patients and for myself. I am in the process of developing an acupuncture-related website and writing a book on health-related topics.

Issues of access to research articles persisted in the use of PubMed for this chiropractor:

CM2 I have not purchased any articles, although I have been tempted. I tend to look at the full-text articles, and I have used the links LinkOut and Bookshelf with success.

As well, he had begun to see how he could best use PubMed in his practice:

CM2 I think I’ll use it to get research, to get on-the-fly research to answer questions. But I wouldn’t send my patients to use the database or conduct searches on this database after a consult with them because I think it would be too complicated. I would not refer them to PubMed to find their own research.

There were also indications from the participants of PubMed raising the quality of knowledge that informs their practice: “I’m using [PubMed] now to look something up instead of Google” (CF12).

Other participants spoke in the follow-up emails of their plans for continuing use of PubMed:

CM15 Now that I know more about it, I think I’ll use it more.... Now that we have had this session, I’ll be able to understand it a little bit better, especially this "Related Articles".

This same participant had explained in his session with the researcher how the Limits tool would serve his practice: “[This] would be great if I had a patient come in with a problem, and I had to really specify my search according to his needs” (CM15).

 Another chiropractic student spoke of PubMed use in terms of its public access as a source of professional development:

CM17 I am not going to be in academia much longer. It would be great to know as much as I could before I leave. I plan on using this when I leave as well.

Of course, not everyone who participated in this study went on to use PubMed afterward:

HF3 I often learn about research studies from [online] medical news sources [eg, *Medical Post*] and access them through links provided in the article versus searching PubMed. There is so much information out there, and I find it hard to find time to access it all!.

A similar theme was sounded by a massage therapist:

MM9 I have no urgent reason to [use PubMed], and my time is too occupied to browse.... Currently, I do not [need PubMed].

And a chiropractor wrote to say that while he is sometimes “directed to PubMed as a resource” at his school, the problem is that he “could never really get the entire PDF files” that he wanted (CM13).

## Discussion

This study confirmed our hypothesis that CAM practitioners would find that certain PubMed tools and services, on being introduced to them, had the potential to contribute to their engagement with and understanding of the research that interested them. This study did not seek to measure the actual contribution of these tools and services to the users’ learning. It was designed to establish whether these users perceived the tools and services as having value, as a first step to subsequent studies that will assess the differences that individual tools might make to the participants’ learning.

Among the PubMed tools and services to which the participants were introduced, only the service of full-text access proved a positive asset for engagement, comprehension, evaluation, and utilization. As well, the Boolean Search and Related Articles were also strong contributors to the participants’ work with PubMed, and all tools and services impressed more than a few participants as to their value and contribution.

In terms of the specific design of PubMed, it is clear that the NLM is continuing to improve its design and functionality [25]. For example, the participants found MeSH to be a particularly difficult tool to grasp; however, the NLM has since provided three animated tutorials (eg, ”Searching with MeSH”) with voice-over, which make its operation much clearer, with at least one recent study attesting to MeSH as the preferred search strategy [26]. Still, one of this paper’s reviewers wisely advised that training in MeSH would greatly improve user searches, and the ways in which a user’s health vocabulary contributes to recognition and comprehension has been the subject of a recent study [27].

There have been design improvements, as well, with Related Articles, Limits, and other tools. With Bookshelf, the only feature that left over half of the participants who tried it seeing little value in it, PubMed has withdrawn the book links from an article’s abstract, while still offering users the ability to search its book holdings. Yet, these medical and basic science books could well serve as background resources when a reader is stymied by concepts in biochemistry and statistics, to name two areas that this study’s participants identified as weak for them [28]. There is reason, then, for the NLM to continue exploring ways of integrating the books into the literature searches in a way that encourages professionals in the health services to seek background clarification and context, as a way of improving their engagement with the research.

The study also has bearing on what has developed, with the rise of the Internet, into the “access issue.” This goes back to at least 1999, when Harold Varmus, then director of the National Institutes of Health, proposed that all medical research journals make their work freely available in PubMed Central 6 months after publication, an initiative that met with the successful resistance of commercial and society publishers [29]. More recent measures include the National Institutes of Health Public Access Policy, which asks funded researchers to voluntarily deposit copies of their published work in PubMed Central, but which has managed to achieve a less than 4% compliance rate to date [30]. As well, the proposed Federal Research Public Access Act of 2006 is currently before the Senate, which would mandate open access within 6 months of publication for a large proportion of US government–funded research [31]. The results of this study would seem to support efforts to increase access to research by making it clear how greater access could contribute to the practice of CAM, as well as to more traditional forms of clinical practice.

The participants’ consistent interest in having access to the full text of the articles indexed in PubMed leads to a strong and immediate recommendation to improve that access: The NLM should make every effort to capture an otherwise missing and substantial source of open access to research and scholarship, namely, the published health sciences research that has been posted by authors in institutional repositories and on websites. While PubMed has an excellent system for identifying and connecting to open-access articles made available by publishers, it needs to develop similarly effective systems for tapping the published work that authors have posted, with the publisher’s permission, in archives and on websites. With numerous archiving mandates, both in place and pending, for this form of open access to research that has been funded by governments and foundations, PubMed needs to ensure that it is able to take advantage of this movement to greater openness. For example, PubMed could include a means of searching the more than 800 repositories worldwide.

For those who study the use of health information, we see a need to push beyond the obvious limitations of this study. There are sufficient grounds for further studies carefully designed to assess the contribution that particular aspects of these tools provide for different types of users, with a focus on how the design of the tools and services augments the users’ comprehension, evaluation, and utilization of the materials they encounter.

The prospect of the ongoing development of resources such as PubMed, along with an increasing degree of public and professional expectation of access to research and scholarship, holds much promise for the continuing educational quality of the Internet and society at large. While only a fraction of CAM practitioners and a proportion of their clients will pursue these forms of knowledge, there is a larger point to this. Through the combined efforts and commitment of the NLM, a good number of life science journals, and the researchers themselves (who post open-access copies of their work), the quality of knowledge that is publicly and universally available is increasing, adding to people’s understanding as well as to the health care practices that affect their lives. This study is one small demonstration of how the benefits of this greater access extend to a larger community than has been commonly considered when it comes to public resources such as PubMed.

## Multimedia Appendix 1

Articles sent to participants prior to PubMed sessions (pdf)
					

## Multimedia Appendix 2

Searches conducted by participants in PubMed sessions
										

## Multimedia Appendix 3

Complete interview transcript of PubMed sessions
												

## Abbreviations

CAM: complementary and alternative medicine
MeSH: medical subject heading
NLM: National Library of Medicine
