# Supplementary material for: How Complementary and Alternative Medicine Practitioners Use PubMed
Source: J Med Internet Res. 2007 Jun 29;9(2):e19. doi: 10.2196/jmir.9.2.e19 (PMC1913941; doi:10.2196/jmir.9.2.e19)
Supplement: Supplementary file 1 [file jmir_v9i2e19_app1.pdf]

Appendix 1: Articles sent to participants prior to PubMed sessions

Code	Pubmed Citation
MM6	Jacob T, Baras M, Zeev A, Epstein L. A longitudinal, community-based study of low back pain outcomes. Spine. 2004 Aug 15;29(16):1810-7.
MF7	Cheville A, McGarvey CL, Petrek JA, Russo SA, Taylor ME, Thiadens SRJ. Lymphedema management. Seminars in Radiation Oncology. 2003: July13(3): 290-301.
MF8	Bennett, RM, Schein J, Kosinski M, Hewitt DJ, Jordan DM, Rosenthal N. Impact of fibromyalgia pain on health- related quality of life before and after treatment with Tramadol/Acetaminophen. Arthritis and Rehumatism, 2004 Aug 15; 53(4): 519-527.
MM9	Bennett, RM, Schein J, Kosinski M, Hewitt DJ, Jordan DM, Rosenthal N. Impact of fibromyalgia pain on health- related quality of life before and after treatment with Tramadol/Acetaminophen. Arthritis and Rehumatism, 2004 Aug 15; 53(4): 519-527.
CF10	Bennett, RM, Schein J, Kosinski M, Hewitt DJ, Jordan DM, Rosenthal N. Impact of fibromyalgia pain on health- related quality of life before and after treatment with Tramadol/Acetaminophen. Arthritis and Rehumatism, 2004 Aug 15; 53(4): 519-527.
CM11	Sigrell, H. Expectations of chiropractic treatment: what are the expectations of new patients consulting a chiropractor, and do chiropractors and patients have similar expectations? J Manipulative Physiol Ther. 2002 Jun;25(5):300-5.
CM13	1.Willms JD. Early childhood obesity: a call for early surveillance and preventive measures. CMAJ. 2004 Aug 3;171(3):243-4. No abstract available.<br>2.Canning PM, Courage ML, Frizzell LM. Prevalence of overweight and obesity in a provincial population of Canadian preschool children. CMAJ. 2004 Aug 3;171(3):240-2.<br>3.Chauhan TS. Factors contributing to obesity in adolescents. CMAJ. 2004 Feb 17;170(4):457.
CM14	1.Loh YC, Lam WL, Stanley JK, Soames RW. A new clinical test for radial tunnel syndrome--the Rule-of-Nine test: a cadaveric study. J Orthop Surg (Hong Kong). 2004 Jun:12 (1):83-6.<br>2.Genc H, Saracoglu M, Duyur B, Erdem HR. The role of tendinitis in fibromyalgia syndrome. Yonsei Med J. 2003 Aug 30;44(4):619-22.<br>3.Mellor S. Treatment of tennis elbow: the evidence. BMJ. 2003 Aug 9; 327(7410):330.
CM15	Malanga GA, Andrus S, Nadler SF, McLean J. Physical examination of the knee: a review of the original test description andscientific validity of common orthopedic tests. Arch Phys Med Rehabil. 2003 Apr;84(4):592-603.
CM16	Bennett, RM, Schein J, Kosinski M, Hewitt DJ, Jordan DM, Rosenthal N. Impact of fibromyalgia pain on health- related quality of life before and after treatment with Tramadol/Acetaminophen. Arthritis and Rehumatism, 2004 Aug 15; 53(4): 519-527.
CM17	Sigrell, H. Expectations of chiropractic treatment: what are the expectations of new patients consulting a chiropractor, and do chiropractors and patients have similar expectations? J Manipulative Physiol Ther. 2002 Jun;25(5):300-5.
CM18	Szeto YT, Tomlinson B, Benzie IF. Total antioxidant and ascorbic acid content of fresh fruits and vegetables: implications for dietary planning and food preservation. Br. J. Nutr. 2002 Jan;87(1):55-9.

Note: Participants who did not request [?] an article in advance: MF1, CM2, HF3, MF4, MF5, CF12
