# Supplementary material for: How Complementary and Alternative Medicine Practitioners Use PubMed
Source: J Med Internet Res. 2007 Jun 29;9(2):e19. doi: 10.2196/jmir.9.2.e19 (PMC1913941; doi:10.2196/jmir.9.2.e19)
Supplement: Supplementary file 2 [file jmir_v9i2e19_app2.pdf]

JMIR Multimedia Appendix 2 (www.jmir.org - Vol 9 Iss 2 e19)

Searches conducted by participants in PubMed sessions.

Code	Basic Search	Boolean Search	MeSH Search
MF1 (PH)	Patient compliance; guidance adherence	Compliance AND bandaging AND lymphedema; NOT filariasis	Patient compliance; lymphedema; bandages;
CM2 (GM)	Inflammatory arthritis	chiropractic AND arthritis	--
HF3 (EC)	Homeopathy	Homeopathy AND massage therapy	--
MF4 (JX)	Fibromyalgia	Fibromyalgia AND massage therapy	--
MF5	Pregnancy;	Pregnancy AND reflexology: pregnancy AND reflexology OR CAM	--
MM6	Osteopathy	osteopathy AND joints; viscera AND spinal pain;	
MF7	Lymph system	lymph system AND anatomy; lymph system AND anatomy NOT renal;	
MF8	Fibromyalgia	Fibromyalgia AND massage	Massage; patellar tendonitis; tendonitis; low back pain; joint manipulation; high velocity manipulation
MM9	Back-pain	Back pain AND massage therapy OR chiropractic; Back pain AND massage therapy OR chiropractic NOT drugs	Back pain; fibromyalgia; messo-therapy – hydro-therapy;
CF10	Visceral pain	Visceral pain NOT pelvic pain; visceral pain AND pelvic pain	
CM11	Chiropractic	Effectiveness AND chiropractic;	
CF12	Chiropractic	Strokes and chiropractic; acupuncture and Bells Palsy;	Acupuncture
CM13	Child obesity	Child obesity AND Canada*	
CM14	Lateral epicondilitus	lateral epicondilitus AND treatment’	
CM15	Orthopedic tests	Orthopedic tests AND shoulder OR rotator cuff	
CM16	Fibromyalgia	Fibromyalgia AND escorbin	
CM17	Dizziness	Dizziness AND symptoms	
CM18	Nutrition	nutrition AND disease prevention NOT longevity	
