# Supplementary material for: How Complementary and Alternative Medicine Practitioners Use PubMed
Source: J Med Internet Res. 2007 Jun 29;9(2):e19. doi: 10.2196/jmir.9.2.e19 (PMC1913941; doi:10.2196/jmir.9.2.e19)
Supplement: Supplementary file 3 [file jmir_v9i2e19_app3.pdf]

JMIR Multimedia Appendix 3 (www.jmir.org - Vol 9 Iss 2 e19)
Interview with MF1

Homepage description:
I: Ok, so lets start with a search from the homepage. Go to Pubmed.com
MF1: Ok, I am there.
I: Have you ever been here before?
MF1: Yes- many times.
I: We can start by doing a simple search. Anything you'd like to know more about?
MF1: Ok, I'd like to know more about umm, compliance. I am interested in the area of compliance. Umm, compliance with I'm sure there aren't any studies on this but anyway bandaging for lymphedema.
I: Ok, so that's a pretty complicated topic. One good way to search it would be to do a Boolean search. Do you know what that is?
MF1: Yes.
I: Ok, so will put in compliance and then we'll put in and bandage.
MF1: And the AND has to be capitals.
I: umm, I don't think so, but do it anyways, it couldn't hurt. And then put in 'Lymphedema'. And then hit go.
MF1: Yup.
I: So how may did you get?
MF1: I got 11.
I: Ok, so will you look through them and se if they are useful to you?
MF1: It's hard to tell just from the titles. But let's just see I'll get the abstract. Ok, the first one looks good. "Managing and promoting change".
I: so you find abstracts useful?
MF1: Yes, very.
I: Can you go back to the list for a second? I just want to point out a few things out. So, let me point out a few things you probably know already. The first is that you know that these are citations, not articles, right?
MF1: Yes.
I: and you know what a citation is?
MF1: Yes
I: Great well the little picture of the piece of paper represents the citation and you can click into it and arrive at details of the citation- if there is an abstract then there are little lines in the piece of paper icon. If articles are free full text there is a green bar in the icon…
MF1: Yes that's intuitive.
I: So you know the difference between an article that is free online and one that is available online but is not free?
MF1: Yes I look for the free ones.
I: Ok, so one interesting thing we can do is as we find research results that are interesting to you we can amalgamate them on the clipboard. And then send the citations to your email in box. Have you done this before?
MF1: I have.
I: Oh, ok. So click the checkbox and then hit send to clipboard. And let me know when you are done.
MT1 Ok, I am just clicking a few more off.

I: Actually if you could just go back to that initial abstract I want to hit related articles.
MF1: Yes, umm, ok, I got it clicked and where am I going to send it?
I: Ok, do you see where it says send to?
MF1: Yah, I do.
I: Ok well choose clipboard first.
MF1: Alright. Sorry, I usually send it my email.
I: That's ok, so go back to the abstract by Seymor. Now if you do enjoy reading stuff by Seymor, you can always click on Seymor and it'll take you back to other articles that he's written.
MF1: Right.
Related articles:
I: Or 'her'- since her name is Ellen. But now, I want you to hit related articles, to the side there, and I want to know what your thoughts are as to whether it pulls up a search list that reflects the initial abstract, that would be useful to you in other words. As they get useful check them off and send them to your clipboard.
MF1: Well the first one is the one by Seymor.
I: Yes the first one is always like the same it is the initial stepping stone.
MF1: Well these are all about ulcers.
I: Ok.
MF1: I didn't really… it's similar but it's not the same as lymphedema but I'll send it to my clipboard.
I: Well you don't have to send them if you don't want to see them.
MF1: No, no that's fine, I do.
I: Ok. So in terms of finding other articles that related to the first one, it took you in another direction. Is that true?
MF1: Umm, I don't know. I'd have to go back to read the Ellen Seymor. I didn't read the whole abstract. I'd have to go back and look at that. Because may be the leg club model was important to it in the first place.
I: Yah right it could be, right.
MF1: Rather than Lymphedema.
I: Yup.
MF1: So how do I get back there. ….
I: But at the same time it does talk about the reevaluation of the use of the long stretch bandages in favour of the short stretch – so it really does have something to do with bandaging.
MF1: Oh yes it does. It certainly is about bandaging and about increased patient compliance. So that's what I am interested in. So the related articles tool did have some application.

Books:
I: So have you ever looked for books before?
MF1: no
I: ok, see links to the right there?
MF1: yes
I: click on it and then on books.
MF1: Ok,
I: Do you see how some terms are underlined in blue?
MF1: yes

I: Well when you click on the term it takes you to books in which the term is mentioned.
MF1: oh, I see
I: So if you ever need a book on a subject then you find one this way. And note that we are in a books database.
MF1: well that 's interesting but I like articles because they are more current.
I: ok, good info. So you don't think you'll use the books function?
MF1: well it depends, but I have never really thought about it before.
I: Ok.

Linkout:
I: Ok, another one is the Linkout button. Click on links and you'll see another little menu come up. And you'll see another little button come up called links.
MF1: Ok yes.
I: Have you ever used this tool before?
MF1: I haven't.
I: Ok, it shows you exactly where you would get this article. So if its available for free where you would get it – in a library or for sale by the publishers. So if you fall in love with an article, if you click on Linkout you can see how to get it. In this case it looks to me like there is also a free supplement that is provided about leg injuries. That is not the text, the text is where it says full text. Do you see where it says that? If you click on that you get taken to the publishers web site.
MF1: Right.
I: And unfortunately this is not a free article. You need a code or ID or something to be able to log into it.
MF1: Ok, I am just going to look at the free one and see what they have. Hmm interesting, what's there.
I: you mean the Medline plus web site?
MF1: Yah.
I: Have you ever been to Medline plus?
MF1: umm, I think I have been to it some time, but not in any particular category of things.
I: It's a resource for less technical information about a lot medical issues and problems. It is basically for lay people.
MF1: Right.
I: So you can find stuff about all different sorts of things on this web site, and this info. Is basically gleaned from the info in Pubmed.
MF1: right.
I: So you can always do great searches in Medline plus, and most is free- but you won't get access to actual full text article,
MF1: No.
I: Is that what you are looking for? More full text articles?
MF1: I am, yes.
I: ok, so it's useful but it's not exactly what you are looking for.
MF1: No.
I: ok, well, go back to our Linkout window and click on libraries.
MF1: Ok, yes
I: And hit all
MF1: Oh yes

I: And here you have a list of three universities that do carry the article.
MF1: Uhh huh…
I: I'm not sure what your access would be but you might be able to get the article from one of these guys- form the libraries.. the point is if you saw McGill or Uof M here, you might be able to go to those libraries and get the article.
MF1: that is more, you wouldn't necessarily find this from the general page?
I: Well what you find form the general page (homepage) if there is a free full text then.
MF1: Yes, it'll show it on that page. And most of the time they aren't so …
I: Yes, and most of the time there is more than one point of access to things, but linkout will provide you the information of where to get the resources if you are confused or can't find it or it is at a library. It'll also take you to other resources associated with it.
MF1: right, right, ok.

Send to:
I: There's another thing I want to show you and that's the clipboard. We've clicked off a couple of article citations and we've saved them to the clipboard.
MF1: Yes, ok.
I: So let's go back and look at them.
MF1: Ok. I've got 5 here.
I: So part of using Pubmed is to organize the research in such a way that you can stay on top of it all because you might collect a lot of research while your doing your search , and you might want to sort through them before you send them to your computer.
MF1: Ok.
I: So this is where you would do that. Here you have the ability to keep your clipbaorad alive for 8 hours or more. You can save 500 citations up there. So after you sort through them and unclick the one you don't like, you just click on send to and then click on whatever: file, email, printer. So try file.
MF1: Ok.
I: So do you see what it does?
MF1: Not really.
I: It makes it look like a typewriter file- no formatting which is easier for you to save on to your computer.
MF1: Ok, so now I have to save it somewhere?
I: Yes, exactly.
MF1: I am used sending things to my email, but I am not used to putting them in a folder.
I: Ok, well, we'll do email after this. I just wanted to show that you don't always have to email things to yourself you can
MF1: I'm not so good at organizing my computer files. It's a waste of time when I can just email them to myself.
I: Ok, so then select email, and then type in your email address. You can write something into the box there to remind you what this search is.
MF1: Yah I have emailed myself before but normally I don't use the clipboard. I just send them without using the clipboard.
I: So do you think the clipboard would be useful to you?
MF1: Yes it is.
I: Ok good.
MF1: because as you say you can then look at it and decide whether or not the one you thought might be interesting, when you look at it against another one isn't so interesting.

I: Right. Exactly the point. Ok, so let's move on to the next one. There's a couple of tools that will let you refine your search.
MF1: Yes.

Mesh:
I: So we were actually quite lucky when we did the compliance search – we found a lot-11 is pretty good. Let's say there is a search you want to do and you don't know how to articulate, how it is talked about in the research literature. Click on MESH, on the blue navigation bar to the left. Have you done this before?
MF1: Yes – well I have taken the tutorial, and I've tried to use it, and I know it provides you with headings
I: Yah and what you should be thinking of MESH as is somewhere between a thesaurus and a dictionary.
MF1: yes.
I: so why don't we try to look up compliance and see what happens?
MF1: ok. I've got one of seven.
I: Ok, so here we've got the many different ways compliance is used. And you meant the first one
MF1: Patient compliance- and guidance adherence is another one.
I: So you mean both number 1 and number 5?
MF1: Possible, yes.
I: ok, well let's start with number 1.
MF1: Ok.
I: the most basic thing you can do with the term is put patient compliance in as your search term. So go back to Pubmed because now we are in the MESH database. And search patient compliance and lymhpedema or bandages.
MF1: right
I: So that's one way to use MESH that's very simple. And simple is good.
MF1: Right
I: So here there are some more entry terms and this is what we are really looking for, so it says …. Patient cooperation, family planning clinic attendance … etc, so let's use some of these- compliance patient, patient cooperation and let's go back to Pubmed and look under that – does that make sense?
MF1: Yes but how do I get back to Pubmed?
I: great question. Do you see where it says Search and Mesh?
MF1: Yes.
I: Drop down that tab and select Pubmed – go upwards.
MF1: It's not under "P" – its up at the top.
I: Ok so what should we look up?
MF1: Patient compliance and Lymphedema because otherwise we'll get ulcers… I think we got more or less the same seven.
I: How about we try cooperation?
MF1: No items found. Another one is adherence and I don't know if that one works.,..

Combining through history:
I: Well, let's move on to try something else. The first thing I want you to do is write patient compliance in the search bar and then search, ok?
MF1: yes

I: And let me know what you got for this one.
MF1: 31165.
I: ok, now erase that, and write Lymphedema, and then search.
MF1: ok
I: and now erase that and put in bandages and search.
MF1: Ok. Actually let me change that to complex decongestive therapy.
I: Ok.
MF1: 29.
I: Not much.
MF1: But that's the complete therapy bandaging is only part of it.
I: Now go to history and …
MF1: Yah, I do this all the time.
I: Oh... Ok.
MF1: And we combine them, right?
I: Right its kind of like doing a Boolean search.
MF1: Yes, oh I find this very useful. I don't do Boolean nearly as much as doing this. Ok so 12 and 13 and number 14. (she combines them). So again I get 7.
I: Well, it could be that there are only 7 articles about your topic. Ok. Well, have you ever used limits?
MF1: I have used limits with dates mainly. And if I am searching for a particular author. That's about it, and may be English only or things like that.
I: Ok, we click on it and we'll just go through the page. Why don't we put a few on and we'll search only for Lymphedema?
MF1: Ok.
I: So, which ones should we put?
MF1: well, what about abstracts only and …
I: And how about English?
MF1: Ok,
I: Ok and go.
MF1: Well there is one here on maintenance therapy that looks good.
I: We can now search for decongestive therapies within this list.
MF1: well why don't we put therapy?
I: I think that's a great idea. Head to 'history' and go to number 12.
MF1: Well we really just need to put in number 17 and therapy, right?
I: Oh mine is number 12 because you did more searches than me. So what did you say?
MF1: therapy. So we enter number 17 AND therapy. So now we get 118.
I: How do those look?
MF1: Well, a lot of them are about Filariasis which I am not interested in, and I don't know how to eliminate that.
I: Ok so you know how to eliminate that?
MF1: No
I: In your search field up top write NOT Filariasis
MF1: Ok so now we are down to 85. (She looks through the list). Yes I can see that therapy is being used probably as a medical term rather than – you know like chemo therapy or radiation therapy, rather than decongestive therapy. So we could take this one and we could say AND decongestive therapy.
I: Yah, put decongestive before the bracket…
MF1: No I didn't put brackets.

I: Oh, mine did it automatically.
MF1: Ok well now I have only go three. I think the original search was better – those first seven, there is one or two that might be useful. They aren't exactly what I am interested in but they would be possibly useful pointers.
I: Except that you can't get them.
MF1: Yes, its too bad. It's the publisher's stranglehold on the universities.
I: yes well … so, your search skills are really really strong, and I am not sure I helped you out too much, but you certainly helped me out and I really want to thank you for that.
MF1: What do you mean, how did I help you out?
I: Well I really liked your comment about the first search list being more relevant, about needing access to the research, and I liked how even though we tried to make our search strategy very complicated, I liked how it turned out the most basic one was useful.
MF1: Well that's partly because I am quite familiar with this literature. And I have an automatic referral for NCBI every so often about updates of new research in this area.
I: oh that's good news too.
MF1: In this area, and for massage therapy generally, so as I say I can spot a new article, I should know the literature with my MA topic so.
I: Well that's great.
MF1: So how is this research going?
I: Well it's coming along – it is interesting to see the different types of people who can benefit from Pubmed.
MF1: So, I am interested if other people have complained about the lack of free access, or lack of access or am I the only one complaining?
I: Well, I wouldn't say that you are complaining, it's more like you are making a good point. And a lot of people are unaware of what they can get their hands on and what they cannot. Some don't know all of the policies behind why some are free and some are not and so there is a lot of confusion out there. So the main issue is that people aren't asking the right questions, they don't feel entitled to see the literature.
MF1: And do many of them have access to a medical library?
I: Some from the CMCC do have access.
MF1: oh yes, they have an excellent library I think.
I: they do, but when they graduate they won't have that access anymore.
MF1: No, that's the problem you see. It's very hard. I don't know what happens with medical practitioners once they've …
I: Yah, it's a good question. I wonder if the CMA has an institutional membership with publishers or something…possibly when you belong to a professional association it is easier to get subscriptions
MF1: well professional associations that have a lot of money may be, but for chiropractors and massage therapists they don't have that access- they don't have the money.
I: So what happened with the Centennial College course?
MF1: It was a good course.
I: could you get access through Centennial college?
MF1: Yes, well when I was taking the course I could – they had excellent access, and when I was doing my MA at Memorial, I had excellent access and also at Memorial you could go in and use the computers in the health sciences library.
I: Are you not able to do that at McGill?
MF1: They control it very, very, very tightly. I went in and asked if I could use the

computer. I already had my list of articles that I knew I wanted, I was pretty sure I would want to print them. I wasn't searching, I had 6 articles I wanted to see, and they let me do that. So having done it once now I know how to do it, so I just have to find the right time when it's not busy because the medical school is very busy and I understand that libraries are for the student but they are also for the public and some universities are much more open to public access then others but I also know that publishers make universities sign these contracts which any person or member of the public could enter the library and read a journal or read a textbook. But if none of the journals are in hard copy then you have no access unless you can get on the computers. And it's a backwards step in my view.
I: Well that's great. Great information. If you have any questions regarding Pubmed do feel free to email me and I can try to answer them. And I really appreciate your points of view and responses and they will come in handy! So thanks!
MF1: thank you.
I: Bye
MF1: Bye.

Follow up Email response:
PubMed use is pretty good. After full access to publications, however, it is a little frustrating to have access only to abstracts. The contracts that the academic institutions (are forced to) sign with the publishers, limiting access to students and faculty only, mean less access for the public. When hard copies were available, anyone could read, digest, copy relative sections etc. Now, depending on the institutional policy, it may be difficult even to get access to view a full text. Needless to say the costs of individual articles is exorbitant.

Interview with CM2:
I: So, should I explain how the homepage works, or is laid out?
CM2: Actually, I have been here before, and have already done searches. So I have seen this page already.
I: What should we look up?
CM2: How about 'Inflammatory arthritis'?

(Several citations came up including one with an icon indicating free full text availability)
I: Do you know what the icon signifies?
CM2: I think it means that a full text is available.
I: Yes, but it is available. How did you know that?
CM2: I intuited that this was what it indicated.

Boolean:
I: Have you ever heard of a Boolean search before?
CM2: I might've heard Boolean before, but I don't really know what it means. I need a refresher…
I: Basically, it is a way of making your search more specific, or drilling down better, by using language as opposed to the features and functions of the database.
CM2: Ok, I don't really get it.
I: So, instead of just searching 'inflammatory arthritis', you'll put in 'inflammatory AND arthritis'. Do you see how that limits things?
CM2: I don't really see how that's much different than just inflammatory arthritis.
I: Well, when you are putting the AND in there you are telling the database to look for this AND that – it may not make much of difference with this example. But picture a Ven

diagram, when you put two thing together, it's the space in common that is your search. Can you picture that?
CM2: Oh yes, I get it now.
I: Boolean operators include AND, OR, NOT. And sometime they widen the search and sometimes they limit it. Like if you did Chiropratic AND arthritis NOT knee pain. See how it would limit the search?
CM2: Yeh, I get it.
(We try chiropractic AND arthritis and pull up a number of articles.)
CM2: That's really great. I will use the Boolean search methods even when using the different features of the database. This is incredibly important to know, use and understand. It's like the building blocks of searches. I've got to practice that one afterwards.
I: Exactly, you could use this technique in all online searching- like Google for example.

Abstract:
[The candidate read over the abstracts and made a decision as to whether or not the article was useful to him. With this particular text he was not interested in reading the full article.]
I: Does the abstract give you enough information? What does it tell you?
CM2: The abstract gives enough information to help me make the decision as to whether I would want to read an article. So an abstract is really useful. If I felt the abstract was interesting, I would definitely need the full text to actually find the article useful to my work. An abstract only provides enough information to pass the first screening test.
I: What do you think overall about the results, the research that we found?
CM2: This information is not what I am looking for. It is too technical, too specialized.

(We clicked into a few other article citations. Two different types of buttons came up with the different abstracts. One indicated that an article was online but not free.)
I: Ok, so see here that the article is available online, but it is not free full text. It just means that it is in electronic format, and you can access it online. Should we take a look?
CM2: Sure thing.
(We go to the publisher's homepage where the article is located)
CM2: Ok, so what do we have here …
I: Well, you have to register for it to get access to it.
CM2: Ok, no, I am not interested in doing that.
I: Ok, should we try a different one. There is a free full text..
CM2: Ok
(We click back to the free full text citation.)
I: Ok, so, again, a green bar indicated that it is free online. Let's click into the citation.
CM2: Ok
I: Ok, and now you see the button …
CM2: Yup … this incredible it is so easy. I wish I could just finish reading the article right now ….

Related articles:
I: Ok, now lets try using the related articles link to the side over there.
CM2: I've used related articles before in other databases.
I: Oh, great. So what do you think about it?
CM2: As long as the initial article is relevant my needs, this feature would be really useful. But I do like it- it could be really useful.
(Little time was spent on this tool as it was something the candidate had done before in

other databases. He had used a similar tool before, and already knew that he liked it.)

Books:
I: Ok, do you see how there is a something called links next to related articles?
CM2: Yup.
I: Ok, click into it and select books.
CM2: Ok, I have never seen this before.
I: Ok, notice how near the search field where it says search in, it now says books?
CM2: Yup.
I: Ok, so we are not in Pubmed anymore, but in something called 'Bookshelf'. And all of the terms highlighted in here are referred to in books that are in this database.
CM2: Ok, so, this is a database with books in it?
I: Well, actually this is a database with information about books. There are abstracts and tables of contents, but not books. But your search terms were mentioned in these books. So the books are even necessarily about your search terms, but are mentioned.
CM2: ok.. (He browses)
I: Take your time to look through them … what do you think?
CM2: I don't think I'll actually read any of these books. But it is good to know what is out there I just think I would rather have articles.
I: Fair enough.

Linkout:
I: Did you see there was another option when you clicked on books? It is called link out. So head back to the citation, out of books, and choose that one.
CM2: Ok, got it.
I: So here we have a place to go to in case you do find that perfect article, and you want to try to get access to it. So you have the online options, which we looked at before, but you also have a list of libraries that have it. If you scroll down to libraries. So it's like a map that in essence tells you where the article is.
(He looks around for a few minutes).
I: What do you think about it?
CM2: I really think this great. It is a useful tool because sometimes all of the information about the citation can be difficult to understand. Now that I get what this is for, it makes perfect sense. I might click into it if I really liked the looks of an article in order to ensure I know how to get it.
I: You might even be able to get it from a library near by or something, like UofT.
CM2: Yah – I could do that.

Limits:
I: Ok, now let's try going to another tool to help you refine your search. So you were unhappy with the initial results, you found them too technical. So we can try to be more strategic with this one by limiting the types of articles and journals we look at. Click on limits- do you see it there?
CM2: Got it
I: Ok, so this limits the meta – information, for example the language of the article"
CM2: Ok.
I: So, what limits should we put on? We have a choice of different ones.
CM2: Ok, lets try core clinical journals, English, and human.
I: Would you like to limit to age range of the subjects?
CM2: Ok, I think I'll do over 18.
I: Ok, so take a minute and look through the list.
CM2: Can I take a limit off?

I: Sure you can do whatever you want, take your time … which one are you going to take off?
CM2: The age range.
(Much time was spent on playing around with the results given by placing different limits on search subjects.)
CM2: I really like this one because I can get at exactly what I want. But sometimes we don't get any hits, so I think I need to play around with it.
(Looks through the list some more)
CM2: This would be a good time to begin using related articles because of how specific the searches are once you place limits on them.
I: Great comment – that makes sense.

Send to functions:
I: Ok, so one thing that you can do in Pubmed send things to your email inbox. Do you see the send to function up top?
CM2: Yup
I: Ok, so click on it an scroll down …have you ever emailed search results, citations to your self?
CM2: I've done this before when I have found research on the web. I think its great it is very easy, and I like to keep things in my inbox. This is a great tool that I would use.
I: Well there is another way to do something similar. You can save things up into a clipboard in Pubmed, and kind of compile a list of search results. Have you ever done this before?
CM2: No, I have done email before, and I copy and paste research results when I do searches.
I: Ok, do you see the little box next to the citation?
CM2: Yes, got it
I: Select it
CM2: Ok
I: Click send to and scroll down and click on clipboard
CM2: Ok.
I: So the citation that you selected is now saved on to your clipboard. But you then have to save that somewhere. You can't just leave it there. So now scroll down and hit send to text and do you see.
CM2: Yah it looks different.
I: Right it has no formatting so you can save it on to your computer.
CM2: Ok, so this features (clipboard) is useful – it is so much easier than doing all that cutting and pasting. I think I'll use the clipboard feature, but not sure about the send to text feature. I find it a little confusing. I lesson on file management for my computer. May be then I could use the text feature better. I think I would send things to my email instead.

Full text:
I: Can you tell me what you think about getting access to the research? Would you be able to work from abstracts and citations, or do you think you need to actually read the articles?
CM2: It is incredibly important to have access to the full text of the article. I would even pay to access a full text article.

Future use:
I: What tools do you see yourself using the most in the future?
CM2: Hmm, I guess it would be the send to email to send my search results.

I: What do you think the most valuable feature is?
CM2: I really like related articles- I saw it in the other database and it is useful because it brings me to things that I would probably not have found otherwise – outside my own searching.

Reasons to use Pubmed
I: Can you describe how you see yourself using Pubmed?
CM2: I think I'll use it to get research, to get on the fly research to answer questions. But I wouldn't send my patients to use the database or conduct searches on this database after a consult with them because I think it would be too complicated. I would not refer them to Pubmed to find their own research.

General comments:
I: Any general comments about the session, or Pubmed, anything you would like to add?
CM2: After this I feel comfortable using Pubmed and am looking forward to using the new features I have learned.

Follow up email response:
Yes I have used Pubmed since our last conversation. I use it on a weekly basis. I use it to find information for patients and for myself. I am in the process of developing an acupuncture-related web site and writing a book on health-related topics. I have not purchased any articles (although I have been tempted), I tend to look at the full text articles and I have used the links (LinkOut and Books) with success. I like the way that it saves my limits so I don't have to reset them every time I change my topic search. I often use the related article section as well. I can't say that I have stayed away from any features in particular, perhaps this is because I am comfortable using the features that I mentioned above.

<u>Interview with H3:</u>

Homepage:
I: Let's start from the homepage. Type in www.pubmed.com. And notice how when we get the page the URL changes. Just so you don't get too startled when you see that- it gets re-directed. But all you have to remember iswww.pubmed.com.
H3: Ok, sounds good.
I: So what would you like to search?
H3: umm, can we do homeopathy?
I: Ok, so just enter it into the search field there, the white box underneath the navigation bar. And just before you hit go, let me just explain a couple of things.
H3: Ok
I: Do you see how there is a black strip above the search bar?
H3: yes
I: Ok, and there is white writing in the strip. Well those are actually other databases that are more specialized that are part of Pubmed. You can click into them. And do you see how it says 'search in' and then it says Pubmed in a drop down format?
H3: yes
I: Well you can pull that down and go into those other databases. Its good so that you know where you are. Search in Pubmed automatically comes up though.

H3: Ok. Great.
I: So now hit go. (3000 results came up). Ok so here we have our search results, and I am just going to quickly go over this. Here is a list of what we call citations. That is every article and all of the information associated with the article is called a citation. That includes the authors, the journal name, the abstract etc. Let's click into one citation. Do you know all this already?
H3: I know a little bit about this but I do need a refresher. It's stuff that I know but not really- like I didn't know the name citation.
I: ok, great. Well in this citation we have the authors names, the journal article, the abstract, and do you see the little icon to the side there? It looks like a piH3e of paper?
H3: yah
I: Ok, that's a pretty important symbol. If it has lines in it means there is an abstract associated with the paper. So, form the search list you can click on it, and it'll take you into the citation.
H3: Ok, got it.
I: So, do you see how author's names are underlined in blue? That means that you can click on them, and it'll take you to other articles they have written. So, if you really like the research that one particular author does, this is one way to find more of what he has written.
H3: Oh really? That's neat.
I: Yes. Ok, now let's go back to our search list. And I just want to show you one more quick little thing before we move on to the next thing. Do you see number 13? What's different about the little symbol?
H3: It has a green bar?
I: Yes – that actually means something important. It means that we can get access to the article for free. Click into the citation.
(We do that)
I: Ok, so we have the regular citation, but we also have another button available to us. Do you see it says available for free?
H3: Yes.
I: Ok, so, if we click into it, it'll take us to another web site- the journal web site.
(We do this)
H3: Oh wow! Ok, so I want to find lots of articles with the green bar?
I: Right- well you want to find citations with a green bar. How does this article look to you? Useful? Would you want to read it? Take a minute to look it over. This web site has similar features to Pubmed …
H3: Yah, it looks pretty good. (She reads it over). I would like to read it.
I: Ok, well, how about this, let's send the citation to you via email and then you can read it later.
H3: Sounds great.
I: Head back to the search list, and do you see the little checkbox … click into it.
H3: Ok, got it.
I: Now do you see where it says send to up at the top menu bar?
H3: umm, … ok, now I see it.
I: pull it down and select email. Then you'll put your email address in the field at the bottom. And if you want a little note to yourself about what the article is about, for you references. Or you can leave that blank.
H3: So what will it send me then?

I: You can choose what you want to see, but if you don't choose it'll automatically send you the citation- author, title, journal, and I believe it send the abstract too.
H3: So then how do I get the article?
I: you can find the citation in Pubmed again, and then click on that button.
H3; oh, I see. Hmm, I would rather have the article. Can I do that?
I: Well you can look up the journal web site after our lesson and find the article that way. It doesn't have to be through Pubmed since this a free article. But having the information about the article will allow you to find it in the future.
H3: Ok, I get it. So now that's in my inbox.
I: It is indeed.
H3: Oh, well that is really handy. I knew about sending stuff to the email before, but now I really understand why it's important.
I: Right, as long as you know something about the article- you can find it again. That's why citations are important. Let's look at another way to keep information. Instead of sending thins to email, you can save it as a text file. So, select the article
H3: Ok.
I: And go back to where it says send to and find text.
H3: oh, so now this is on my computer? How do I find it again?
I: Well, actually, it's still just a web page, but with all of the formatting stripped. So you can easily save this to your computer and then refer to it later on.
H3: I'm not really sure what I would do this. I would rather send thing sot my email inbox and then find the articles.
I: Ok, one more way to save things and organize information is to save things to a clipboard.
H3: Where is the clipboard?
I: Well, the process is the same, but instead of sending it to text or email, you would choose …
H3: Clipboard. I see it.
I: But the advantage here is that you can select quite a few articles by checking into more than one box. And then you can save them all to the clipboard. And afterwards go through them and see which ones you want.
H3: Ok, so select a bunch, and then send to clipboard.
I: Right.
H3: And then how do I go back to clipboard?
I: Ok, see the tabs at top? Click on the one that says clipboard.
H3: Oh, I see them. But then what do I do with them?
I: You could send them to your email.
H3: This seems a little complicated. I think I would stick to using just the email function finding articles as I go.
I: Ok, that is great feedback. Thank you. Now let's try something else.

Boolean Search:
I: So you see what a got a huge amount of results when we did our search for homeopathy. One thing we want to do is to try to actually lower the amount of results we get when we search – we want to refine the search a little bit.
H3: Ok.
I: one way to do that is by doing what we call a Boolean search. Have you heard of this before?

H3: Actually, I do know Boolean; When you use AND in your searches.
I: Yes, along with NOT and OR.
H3: Yes.
I: So what should we search for: homeopathy AND what?
H3: Hmm, good question. Massage therapy?
I: Ok, sounds good. SO just type it in. … Ok, so do you see how it provides fewer research results?
H3: Yes I really do.
I: Will you look through the list and check to see if these look like good articles?
H3: Ok (a few minutes pass). Yes these articles look great. I would want to read all of them.
I: Ok, great. Well, since we are starting off with a good list, a useful list, its a good time to learn another tool called related articles.
H3: Ok.
I: Have you ever done this before?
H3: I have used a similar thing in another database.
I: Ok, well its good start with an article that you like, so it'll call up articles that are related to it.
H3: Ok how about number 5?
I: Sounds good. So click it and then on related articles and then let me know whether what comes up is useful. Note that the first article that comes up was the original article. Give yourself a few minutes to look things over.
H3: Oh, interesting. … yes these look really great. …I could spend hours just seeing what comes up from this feature after starting from one search.
I: You could email them to yourself. But I think we are running out of time. So, can we try another feature?
H3: Sure thing.
I: But all in all, did you like that one? Did you find it useful… like do you think you'll use it again?
H3: Yes, definitely, especially if I am browsing around.
I: Ok, great feedback. Now, are you in the citation?
H3: yes.
I: Ok, do you see right next to related articles it says links?
H3: Yes.

Books:
I: Ok, click it, and then scroll to books, and select books.
H3: Ok.
I: Ok, do you see how there are a lot of words highlighted in blue?
H3: Well that means that those words can be found in a book. Do you see how now we are in the books database, look up near the search field?
H3: Oh yes.
I: Ok, so this books database is a part of Pubmed. It is a database that contains books about different themes as opposed to journal articles. So, click on one of the search terms that is highlighted. So try complementary medicine.
H3: Ok.
I: Ok, so now we see complementary medicine is mentioned or cited in these books. In the first book. HSAT complementary medicine is mentioned 125 times.

H3: Ok.
I: Now click on the first one.
H3: On the picture?
I: Well if you click on the cover, you will get information about the book. If you click on the 125 items, you will see where complimentary medicine is mentioned. So click on the items.
H3: Ok.
I: Now just have a look at this list of information. … This is how complementary medicine is mentioned in this book HSTAT.
H3: Oh, I see,
I: take your time in looking at it.
(A few minutes pass)
I: So basically if you were looking for extensive information about a subject mater, like complementary medicine this is how you might go about finding books on the subject. Like a textbook or something, just to give you an idea about how you would this…
H3: Ok
I: What do you think about this?
H3: I see why it would be useful – but I don't think I would ever really use it for myself. It is interesting to see whether there are whole books on a particular subject. But I am not sure I would really use it that often. I like journal articles because they are more specific.
I: Fair enough. Ok, well, we have just a few more things to cover. Is that ok?
H3: Definitely, this is helpful, I am really learning a lot.
I: Great! Ok, hit a few times and we'll go back the citation we were working form.
H3: Ok.
I: Ok, go back to links where Books was.
H3: Ok.
Linkout
I: And now choose Linkout.
H3: Ok.
I: So here we have basically a little map or guide to where this article can be found- how to get your hands on it.
H3: umm, ok.
I: Ok, so remember, Pubmed does not always provide access to the full text of the articles. It provides information about journals… and then it is up to you to get them, like at your library or something.
H3: Right – so this tells me how to get them? But what about those links to journal web sites?
I: You mean when we looked at a free one – with a green bar?
H3: Yes. I don't remember doing this to get to the article?
I: That's a great question actually. The link for the full text articles can usually be found in more than one place- right away when you click into the citation, and also from link out. But when the article is not free full text it's a little different.
H3: Ok – so go to Linkout when the article is not free to find it.
I: Well, kind of. First of all, not all articles are electronic. A lot of the new ones, but not all of the less recent ones. So, if an article is located at a library, you can go to Linkout and do you see where it says libraries?
H3: Yes
I: You can look up your library and see if they have the journal. So, just click into any of

the libraries- actually check if they have UVic or UBC…
H3: Ok – so ..
I: Go to ‘U’ and it is an alphabetical list.
H3: I don’t see them.
I: How about UofT?
H3: Oh yes- it says electronic.
I: Ok, so then UofT has the electronic version of the paper. So if you were a student at UofT you could probably get it through your library.
H3: Well, too bad I’m not.
I: Well, if you yourself wanted it you could get it too – you would have to pay for it. I’ll show you how to do it from Linkout.
H3: ok.
I: So click out of libraries, and do you see where it says Elsevier Science and then full text?
H3: Yes.
I: Ok, click on that and it’ll take you to the publisher’s web site.
H3: Ok … but it says you need a log in and password
I: right you need to buy access – $30.00.
H3: I see – so I would need to put in my credit card information?
I: Exactly. Do you think you would even buy an article?
H3: Well, it would depend on the article, and why I was using. It would also depend on whether I could find other articles that are free. So, if I was desperate to find information and couldn’t find anything, then I might actually buy it. It depends on the situation.
I: Ok, good to know. Now, let’s just go back to Linkout- is it clear why you would use it?
H3: I think so- to get access to the articles.
I: Right, and what do you think about it?
H3: Well, if I needed access I would use it. I think I’ll look for mostly free articles so I could actually read them, so I might not even use Linkout, or need it. But I would use it if I found and non-free article and needed to get to it.
I: ok, great. Thanks.
Limits:
I: Ok, so now lets move on to another tool, that has more to do with searching than about access.
H3: OK.
I: Limits. Do you see where it says limits up at the top?
H3: Yup. I have done this once before.
I: Ok. Well, do you mind if I explain anyways?
H3: No- it will probably help me anyways.
I: Ok, great. So what we have here are options to limit our search. So, even before putting your search terms in you could stop and think, and say, hey, I want to find articles that only have abstracts with them, or I only want articles that are in English ..or
H3: Ok, so you click off the way you want to limit your search.
I: Right, and then when you perform your search you get only items with those criteria.
H3: So what do we have here …
I: Ok, so take a look at the different options for a second… but do remember that the more limits you put on, most likely the fewer results you would find.
H3: Ok, I am clicking on ‘items with abstracts only’ – I wouldn’t read a paper without an abstract, ‘English’ and ‘human’. I also want to choose editorials, as well as ‘clinical

trials', but I am not sure if that is possible,
I: Hmm, I don't think so. You can only click off one at a time.
H3: Ok, well, that's good enough for me.
I: Ok, and now we do our search - let's stick just to complementary medicine though
H3: Ok, so just put complementary medicine in the box.
I: Right … so when you get the search list, just take a moment to look it over, and tell me whether or not you think the list is good- whether you want to read the articles or not, and if limits, basically worked well for you.
(some time passes)
H3: Well, we got 78 articles here. They do look useful. Most of them I would want to read. … this looks like a good list and there aren't hundreds of articles to wade through. I guess that's because of the limits.
I: So what do you think of limits? Would you use them again?
H3: I would. I think that I would check of certain things at the beginning of my searches – like only items with abstracts – and then do whatever search I wanted. Yes- it definitely seems useful.
I: And just do you know, you can leave limits on for all of your searching- you don't have to re-check them off when you do different searches.
H3: Oh, that's good news…
History:
I: Ok, last tool to show you: history.
H3: ok.
I: Up at the top, click on history, and what you'll see is record of all of your past searches.
H3: Oh, neat.
I: Furthermore, you can see what time you did your search, and how many results you got. You can also click on the results number, pull up the actual search list.
H3: Wow. That's amazing. If I ever want to go back to what I did, this is how. It's like you don't have to write anything down because it is all recorded automatically. This is really, really great.
I: Ok, so take your time to look through it al little bit if you want.
But that's all I wanted to show you today. Any questions?
H3: Not really.
I: Can you name your favourite tool?
H3: hmm, I am not sure- I think it might wither be history or related articles…
I: Ok, thanks.

Follow up email:
I have used Pubmed on occasion (I am not practicing as a Naturopath full time, as I have a young child). I must admit, however, that I often learn about research studies from medical news sources (eg. medical post,), and access them through links provided in the article versus searching Pubmed. There is sooooo much information out there, and I find it hard to find time to access it all!

Interview with MF4:

Homepage:
I: Ok, so have you ever used Pubmed before?
MF4: Yes, but I found I wasn't getting the stuff that I wanted to get.
I: Ok, so let's go to the home page. Go to µwww.pubmed.com§.

JC: Ok. I am here.
I: Ok, so do you know what the search field is?
MF4: Yes, the white bar. I have done searches before.
I: Ok, so let's do a search. What for?
MF4: How about Fibromyalgia.
I: Sure. How do we spell that?
MF4: Fibromyalgia.
I: Great thanks. Ok, we got quite a few results. Do you know what the little icon that looks like a piece of paper symbolizes?
MF4: umm, we get to he information about the article?
I: Right- the citation. And depending on if there is a green or orange bar there, it shows that we can get the article for free.
MF4: Ok, yes I have seen that before in this database. I know to look for the green in the icon.
I: Ok, let's take a look at the information. So click the icon. And here we have the title of the article, the authors, the journal, and sometimes or actually most of the time the authors are in blue which means…
MF4: Yes, I know you can click on them and it brings you to articles they wrote.
I: right. Ok, great …
MF4: I know all of this stuff already so…
I: Ok, so let's move on to something a little more complicated. Have you ever heard of Boolean searches?
MF4: Yes. But I'm not that good at them.
I: Ok let's practice, and I think that it'll be very helpful for a search on fibromyalgia. By the way, what is Fibromyalgia?
MF4: It is very painful. When patients have chronic pain and stiffness in their muscles. It makes them tired all of the time. It's hard for them to move.
I: Oh, sounds terrible.
MF4: It is …
I: Ok, well let's try to get fewer results by limiting our search topic using Boolean. With Boolean you use the words AND, OR and NOT to limit or expand your topic. So, if we were to do Fibromylagia and massage therapy, what do you think would happen?
MF4: umm… Ok, it will make our search list smaller.
I: exactly, very good. Ok, what if we added NOT, let's say, arthritis?
MF4: umm, I'm not sure.
I: We would get even smaller search results. Adding NOT would limit it further.
MF4: Oh, ok.
I: We could expand it our topic by adding OR. Let's say Fibromyalgia AND massage therapy or chiropractic. This would mean it would search for massage therapy and chiropractic as the therapies so you are providing another option.
MF4: Ok.
I: Ok, let's try FB and MT. Sound good?
MF4: Yes.
(17 articles came up.)
I: Pretty good. Can you look through and let me know what you think?
MF4: Sure.
I: Take your time.
MF4: Ok. They look really interesting. I would want to see them more.
I: Ok, do you see the icon without the lines in it?
MF4: Yes.
I: That means there is no abstract. Would you want to read that one?
MF4: Well, not really I didn't even look at that one really.

I: Ok, so you think an abstract is important?
MF4: It gives me an idea if I want to read an article or not. I need it. I usually ignore the ones that don't have an abstract.
I: Ok, let's try to find more articles on this topic. DO you see the related articles button there to the side?
MF4: I have done this before.
I: Ok, well can you click it and let me know if the articles it calls up look interesting?
MF4: Ok. [looks over new list] This list makes more sense to me than the original list that came up.

Sending to email and clipboard:
I: Ok, great. How about you click on a few of the little boxes, and we'll practice sending things over email?
MF4: Ok.
I: Ok, so click into the little box near the citation.
MF4: Which citation?
I: Anyone that you want; one that you want to read.
MF4: Ok, how about number 12?
[It is free full text]
I: Great idea. But you know you can select more than one. Click the whole list if you want to …
MF4: But I am not sending the articles right?
I: Right. Only the information about the articles, so you can find them later on.
MF4: Ok, now what?
I: Ok, go to send to up at the top, and click on it so the menu comes down.
MF4: Ok.
I: Ok, and then select send to email.
MF4: Ok, now I put my email address in?
I: Exactly.
MF4: Should I put in more information?
I: Well, sometime you can put in like when you did the search or some way to recognize which search it is. But it's not necessary. It is to just stay organized.
MF4: Ok.
I: And then just hit send. Ok, so you see how it says the email was sent?
MF4: Yes.
I: Ok, let's try another way to send. We go back at our search list.
MF4: Ok.
I: And are your boxes still checked off?
MF4: yes.
I: Ok, now go back to send to and select clipboard.
MF4: Ok.
I: So now all of the citations are saved in what's a called a clipboard, and you can keep on adding them and check them.
MF4: How do I find the clipboard again?
I: You click on the top, there is a tab that says clipboard and the citations go there when you click them off.
MF4: So how long do they stay here? Oh I see what it says.
I: So, what do you think about that one?
MF4: This is much better than writing everything down by hand. I think I will use the clipboard function especially when I am going through related articles so I can save what is good to there.

I: There is another way to do this. Go to send to and select text.
MF4: Ok. Oh, I see…
I: So it is easier to save this way. You can save it on to your computer in a special Pubmed file.
MF4: Yes, that is definitely very useful. It is easier.
I: Ok, let's move on to something else. Click into a citation.
MF4: Anyone?
I: Yes, one that you like. And tell me which one it is.
MF4: ok, well why not number 12.
I: Great idea. Ok, now do you see to the right there is something called links?
MF4: Yes.
I: Click it and select books and tell me what you see.

Books database:
MF4: Ok, everything is underlined.
I: Ok, basically we have moved out of Pubmed into a different part of the database called books.
MF4: Ok.
I: So, everything that is underlined is mentioned in a book, so click on complementary medicine and see this means it was mentioned in these books this many times. 47.
MF4: Interesting. I was once looking at one point for a book on Fibromyalgia and I was looking around and couldn't find anything. This is very useful to me- but it would depend on what I am looking for…
I: Ok, great. Now let's try another tool. Hit back, and let's get back to our citation. Number 12.
MF4: Ok.

Linkout:
I: Ok and go back to links and choose Linkout.
MF4: Ok.
I: Ok, so what do you see?
MF4: I am not sure.
I: Ok, well here is basically a quick and easy way to get to an article. Its like a roadmap to the where the articles are, either in paper or electronically. Which schools carry them.
MF4: Ok.
I: So, this article is free and easy to get to through Pubmed, but sometimes they aren't so easy. See the link called libraries?
MF4: Yes.
I: Ok, it is a list of libraries that have this article. I'm not sure if Centennial will be included on it, but if you have access to a library then this might be helpful. But there is a also a clear link to where you can get the article.
MF4: Its true, when I was doing the course at Centennial, every time I used PubMed I found the abstract, but I tried to get the article. I couldn't get it and I had to go back onto Centennial's database to find it – so I could see why this is useful.
Limits:
I: Exactly, wonderful. So, let's move on to something else. Have you ever heard of limits before?
MF4: I think so; we did it on the other databases.
I: Ok, well let's go over it anyways.
MF4: Ok.
I: So do you see where it says limits at the top there?
MF4: yes

I: Ok, click into it. And here we have a list of options as which when chosen will help to limit or refine our search. It is kind of similar to Boolean in this respect.
MF4: Ok.
I: Well, one great limit is to limit the language. So you could choose to search only articles in English.
MF4: Ok
I: And also, let's say you're interested in searching a particular group of journals like complementary medicine journals. Do you see there is a selection?
MF4: Well, can I put core clinical journals?
I: Sure thing. And are you interested in women more than men? Because you can choose gender…
MF4: Not really.
I: How about a particular age group?
MF4: Not really – I guess we can choose over 18.
I: then there is also the choice about when the article is published. Some people are only interested in the most recent articles that have been published.
MF4: well, I just want to find any research.
I: Yah, it might be too limiting. Ok, well, let's do it then.
MF4: Ok.
(we search massage therapy and Fibromyalgia)
I: Not bad. Ok, will you look through the list and tell me whether you think it's a good list?
MF4: Ok. Yah, it looks good. Here is a useful article for me – it is about chiropractors and therapists working together. I would definitely read this
I: Great.
(more time passes).
I: So do you think you would use limits when searching?
MF4: Yah, its useful- it gets rid of all the articles you don't want. I find when I am doing Boolean searches [to drill down] I find I have to change my words and play around with them. This helps a lot.

History:
I: Wonderful. That's great feedback. Ok there are just a few more things for me to go through with you. Do you see where it says history, up at the top?
MF4: Yes.
I: Click on it.
MF4: Ok.
I: So, here you have a list of all of the things that you have done on Pubmed today. Yours searches, related articles, etc… everything has been logged. It's useful in case you forget something that you did, or you want to call up an old search list again. So, just have a look at it for a minute, and then let me know what you think.
MF4: It is interesting to see my past searches, but I think I will always want to conduct new searches and do not think I will pay much attention to what I did in the past?
I: Ok, great. Well, I think we are about done. But I am not in a rush or anything. Do you have any questions to ask me?
MF4: Not really. But this was useful. I have something to play with when I am using PubMed.
I: Ok great. Well, if you have any questions please do not hesitate to ask. You can contact me by email.
MF4: Ok, thanks.

Follow up:
No follow up.

Interview with MF5

Homepage:

I: Ok let's start at the Pubmed homepage. So go to www.pubmed.com.

MF5: Ok.

I: Ok, so do you know why we are doing this project is?

MF5: Well, I think it is because you are trying to understand how helpful Pubmed can be to us.

I: Well, right, how helpful it can be, but also how and why you use it.

MF5: Right and then you are going to follow up with us in August.

I: Yes, hopefully. So, up until this point, you have had access to online articles because you're taking this course at Centennial. So how have you used the databases? What's been your experience?

MF5: Well, I have been using the databases to find research on massage therapy which is what I do.

I: And what kind of research are you looking for? What's important to you?

MF5: Well, I guess I am looking for credible research- I don't really have a preference for the different types, as long as it is considered credible.

I: And what would you consider credible?

MF5: Well let's see, evidence based research,

I: Ok.

MF5: Standards, evidence, clinical trials, randomized control trials, stuff like that.

I: Ok, so you like the empirical stuff.

MF5: Yah, yah, there's a lot of qualitative research out there on complementary therapies, but not a lot of quantitative so I am looking for quantitative oriented stuff.

I: Ok, I understand good responses.

MF5: Yes, this is all pretty new for me, learning more about the articles and the abstracts, I'm a novice, so if I say something incorrect please let me know.

I: No, no, no, there isn't anything incorrect. So, not to worry. It's all about getting more experience. It'll be less intimidating you need to figure out what's right or wrong for me. (I explain the homepage to her)

MF5: Ok, so that's where I start doing my searches.

I: Yes, right. So just so you know there's lots of places on this page to begin, so that just explains the drop down tab. Ok, so let's start with a basic search… what would you like to start with?

Boolean:

MF5: Umm, something with pregnancy. Or is that too general?

I: Hmm, may be a little too much.

MF5: Ok, how about reflexology?

I: And pregnancy? That might be good.

MF5: Ok, so I would be typing in reflexology with the Boolean operator AND pregnancy?

I: That's right. Very good. So you know how they work, right?

MF5: Pretty much. They are just like ways of pairing things together to create topics.

I: Right, they help you to pair things together to drill down, to limit your searches to be either more specific or more general. So let's say with reflexology OR complementary medicine AND pregnancy- do you see how it broadens it and limits it at the same time?

MF5: Uh huh. And should we be using capital letters when we do this?
I: hmm, it might depend on the database, but I think in Pubmed we should use caps. I am not completely sure though. So we are at reflexology and pregnancy.
MF5: Ok, 1-20 out 196.
I: I got the same…. And the first one is Wickam?
MF5: Yes.
I: Great. Now, will you look through the abstracts and choose one that is good for you? That is interesting to you?
MF5: (Takes some time). Well, they all look pretty interesting to me.
I: Ok, actually, before we continue, I am going to give you a brief overview of what we are looking at. (I explain why things are highlighted in blue – clickable, the title, the name of the journal. Indexing for the database, the number beside the article for selecting an article to send to different places).
MF5: I thought things were underlined because it lets you open up the, uh abstract.
I: Well, you can do that or you can click into the abstract. But before we go there I just want you to notice different thing about the icon.
MF5: Like if it is blank it means there is no abstract …
I: Right, very good, I think you are better at this than me!
MF5: Isn't it amazing – I have learned so much in a small period of time. Isn't it that if there is no writing there is no abstract, if it is green then there is access to the full text of the article?
I: Perfect, ok, well let's just move on then. Ok, so you can go into it by hitting the icon or the author,
MF5: But when we click into it, even if it is electronic, it doesn't mean that we can get access to it, we would have to subscribe to it, is that correct?
I: Absolutely, you would need a subscription. So, how important is it to have access to the full text, or is it good enough to just look through the abstract?
MF5: I usually base it on how the abstract sounds to me. If it sounds like it is something I would be interested in then, yeh, the full text of the article becomes a little more important so I could read at least the methods, and you know what, the whole thing, so I have to say its pretty important.
I: It's important to you to have access if like the abstract but you have enough info from looking at the abstract to know whether you want it or not.
MF5: Correct. I can use the abstract to judge.
I: ok, good, can you read through this abstract and tell me your thoughts about it?
MF5: well it talks here do you see about the p factor? I am not sure what that means... like, .0001, I don't remember what that term means. I'm not really up with statistics per say, I'm not really … I can understand some but not all. But the article, I mean the abstract seems pretty straight forward. I would be interested to see more.
I: Ok, so just go back and let's go over some more features.

Related Articles:
I: Ok, so this feature presents you with more drilled down information, actually, do you know what I mean by that?
MF5: Refining the scope.
I: Exactly, so what this does is take the info and it searches based on those criteria (I explain more). Look through this list and see if the abstracts are actually related to what you are looking for.

MF5: Well, I would be interested in looking at number four. This one talks about Premenstrual symptoms and I am not sure how that relates to pregnancy.
I: So a few would be…
MF5: Yes, I am not sure of number 14, of a technical term here, it says something about pre-assessed something, are you aware of this term?
I: Uh, no sorry..
MF5: Yeh, well, not a lot of these seem to be directly related to pregnancy, some of them but not a lot of them.
I: So overall, would use the feature?
MF5: Oh definitely, to see what it produces. If it produces nothing of interest I'll just go into something else.
I: Ok. So let's go back to number 5 and we'll go to links. Choose books first.

Books
I: So we are at the same abstract, but now there is a number of things highlighted in blue (I explain why… links to books … opportunity to seek out more context…)
MF5: Ok, let's look at lymphatic.
I: (we click on lymphatic and I explain more). Ok, some of these books are actually online… do you see this is like a table of contents. In fact it looks to me like a compilation of journal articles.
MF5: Ok, I think I am little lost here. Where are you?
I: Ok, let's go back a few pages.
MF5: Ok.
I: SO, now click on the book cover you see there.
MF5: Oh, I clicked on the number 147 beside that … ok, now I see.
I: Ok, so this is the TOC, and you may search the book for whatever you wish to search for. Now let's do what you did.
MF5: I am thinking actually that they all go to the same place, at least that is what I am thinking so its good…
I: Yes, its good to make mistakes some times. Ok, so this is giving you the articles in the book relating to lymphatic drainage. (I explain more….).
MF5: what kind of order are these terms in? Is there some sort of ranking system or something? Is it ok to ask that?
I: Hmm, of course- it's good. You obviously get it. I assume that there is some sort of ranking, but I am not sure how exactly it is done. So what do you think of that feature.
MF5: I really like it, if I am writing a thesis for a paper, I would see this as very useful.
I: But, for what you do… do you see yourself using it very often?
MF5: Well, I wouldn't say a great amount but I would use it on occasion. If I was doing in-depth research on something.
I: And how often do you do on depth research?
MF5: Not a lot.
I: Ok.
MF5: Well, when I was in school, I graduated a little while ago, the opportunity to search online wasn't really available. And that it is online… and now that I am an alternative health field it's a lot harder to find that information, I find.

Linkout
I: (I explain the purpose of the tool). All of the information about how to access the article. Here we see the publisher – Elsevier, it is available online with subscription, as well as where it is available in libraries. So here the Ontario Scholars Portal has it.
MF5: hmm, I am not quite familiar with that portal…
I: ok, so here are all the colleges that have the article. Let's see if UofT has it.
MF5: Well, I am close to McMaster….
I: Do you see yourself doing that?
MF5: Well, it is a possibility. I don't particularly like paying for things online, I don't think that that's a safe measure with my credit card online, its something I am particular about, I have done it but I don't like doing it…
I: I see…you don't like to give out your information.

Limits
I: OK, let's go back to the homepage and go over another little feature. Limits- do you see up there in the tool bar…
MF5: Yeh, I did look through the tutorial, I did this a while ago, I didn't actually do a search with it but I did look through it.
I: Oh great, well, its basically another way to limit the scope (I explain all of the possibilities; language, gender, subsets. Abstract/no abstract. Entrez date. Ages, We put on some limits. We search reflexology and pregnancy).
MF5: I guess we shouldn't put too many on.
I: Yes, you might limit it too much
MF5: Oh, this found a number of articles, and there is a free one down there too. Yes this looks pretty good.
I: Ok, so between this and the related articles, which do you prefer?
MF5: Hmm, I think I prefer this one although, although I know what I am looking for here.
I: yes the tools are a little different in that respect.
MF5: I think I would sue this one to look for something very specific but if I wanted to browse around I would use Related Articles.

Send to:
I: Ok, so select an article and all I want you to do is place a little check mark in the box next to the number. Do you know how to send things to your email or save them on your computer?
MF5: I have sent things to my email before.
I: So this might be a little redundant.
MF5: Oh that's ok…
I: (I explain 'send to'). Let's start with email. (I explain all of the options- sending with abstract etc…) Do you know what 'html' is?
MF5: Umm, I guess it would include all of the graphics.
I: Right. (We mail citations to ourselves). You can send the information up to your clipboard and amalgamate and then decide what to do …
MF5: Oh, but how do we then go to our clipboard?
I: Well, let's just try it (we select things and send it to the clipboard).
MF5: Oh there it is up there I just click on it then?
I: I am never going to be able to teach you anything because you keep figuring things out.

Very good!
MF5: Oh, I am sorry…
I: No, no it is ok, I was just kidding. Ok, so let's send these to our email.
MF5: Oh yes that's very good. But I have a question for you, it says here that all of the items that will be stored here will be lost after 8 hours of inactivity. So they will be lost if I don't do something with them?
I: if you have stored them on your clipboard then yes, you will lose them. Do you know why?
MF5: because it ends up taking up too much space on Pub med.
I: Exactly. Ok, hit send to text and see how it looks like a typewriter
I: (explains why this is important). So which one did you like the best? The email or…
MF5: Well, I certainly did like the email the best. Although the clipboard function is great because you can sort though all of your results, so I would say a combination of those two.
I: Ok, great, well we are just about done, do you have any questions?
MF5: Well, I wanted to know about the Mesh database is. Mesh more specific?
I: Well, it is basically a controlled vocabulary. So, it helps you find different ways of naming things or finding different terms for you to help you with your searches. I don't have much experience with it…
MF5: Ok, well I can contact you in the future..
I: Yes, of course. Do you plan on using this in the future?
MF5: Yes, I do… you reaffirmed things that I thought I was doing right... that was good, I m really happy we did that. I do picture myself using it.
I: What do you think you would look up? How would you use it?
MF5: Mostly, complementary and alternative therapy. I always have clients who are asking me about information that has come out on certain treatments so that is primarily what I would tend to use this for. Or if I come across article in magazines, I don't always trust what the author has written you know there may be a bias or an opinion and I would prefer to research the actual thing.
I: Oh great, really interesting. Would use be able to help someone else in it? Recommend it to others.
MF5: Oh definitely.
I: So what did you think the most valuable feature was?
MF5: I think going into the limits was, I really wanted to learn how to narrow my search, I think that is more beneficial than related articles.
I: So, how will you do your searches in the future.
MF5: I think I will go straight to limits now. Before, it was the Boolean operators, but it was too much information and I was worried I was going to get lost. But I think it will be easier with the limits.
I: Ok, that is great information. Thank you.
MF5: Thank you- that was great for me too.

Follow up:
None.

Interview with MM6

Background and homepage:
MM6: We need to be able to search, well, Pubmed, and there is also an Ostmed: and Osteopothy database. Out of Texas University. One of them in the south there anyway. I have been there and have searched in there a little bit.
I: How did you do?
MM6: I did ok, looking for lower back resources/ articles and I did find a few. It is actually a web site, or a search for articles that has been discontinued. In other words, they have stopped adding to it they are only maintaining it.

Computer use:
(MM6 describes himself as an "average at best" computer user.)
I: How often do you use it and what do you do, mainly?
MM6: I use every other day, email, communicating within the class for Osteopathy- we might send on an article. Little bit of searching. Year-end papers for the program. A few web sites.

Used the web to find out more about a personal medical issue:
MM6: My daughter had nephrotic syndrome, so I just looked that up last week.
I: Is she ok?
MM6: She is actually, she basically healed herself.
I: How did that happen?
MM6: She had had a severe stomach illness with diarrhea, and after that she had become swollen. Really swollen. And we took her to the doctor because it wasn't going away, and he basically diagnosed this through the blood urine test. Anyway, he wasn't a real talkative guy so we searched it and found lots of material so, we read about that. We also had a Chinese medical doctor working in our office. She was kidney specialist, so she was pretty helpful. Actually much more optimistic about how it was going to go. She said 90% of kids heal themselves and sometimes they'll use a Chinese herb to help the process, whereas he (the doctor), was kinda leaning towards doing some meds, but they were pretty severe. So anyways her swelling went down over the course of the week, so I'll look things up as need be but I do not spend hours on the computer by any means. There were some articles, and I looked in Ostmed, and they would just give the abstract. One of the instructors said many times you can go to the library and get it for free. So I went to the library and the librarian told me that they can't get any articles from an American university or through the states. It has to be in Canada. So I had to contact them and pay for the article.

Regarding his thesis for the Canadian College of Osteopathy:
MM6: Well we can do a little study. But of course we still have to back this up with the research that's out there on our subject.

Homepage:
I: Have you ever heard of Pubmed before?
MM6: Yes.
I: It's a life sciences database with full text articles in it. Sometimes these articles are a little difficult to read and specific. So we don't only use this database to find information.
MM6: Oh, ok.
I: Journals is another database. 'Books' is another database, of info about books, like textbooks. Genome structure… all of these are very specialized databases. You can access the databases when you scroll mouse over the white, and you can click into the different databases.
MM6: Ok.

I: Most web sites provide more than one point of access to a database. And so you can get to any of these databases by clicking on the drop down menu where it says search Pubmed.
MM6: Oh yeah, ok, and there is many more actually.
I: Right! So the ones across the black bar are probably the oldest and the most used divisions, and the ones that drop down get even more highly specialized. Ok?
MM6: Right.
I: Now down the blue bar are some more tools and more databases. You have access to the journals database, if you want just click into it quickly.
MM6: Umm, down on the left side?
I: 'Yes, do you see where it says …'
MM6: Yup, ok.
I: You see it has information about all of the journals. And then there is the MeSH database, if you click on that."
CM16: Yup, ok.
I: And the MeSH database is a controlled vocabulary database. And this is not actually a database with information about articles. It is information about how to search Pubmed. So that if you were looking for something in Pubmed but don't know how Pubmed would refer to it…
MM6: Yah.
I: Then this is where you would look up your search terms. We can try it at the end and practice it. It's a very useful thing but it's a bit complicated. Must know that you can locate it through this blue bar so if you ever need it you know where to find it.
MM6: Alright.
I: Then there is single citation matcher. So if you already know about an article, and you want to find that article this is where you would do it. And you don't have to fill in ALL the fields. just give it enough information so that the database can call the article. So, my point is, with all this, that there is always several ways to get to one place.
MM6: Right, ok, perfect. Looks good.
I: Now hit Pubmed on the black bar, and we'll get back to where we started. Just a little bit more of an orientation. There's the tabs that look like folder tabs.
MM6: Right: limits, preview, etc…
I: Right, so just know where those are. And what we'll do first is, you are into osteopathy?
MM6: yes.
I: Ok, that'll be our first search. Type osteopathy into the field, and then hit go.
MM6: Alright. We've got a few. 258,000.
I: Yes, and that's not a good thing since you don't have the time to go over this many articles.
MM6: Right.
I: Ok, so the first tool that we are going to do is Boolean searches. And actually, Boolean is a search technique that uses language as a way to limit the scope of your subject. And it is actually not even a Pubmed feature. Its something you can apply to all databases.
MM6: Oh, really?
I: And there are three Boolean techniques: AND, OR or NOT. So if you put osteopathy AND joints, make sure AND is in caps, you are essentially limiting the scope of your subject matter because you are essentially making it more specific.
MM6: Yup.
I: If you did osteopathy AND joint NOT knee, you would get any article that mentions joints, but no article with joints and knee- so you are limiting further.
MM6: Gotcha, right.
I: If you hit osteopathy OR joints, I don't know why, you would be widening the scope of

your search. Got it?
MM6: Yes, yes …
I: And that you can even apply to Google, by the way. So if you were trying to find some literature by just going to Google, and you were having trouble finding stuff, you can use the Boolean technique to search.
MM6: Ok, that's good.
I: So, let's hit osteopathy AND joints and then hit go.
MM6: Ok, yeh, well, its 15, 000.
I: That's it? Ok, well, let's stick with this 15,000. We are not going to go through ALL of them, but I just want to orient you within this search screen. Basically you have at the top something, you see where it says 'display': summary, Show: 20, sort and send to?
MM6: Yes.
I: Ok these are all options about how you can see this list. So, it could be that you only want to see the abstract or the brief, so you can play around with this. I keep it on summary because I am not an expert in Pubmed, but once you play with it more, you can change around not the search results, but how you see the search results. You can present the information to yourself in a more helpful way. I think this is pretty good though…
MM6: Sure.
I: You can show up to 500 at one time, or you can show only 5, in case you don't want to look at all of this information at once. You can also sort it alphabetically by author, journal the most recent or the earliest. So then we have our citation. Do you know what a citation is?
MM6: Well, you better explain it to me. I'm not exactly sure…
I: Ok, no problem. A citation is just the name of the author, the journal, the abstract. It's what we call that group of elements. So number one is a citation. Does that make sense to you?
MM6: Ok, yup.
I: So, all of that information is called the citation. Next to the citation is an icon of a piece of paper. Do you see that?
MM6: Yes, I do.
I: And do you see how number three does not have any lines on it?
MM6: Yes.
I: Ok, what do you think that means?
MM6: umm, no lines on it. There might not have written a longer, there might not be any wording on it?
I: Kind of ... it means there is no abstract associated with it. An abstract is a common part of a citation. It often has an abstract, but doesn't necessarily have one. It does have to contain an author's name, and the name of a journal, but it doesn't have to contain an abstract.
MM6: Right.
I: Most journal articles do have abstracts associated with them. An abstract is a little summary of what is contained within the journal article. It is a summary of the main points and findings. So, basically, number 3 does not have a summary, but number 2 does.
MM6: Right.
I: And that little paper icon identifies that.
MM6: Gotcha, so we've got an abstract when there are lines on it.
I: Exactly.
MM6: On one of the articles I had ordered, we had an abstract there, and I thought, it was more to test out this whole system because the abstract was helpful but I thought I would get the whole article. So on a few of them they sent me the full article, but on that

one they still charged me, but it was only the abstract anyway. So I didn't need to order the article because all there way the abstract… but it didn't say that.
I: Oh, that doesn't sound fair…
MM6: It was a rip off…
I: So you paid for the abstract?
MM6: Yes, well exactly. Because I said I want the articles on these, it was four articles, they sent them out, but the one of them was still just the abstract.
I: You should get your money back.
MM6: Well your right.
I: I am serious! Because an abstract is essentially, almost like advertising for a paper! You shouldn't pay for advertising…
MM6: Yah, exactly, well I thought this is crazy I mean I have the abstract right there. Ok, well that's just a sideline, ok, let's continues.
I: No, no, that's important. I actually want to hear more of your voice than mine. I tend to talk a lot so you can cut me off any time you want…
MM6: Alright.
I: So I want you to notice how on the citations, each one has a blue name that is a link essentially. So let's click into the first one, and I'll show you what a full citation looks like.
MM6: Alright, ok, now this is the authors with the blue underlines.
I: Right, and the reason why their names are still underlined in blue (after we have clicked into a full view of the citation), is that you can click into that link and find more articles by the same authors.
MM6: Oh I see.
I: Alright, you then have 'Chang. Gun. Med. Journal', which is the name of the journal. Then you have 2005, 28, 6, 411-20. Those are the year the issue and the pages, then you have the title. Then you have some info about who they are and where they are from, and then you arrive at the abstract.
MM6: So this is the summary of their study ... ok, got it.
I: Exactly. Ok, so now just go back, and I'll show you a couple of other things…
MM6: Wait, 'related articles', to the right there, that's related articles that these authors have done?
I: That's a very good question- we are going to go over related articles soon enough- so that's great. But in short, what that function is is articles related to this article. Not necessarily to the author. The way to get more articles written by the author is by clicking on the author's name which is underlined. But the way to get more articles like this one is by clicking on this button. Soon enough, when we find an awesome article for your needs, we are going to click the related articles button, and see if it works for you.
MM6: Ok, alright, but for now, you just want me to go back.
I: Yes, there are still a couple of things to go over.
MM6: Ok, it's all good so far.
I: Ok, so now just scroll down, and what we want to find is one of the pieces of paper that has either a green or an orange bar on it. I haven't seen any yet.
MM6: I don't see any yet either. We should probably go to the next page.
I: Oh, I found one, number 37.
MM6: Yup- I got it.
I: Ok – that green bar across the top indicates that this article is free. So click into it …
MM6: Yup, xxxxovitis inflammation of the tendon…
(we find a Spanish article)
I: I will show you a way to limit the languages so that doesn't have to happen.
MM6: Ok. How about we look at 52? It says free full text. And this one is in English.
I: Ok, I don't see an abstract… did you click into it?
MM6: I did.

I: OK, so I want you to notice that the way you end up accessing the free full text article is by going to the journal's web site. So the Journal of Rheumatology essentially, has a web site where they publish their research. So note that the article is not located within this database, you have to go t another web site to get it.
MM6: Right.
I: So if you just hit journal of Rheumatology, and we'll go that web site.
MM6: Got it.
I: Ok, and here we have our authors, and umm….
MM6: Ok, good.
I: Ok, I still don't see an abstract for this article…
MM6: Yah and you can use the abstract to decide if the article is worthwhile.
I: Right! Now if you go back to the Pubmed window, I would like to point something out.
MM6: Ok, I'll get rid of this…
I: Ok, notice that it says 'publication type: editorial'.
MM6: Yup- I see that.
I: Ok so basically an editorial is an opinion piece of certain issues. It is generally not a research study. So one of the reasons why this one might not have an abstract is because it is not a research study, and therefore there is no 'findings' to summarize. It is almost like a letter.
MM6: Ahh, gotcha
I: Alrighty then, let's go back and find something else. Hit next.
MM6: 67 has one… looks good.
I: Ok, look at the abstract, and let me know whether the article looks interesting, and whether the abstract itself is helpful to you in determining, in being able to evaluate the article… and take your time.
MM6: Ok (Time passes). Yes, well it encapsulates…. I mean they laid out what happens. Umm, surgical intervention, and then post-op. So, it's an abstract and I think it's fairly clear.
I: Are you interested in reading more about it?
MM6: Umm, I mean yeah, yeah, this is an area of interest. They are talking about ligaments in the lower back and umm, Hematoma, I mean this is probably not… I mean yeah it says right here- rare case. Because I was going to say this isn't something I ever run across.
I: Yes.
MM6: So that would be the only part about it that would, you know myself, I wouldn't go into this one because of that.
I: Because it's rare?
MM6: Yah, the rarity of it. And, well would we see this or not? I mean, possibly. I mean the person would have no idea, just have back pain, and you know we would end up looking at this. But I haven't seen one in 16 years.
I: Oh! Ok! Well, for now, let's move on. Put a check mark into the little box near the number 1.
MM6: So click, ok, put a check in number one.
I: And then I want you to hit send to at the top of the page, and hold the mouse down and scroll down to clipboard.
MM6: Ok, sorry, tell me where you want me to go again? I clicked on number 1.
I: Ok, and go up to send to, do you see that at the top there? Send to…
MM6: Ok that I am having trouble with. Its at the top, in the blue?
I: Nope, it says display…
MM6: Oh yes, I see it gotcha, ok sorry.
I: Its ok… just hit on the arrow downwards and
MM6: Ok, go to clipboard.

I: Ok, now you'll see it says in pink (I read the instructions).
MM6: Right, ok, so you kind of archived it.
I: Right, well what you did was you saved it, well, Pubmed is like your workspace and you saved it into your workspace so that you can look at it a little later. So at the end of the hour, we can go and delete if we find it useless. But, we'll just keep it there for now, and we'll go back and evaluate what types of papers we have there and which ones you might want to email to yourself later on at the end of the hour. And note, that these articles don't stay here forever, they get deleted after a while.
MM6: Right, 8 hours …
I: Right, 8 hours of inactivity. So, technically you can keep them there forever, you just have to make sure its never not active for 8 hours."
MM6: Right (we laugh at that).
I: Ok, let's do another search because it seems like we haven't really found such good free articles. So, what else can we look for? What else are you interested in? What did you think of the article I sent you?
MM6: It was somewhat interesting. But it was, umm, well, I mean I could skip to the conclusion and sort of gone, oh yeah, that is what we tend to see. Certain number of back pain patients. It seemed like it was almost confirming what we see all the time anyway. Anything spinal and lower back is good. Relationships between visceral organs and spinal dysfunction.
I: Oh! That sounds really medical… I love it! Let's look for that. What' is it again?
MM6: Yeah, viscera and spine.
I: So, how do you spell viscera?
MM6: Ok, it's viscera.
I: Ok, we'll do viscera AND spinal…
MM6: Well, we could do spinal dysfunction, spinal pain, may be spinal pain would be more common.
I: Ok, let's do it.
MM6: So your putting in Spinal AND pain AND viscera?
I: No, I am putting in Viscera AND Spinal pain. I am not doing AND pain, but we could check out if there is a difference afterwards. And then hit go.
MM6: And when you did spinal pain did you do spinal AND pain? (Probably did not hear my explanation as to why above because was concentrating on what he was doing."
I: No, I just did spinal pain.
MM6: Oh, ok, it'll pick up on both… like its two separate words. Spinal pain.
I: Ok, hit go.
MM6: Ok, we'll see if we got the same thing. 128, is that what you got?
I: Yes!
MM6: Ok, good.
I: Ok, again, will you scroll through the list and see if any of these articles look relevant for you. Take your time… see if you'd want to read any of them. Look at the abstracts if you'd like. We aren't in a rush.
MM6: Well here is one, spinal manipulation for primary and secondary dymennerea.
I: Uhh, what number?
MM6: 6. I'm just going in the abstract. Ok, so they have a good size abstract in here. It looks like an interesting one.
I: Ok, does it look like you want to read it?
MM6: Yeah.
I: Ok, great so this is the one we'll use. From here, we'll go to related articles.
MM6: Ok
I: Ok, so click on related articles.
MM6: So now we have 278… (referring to the number of articles).

I: Right, so we have 278 articles that relate to the initial article. Which means, may be we have a list of articles that is useful to you! So have a look through them and tell me whether indeed they are related to your interests. Tell me whether these are even better than the last ones.
MM6: Ok, well, the first two are the same ones. Ummm, ok they are talking about acupuncture with it. Ok, so now they are going through the pharmacology to treat it. (reads to himself a little more).
I: Oh!
MM6: Umm, so yeh, now I think its … well the first few we had the same articles, then there was one on acupuncture and now a lot of it is getting into more, uh, pharmacology, surgery, we even have one further down that talks about ankle sprains.
I: So do you think that these articles are really related to the first one?
MM6: Not so much- just the one really, near the top. Yah, the first two were the same, then we had an herbal dietary thing, then we had the acupuncture, and then from there on below four, it, yah, not as much.
I: Ok, interesting stuff. Well, if you ever find an article that is exactly what you want, and you want more of those, you might want to try related articles. But it doesn't look like we've had very much luck. But, do you understand what the tool does?
MM6: Oh, yes. I do, and I mean certainly, I mean it is capturing, I mean related, it makes sense… it captured half of it in all of these things. I mean endometriosis – same sort of area. So yeah, I get it.
I: And do you think that you would use it if you ever came back here?
MM6: Related articles? Oh yes…
I: Ok, great. So let's go back to our list of 128, our search that we found on viscera and spinal pain.
MM6: Ok, so go back to the list. Ok, 128.
I: Ok, and have a look at number 128.
MM6: Oh, yeah right because its got the whole article.
I: Exactly. Now does it have the whole article, or do you have access for free to the article?
MM6: I guess I have access for free to the article?
I: Very good. I am sorry to be so picky about that.
MM6: No, no, it's good.

Pubmed Central:
I: Just want to make sure you know that the article is not in the database. Because there is actually another indication, when there is a green and orange bar it means that you are actually finding the article with full text within Pubmed Central. Which means that you still are in the Pubmed environment. I want to show you what that looks like because then it gives you access to all of the tools that Pubmed has.
MM6: When its just the green, then what'll likely happen is the same as the last one, it'll show us the web site it will go to get this article.
I: Yeh, it'll open up another browser for a web site, and in this- oh I found one- go to number 54 to go into Pubmed Central.
MM6: Ok, you said 54?
I: Yeh, 54. Ok, so do you see what happened here?
MM6: Well, we clicked into … oh I see. At the top it tells you Freefull text article in Pubmed central.
I: Right, so you have two options here. You've got free full text article at 'www'. And you've got the article free in Pubmed Central . So you have two options here: one is to go to the journal web site, and one is to stay within Pubmed central. I don't know if one is better than the other necessarily, but that's what the difference is. If we were talking a

few years ago, I might say that staying in Pubmed would be a benefit because of all of the functionality that the web site has, in terms of related articles, etc… but now, most journal web sites have that functionality. So I'm not sure if there is a major difference. But let's see: click on the Pubmed Central icon.
MM6: Alright.
I: So when you have an article free in Pubmed central, you still have access to what I call functionality: figures and tables, related material, Pubmed related articles. Do you see that?
MM6: Gotcha and its got full text highlighted…
I: You have access to records, taxonomy tree.. so when you become a Pubmed expert you will be able to use all of these tools. It's one of the things that comes with staying in Pubmed. Does that make sense to you?
MM6: Umm yes.
I: Ok, let's close out of this for now, and click on the 'free full text article' button.
MM6: Yup- so I'm there.
I: ok, you've got the article, you've got services and functionality- it links you up to Pubmed and to something else called Web of Science. And remember this is not a database but a journal web site. Ok, you can even go to the current issue of PNAS (the name of the journal), and browse through it if you wish to.
MM6: I see, ok, that's good.
I: And then you can subscribe.
MM6: Of course.
I: Ok, so close out of this window, now actually, you might wonder why this particular article is free, while other articles in the MM6ournal are not- you have to subscribe to it. It could be that they have a policy by which they release their articles six months after they have been published, a delayed release, or may be they just decided to release this one free for some reason- who knows why…
MM6: Yes, right obviously, I am just scrolling around, very few have the full text articles.
I: It really depends on the topic. Some have a lot and some don't.
MM6: So, if I want to see some of these other articles that only has the abstract, then I am going to have to umm, contact that university or journal or what have you.
I: Yes, exactly, and I'll show you how to do that more efficiently. But first, will you try to send yourself this article?
MM6: Sure, I can do that.
I: Ok, what do you do first?
MM6: Ok, I have to click on number 1 and then send to clipboard.
I: Yes, but its number 54 …
MM6: But I am in the article…
I: Close out of the article first, go back to the search list and
MM6: Oh, ok, I see now. Number 54, got it.
I: Great. It's easy to get lost, eh?
MM6: Yes, and the problem that I've got is that we have no drapes on our window and I am fighting the sun to see the computer screen.
I: Why don't you take a minute to fix it? You shouldn't be uncomfortable while we do our interview. Take your time.
MM6: Ok, so I just put 54 on the clipboard. So my question is, does it show anywhere that I have got two articles on the clipboard?
I: I am glad you asked that, because I can answer it. All you have to do is click on clipboard up at the top, where the tabs are and…
MM6: Ok, gotcha…
I: And it shows you both of your articles…
MM6: There you go. They are in there.

Linkout:

I: go back twice now, to our original search. Now, choose an article that you are interested in, but it is not free. We can look at whatever you want.

MM6: I am in 40, how to I get out of it? (referring to the page number)?

I: If you look down at the bottom, you can see that there are page numbers below, and you can put in any page number you want (into the little field.)

MM6: Ok, so I am back to one now. Sure number 1 looks good.

I: Ok, hit links and then choose link out. See another screen pops up.

MM6: Yes, ok.

I: Can you tell what this is?

MM6: Well, we've got another article I see…

I: Well, kind of... let me explain. Basically what link out does is provide you with a link to get to the article. So basically, if its not a free article, you need to know where to get it- there won't be a free access link to it. So you can do a bunch of things: order it, download etc…but this shows you who the publisher is. So if you know that say UBC has a subscription to EBSCO, then you know that they have this article. Is that clear? So Blackwell, Casper, Ingenta, Ebsco and Ovid are the publishers or aggregators of this journal. It comes in PDF. You can get it at several libraries. Click on libraries, and look at the alphabet. Click on U and we'll see if UBC is there.

MM6: Doesn't look like it.

I: Yes –it's there.

MM6: Oh, I see it now. Electronic full text.

I: Ok, so click on electronic full text. And it takes you to the journal and the abstract. If you wanted the article you would have to log in. If you were a UBC student, you might have that info to put in at home. If you were at UBC, physically, you might be able to access it and print, and pay for the pages it is printed on. So that is what link out does. First of all it gives you the aggregator and publisher, and then you can order it through doc. Delivery, and it can go straight into your inbox, or you can go up to UBC and get it.

MM6: Right.

I: Is that clear?

MM6: Yes. I will use it because odds are the article I am interested in, I am going to have to go and get it. Umm, so I would have to use it.

I: How would you know if you wanted and article?

MM6: Umm, well I mean if its in what I am writing about, the title is going to give away some of it. Now it may turn out that ... oh actually, I know- the abstract. The abstract will give me a fair idea of whether it is useful.

I: So an abstract is important to you?

MM6: Yes.

I: Sorry- I have to ask these types of questions.

MM6: I understand- you are building data.

I: Ok, so that's Linkout. Should we try it again with another article?

MM6: We had better.

I: Ok, let's try it with number 2.

MM6: Ok.

I: Ok, you see it says full text article?

MM6: Umm, beside number two? Oh sorry, I clicked number 2 and put a check mark in the square and now I can't get rid of it.

I: Ok, just click back into the square, and you will be able to get rid of it.

MM6: Ok, great. So go into this article. Ok, I am in the article.

I: Ok, do you see Elsevier full text article button?

MM6: Ok, yes…

I: it also takes you to article's abstract. Now this is another case of needing a login and password to get to the article. So that's not going to help you. Now X out of it if you can. I just wanted to show you how it would be done if you had access- it is one point of access. But you are not a member so you cannot get it. Ok so now go to link out and read it out to me since mine isn't working…ok, so what do you see?
J: Ok, well I have the article and then Elsevier Science is the journal, I guess..
I: Is the aggregator. It's not the journal.
MM6: Oh, ok.
I: the journal is pharmacology and biochemistry behavior.
MM6: Uhh, right, where is that?
I: that would be with the abstract. Does that make sense?
MM6: yup, ok, so now umm, and then I go into libraries, because from this point if I want to see that abstract again., do I get there through libraries? No..
I: if you want to see the abstract again you don't need access to the journal, you need to go back to the search list. I was just trying to show you that if you wanted the article, you would go to Linkout and checkout who the aggregator and the publisher are, and see if UBC is a member …
MM6: ok, so I am going in there right now. And then I have to go to U- for UBC …
I: Yes, or go to Simon Fraser, may be.
MM6: Oh, it might not be there this time. It doesn't look like it. I'll check for Simon Fraser.
I: Ok, if you really wanted this article, and UBC isn't on the list you can get it through infotrieve. Do you see that link there if you click out and go back a few times?
MM6: Go back to the list?
I: Ok, go back into links and go to Linkout.
MM6: Ok, were we in number 3? I can't remember.
I: It was in 2, but it doesn't really matter, I am just showing you as an example. I just wanted to show you how if you did want an article, and couldn't go to say UBC, then you could try something called Infotrieve. Do you see Infotrieve there?
MM6: Uhh, not yet…
I: Infotrieve- as in retrieve.
MM6: Uhh, is that over on the other side?
I: It's under Linkout.
MM6: (Having trouble finding it). I wonder if we are in the same spot…
I: Ok, you know what? We are going to skip that anyways. It's a little expensive –
MM6: Oh really. Does it? Ok.
I: So, let's go back to our list, and now, let's work with number 1, the first citation "basic and clinical aspects of visceral sensation.
MM6: Yup, got it.

Bookshelf:
I: Ok, I want you to go to links and then to books.
MM6: ok, yup, got them.
I: Ok, what I want to show you is… do you see how many of the words in the abstract are underlined in blue? Ok, each underlined term, linked term, is basically in a book somewhere. Most of them are textbooks. If you click on the first one, pain,
MM6: Ok, right it brings me up a list of umm, textbooks. Cancer medicine. Right?
I: Yes. So, in essence, the word pain has been 4444 times in HSTAT in that first book. Ok?
MM6: Yup.
I: Ok, so I want you to notice how up at the top of the page, where it says search, we are no longer in Pubmed, but we are now in a new database called bookshelf. Do you see that?

MM6: At the very top- bookshelf. Yep!
I: Well, before it used to say search Pubmed, and now it says search books for “PAIN” because we had clicked on PAIN in the abstract, ok? And so pain is mentioned again 3444 in Hstat, 478 items in Cancer, 297 times in Physical Education and Medication Board review… etc… so these are books, not necessarily about pain, but have pain in them. And sometimes, it will be a book about whatever the search term is, it just depends on what the book is. Does that make sense?
MM6: Yes.
I: So let’s go back to the first citation that we were working with when we clicked on Pain. (Still in the Bookshelf database).
MM6: OK.
I: Ok, just take note that any word that is underlined in this abstract is connected to a book. If you want, you can click into one of them and check it out.
MM6: Yes, got it.
I: Do you think this is a useful tool?
MM6: Umm, I don’t know. It doesn’t really tell me- well, ok, the word pain is in all these books- but the word pain, I’ve probably got 30 medical books in front of me and I’m sure the word pain is mentioned there many times. But I don’t know how that would link very well, with narrowing the search down for me.
I: Ok, good to know. Ok, well, let me tell you a little bit more without convincing you. Let’s say you were looking up massage ‘therapy and lower back’ and you went into ‘books’ to check out if there was an entire book on the subject.
MM6: Yes, ok true enough. That could work. If you had a more general heading, as opposed to these small words that could be, well there would probably be a few then may be the book itself, I don’t know if you click onto them book itself, does it tell you anything more about the book (referring to the picture of the cover page that you can click on to find out more about it).
I: Good question, we were just going to go there. Let’s see what that does.
(We click on it).
I: Ok, I think this book is associated with the National Library of Medicine. And you have here I think the table of contents with what is in the book. So you can get some pretty specific information about what is in the book form this tool. If you are looking for a specialized book, and you hit the books database to see if there is any more specialized information, you can find some good info.
MM6: Yes, I can see how this would be useful. And then obviously we would have to buy them…
I: Yah or you could take it out of the library. Again…I don’t want to persuade you of anything. I just want to present a scenario that is useful to you. I am not giving you a fair shot when choosing pain. It’s not the best example.
MM6: No it isn’t but you broadened my thoughts on it.
I: Ok, so now I want to do limits. Located at the top in the tab.
MM6: Ok, I clicked back to our list.
I: Yah, good, exactly.
MM6: So, we are looking for limits…
I: Can you actually tell me once again what our topic was?
MM6: Sure, it was viscera and spinal pain.
I: Ok, great. So working again with the first abstract. Click into Limits.
Basically this is a tool that allows you to narrow the scope of your search, but not by searching the contents of the article, but rather by searching the information about the article. So, for instance we had trouble at the beginning, because we found a free full text article, but it was in Spanish. Here is where we would go to limit by languages. Do you see where it says that within the limits screen?

MM6: I see something called “All Fields’.
I: Close all fields, and just look at the screen first to see what the info is on the screen.
MM6: I must be losing it because I am not even in all fields anymore. (lost).
I: Ok, click back please
MM6: ok, I am back to the list.
I Ok, click on limits again. We’ll just try it again.
MM6: Ok, so yah, I’ve got limits.
I: ok, and it says limited to: and then you see all fields, publication types, ages, entrez date. You don’t see those fields?
MM6: Well, I guess, its got limited to “all fields” at the bottom.
I: Did you put on a limit may be?
MM6: umm I don’t think so… I went to the ‘limits’ tab, I see ‘all fields’… I click on that but I don’t see languages. It’s got ‘All fields, experiment types, filters….’
I: Hold on a sec… umm, because what I see is, it says limited to, and it says limited to, then it says publication types, ages, Entrez date, pub date, then if you move over, it says languages, humans or animals, subsets and then gender. So have you clicked into ALL fields?
MM6: Yah.
I: Ok, don’t click into it for a sec. Just have a look at the web page before you do that, if you don’t mind.
MM6: OK, I am still there. I am in limits and then its got the Boolean operators, AND OR NOT, more help on using limits available here (i.e. reading the screen). And then limited to ‘all fields’. And that’s it.
I: Hmm, that’s very strange!
MM6: And it’s not at the blue at the side?
I: No, definitely not. Can you hit reload on your browser window?
MM6: Ok, now you’ve lost me… reload on the browser window? (Doesn’t know what that means).
I: It means to refresh your page. Have ever done that before?
MM6: No.
I: Oh, ok, up at the top, what browser are you using?
MM6: IE.
I: Ok, you have the ability at the top of the browser to refresh the page. …

(Here we stop because he was having some problems with his computer. What follows is a second session a few days later.)

I: Ok, can you hear me? Ok, so what is the search?
MM6: Real general one. Low back pain.
I: hit go?
MM6: yah, well I did ‘Low and back and pain.’ And I got 12, 292.
I: Great, and what’s the next step.
MM6: Well, I just went into the first one, which didn’t have it, so its just going to be an abstract without the full article. Umm, so I went in, had a look at the abstract, then went into Linkout, and it’s not in the university list, so I couldn’t remember if we did this. So, if it’s not at the library, in the list here, is there any other way for me to get it?
I: Umm, some times if it’s available electronically, then you can get it. But it looks like its not. If it’s a PDF and it sits on another web site, then usually if it is you’ll see a little sign that says EBSCO, or EBSCO full PDF access, and you would be able to click on that and be able to order it right to your computer. But unfortunately, for some reason, this one does not have that. Let me just look at something. I want to see if it’s a print journal. Can you do me a favour and just tell me what the date is on it?

MM6: Ok, I am just clicking in. You're at the first one?
I: Yah- Fleurmonde?
MM6: Yes, that's it. Uhh, the date, summer 2005.
I: Its really weird. It looks like everything here has it only in print. I am looking over this list (in Linkout) and it says print collection. Let me see I am going to go into University of Toronto and see if I can email it to you.
MM6: Ok.
I: this means, I am going to log into my UofT web site, and umm, look it up in their resources. What I do is, I look up the name of the journal. What's the name of the journal.
MM6: Umm, ok, I guess it is the first part- oh I got it, Ethnicity And Disease.
I: ok, great. This is just an interesting little side bar. I have never done this with anyone! (We chuckle).
I: Ok, I can do this for you, but It'll take a few minutes. Should we bother?
MM6: hmm, I don't know. It'll just be seeing if its there? That doesn't help me anyway…
I: No, if its online it will, I will forward it to you… as sort of a favour.
MM6: Oh, yah, yah, yah. You could get it for me. But umm, but I won't be able to do this all the time, say get me this article…
I: Well if I was in a good mood may be. So what should we do? Forget it, or? How about this, I'll do it when we get off the phone. Ok, when you went to link out to look for it, everything was in print, so it was difficult to get the article.
MM6: Right so even though it just shows on my page as an abstract, I should check link out because I may be able to get it.
I: I think we should click through and find an example of a linkout where there is an electronic article right there. Umm, that's what I would like you to see right now.
MM6: Ok, if you want to do the even ones I can do the odds.
I: Ok, that sounds great. … oh, sorry, but I found one already. Go to number three…
MM6: Ok, so got to Linkout- oh but is it right there? Ok., so I know right away when I pull it up that I can get it.
I: Oh right, I didn't see that. You're right. You'll see right away if you can get it. But this one you would have to pay for.
MM6: Oh, I'd have to pay for it.
I: Yah, in fact, why don't you click on it and see what happens.
MM6: I did go into it. Ok, so go back into it. Umm, so well, ok there is the abstract, and the other part is in German.
I: Right, so when you click on it it asks for your username and password. And do you see on the side? The login?
MM6: So you know right away that you need an account with them.
I: Yup. Most American ones will have Ebsco or Ingenta button. This one is a different button because it is a German article. Ebsco and Ingenta are giants in the publishing industry.
MM6: And they may or may not be charging for it.
I: No, most likely they will be charging for it!
MM6: Oh.
I: Unless you see the green or the orange bar on the abstract icon, they will be charging for it.
MM6: Yes, ok, so basically if I need to get an article here, well, I mean I can always click in and see if I can get it at the university. But UBC, I have never seen SFU, and UBC, only comes up sometimes so it seems like most of the articles I would not be able to see. There is not a lot of green and orange bars around.
I: But what you might be able to do is go to UBC and talk to a librarian, and see what she could do for you.

I: Ok, one more thing. Click on number 16.
MM6: Ok.
I: Ok see that little Ingenta Select?
MM6: Yah.
I: That's the giant.
MM6: Ok, that's the giant.
I: Ok, so a lot of the articles are owned by them.
MM6: So, a person could set up an account with them.
I: With EBSCO and Ingenta?
MM6: Yes...
I: Yes, a person could.
MM6: Do you know how much that would cost?
I: No, sorry. But may be you can go to their web site and look it up. That's a good question.
MM6: Ok, well I mean it is worth it if I see an article that I want, I should check it out anyways. You never know …
I: Yah, you should check it out. Ok, ready to proceed? Check off the little box next to 17.
MM6: Ok.
I: Ok, and we are going to do the Send to function. Go to the top of the page.
MM6: Ok, I see it.
I: Ok, scroll down, and hit send to email. Ok, and then it brings you to another screen.
MM6: uh-huh.
I: And then you put your email address in there.
MM6: Ok, and umm, ok, down right..
I: And if you want you can put additional text- something that describes the article.
MM6: Right. Ok, what was that article?
I: Ok and then your going to hit, mail.
MM6: Yup.
I: Ok, now you have the source in your email in box, and you don't have to worry about finding it again.
MM6: Right, so you can use clipboard or that.
I: Did I show you clipboard the last time?
MM6: Yes, I am just trying to remember how to use it. We click on it for sure, and then we just go to clipboard and this adds to it?
I: No, what it is, is you click off a number of articles that you want. Then you go to your send to button, send it to your clipboard, and then you can look at what you have.
MM6: Oh, yah, yah.
I: First you select the articles, then you send to, and then you look at your clipboard.
MM6: Ok, now I remember the send to button. You can send it to your clipboard or your email. But your clipboard doesn't last that long.
I: Right- inactive for 8 hours, and everything goes away.
I: Ok, let's go over Limits, which is what we were having trouble with the last time.
MM6: Ok.
I: So, click on Limits. Do we see the same thing? Because that was the problem.
MM6: Yes, this time I have got it.
I: Ok, great.
MM6: I don't know what happened the last time. When I tried it again later it was fine. (Describes what he sees).
I: Ok, so basically what you want to do is, you want to think about what type of search you want to conduct. Because we learned how to do a Boolean. And when you do a Boolean you are basically limiting your search or controlling the scope of your search using words or the content. Like, if you are looking for back pain, with Boolean you think

about how to get more specific in order to limit the search: so, you say what kind of back pain? Low back pain, and then you ask in relation to what? And it is in relation to pain. So you have Low AND back AND pain … (tape ends. We have a conversation about research while I switch the tape. My question was lost but it had something to do with access to the research).
MM6: A large part of me wanted to do this because of doing papers for the Osteopathic college is a big motivation. But its also nice to be able to, now that I know a little bit, even if I was not doing the college, to try to stay you know somewhat up to date on the current recent articles on whatever it is. You know PMJ (?), or neck pain or whatever. But of course if people go in and they can only read abstracts and they can never get the papers it is limiting.
I: Exactly.
MM6: Well, hopefully things will change…
I: We'll see. Alright should we talk about limits?
MM6: Limits, sure, ok.
I: So, I was telling you how Boolean changes the content of the search. And so limits changes the stuff around the content. The parameters around the content. Like, what type of field its in, or when it was published or what language it was in, or umm, what type of journal its in, who was in the study. That stuff is meta-data, its not really the stuff of the content of the journal article, but its stuff that every article has. Every article has people with a gender, a language that it's written in… and you can control that without going into the actual theme and content. Does that make sense to you?
MM6: Yup.
I: ok, so we are going to do, low and back and pain and we'll put on some limits. Ok, so first let's choose English.
MM6: Yup.
I: Ok, and humans.
MM6: Yup.
I: Male?
MM6: Sure.
I: Ok in subsets, we'll put it to core clinical journals, which means we are going to limit from other journals which are kind a outside of the fold.
MM6: ok, dental journals … I see
I: Yes, we don't need any of that stuff. We aren't really interested in back pain associated with any dental problems. We'll put Entrez date a year. And then hit go.
MM6: Ok, I just have to punch back in low back pain.
I: Ok, low and back and pain. We got a lot of hits on that one let's see what happens now.
MM6: right. Ok. Yes, well that did a lot, 49.
I: there you go. Will you look through the list and tell me if it's a good list?
MM6: (after a few minutes). Looks pretty good.
I: great, well that was limits. So, notice how there is a yellow band across the top, and it reminds us of what your limits are. If you put on too many, you go back in and change it. What would happen if we put too many on?
MM6: We'll limit and cut out some you actually want. Like gender for instance, I assume they must have done studies on females as well.
I: right, so if you put on too many limits, you would limit your search and you may not come up with anything. So you would want to take some limits off. So this yellow band reminds you of what your limits are, and you can go I and change them. Click on the tab on limits and see like this is what you have already got and you can change it. If you put this in combination with your search, low back pain, you get something pretty specific. You wouldn't get any German titles for instance. It's a little less daunting.

MM6: Ok right. That's useful.
I: well that's limits and that's about all I had to show you… do you have any questions?
MM6: Hmm, well, let's see, I did want to ask you one. You were talking about access… and that there are groups trying to get access and I know I was talking to a librarian here at the public library and she said in BC they were trying on working on, and the government was pushing this forward, although I haven't heard anything lately on trying to link the public library with the university library so that anybody that has a library card can access SFU and UBC you know obviously they are linked with many of the other universities across north American and around the world so they are trying to make it more you know user friendly or allowing a lot more people to access.
I: Wow, that's great.
MM6: Did you hear anything about that in Ontario?
I: hmmm, I'm not sure exactly, but I know there is alliances behind a lot of the different universities especially between libraries. So that's something worth looking into on my part to find out whether they can share electronic subscriptions with each other. .. So that's really interesting.
MM6: Yah I thought so, but of course it's not in the cusp and you know how fast government works.
I: Well, I'll look into that…great.
MM6: And then in a couple of months I'll check in with you… because I got a course coming up, so after that I'll try to mess around in Pubmed.
I: Sounds good. Thanks for taking the time
MM6: oh, no it was good I appreciate it.
I: Email me if you have any questions.
MM6: Ok, thanks. Bye.
I: Bye.

Interview with MF7
I: (I begin by explaining what we are going to do on Pubmed). Our goal is to drill down as much as possible to the find the least amount of references possible.
MF7: yeah so you don't have to, yeah, uh huh, I get it…
I: Ok, so with that as our goal, you have to recognize that this is not going to actually find us the articles, its only going to find you information about the papers.
MF7: Oh, you mean Pubmed does that?
I: It's an 'Index', it does not contain the actual papers.
MF7: Oh, ok!!
I: Yah, and that is one of the reasons why we are going to do this study. To put it very simply, one of the goals is to open research up. Now, research in the form of papers is locked up. You often have to pay to get access to it.
MF7: oh, like the journals and stuff that they are actually published in.
I: If you go to a university, its not so bad because often times but not always, the university will have a subscription to the journal. In contrast to this is the existence of the Open Access movement. Now that it is so easy to publish information in digital information, there are few reasons why access to scholarly research, particularly if it is subsidized by federal research grants, should be limited.
MF7: That's so true.
I: So we are going to go through Pubmed, and I'll show you how to actually get the free articles, because there are some that are free. Some even release their journals articles later on and make them Open Access like 6 months after they have been published.
MF7: Right…

I: So I am going to show you how to recognize the free from the not so free, and I also want your opinion on whether you think you will use these articles.
MF7: Well with my teaching, part of what with classes b/c it is post grad work to a variety of people, I usually present articles about topics that we have covered in class with manual lymph drainage and decongestive therapies. And it's a real challenge to find more recent stuff. Right now what I have gleaned is usually from other people, and it's probably dating back to 1997. To 2003 in terms of what I have got on file, which is hard copy only. So it would be nice to upgrade to drop a couple and add a few because students always ask where does that research come from. I can name my researchers but it's nice if I can provide these other articles.
I: Ok, well I'll teach you now how to use Pubmed so you can get some relevant material, or at least look into whether there is good stuff for your practice. And I'll show you what the principles of searching Pubmed are. Ok, so we'll start now with the first screen that we are looking at (homepage). Now Pubmed is an Open Access database, which means it is free for everyone in the world to use, as long as they have a computer. And it's published by the National Library of Medicine. Now when you type in Pubmed, it re-directs to another web site.
MF7: Oh yes, that NCBI address?
I: Exactly, it does that automatically.
MF7: Ok.
I: Ok, there's all sorts of tools and tricks that you can use on this web site that'll help you find good information. So, we'll start with something very basic, umm the first is, do you see the black bar at the top.
MF7: [lists what is written on the black bar, all of the databases associated with Pubmed}
I: Basically this one page that you are looking at is kind of an umbrella to multiple databases full of information. And as the information grows they might create more databases. And the reason why its not one big database, why there are these divisions, is because it is probably easier if you separate it according to them or topic. [I then explain what the divisions are]. There is crossover between all of the databases. You can click into all of the databases.
MF7: Ok, by clicking into the bar. I see.
I: Yah, but don't click into it right now. You can do similar things, if you take a look at the blue bar running down the side of the page where there are more options.
MF7: Oh, I see!
I: And then it says 'search Pubmed'.
MF7: Oh yes…
I: and you can drop that down, and again you have access to the main databases and even more.
MF7: Ok.
I: Ok, so the first thing we are going to do is a Boolean search. And this is not even a high tech way of searching. It simply means that you are going to take your subject matter and limit it by being more specific. So choose a subject matter.
MF7: Ok, so I type in 'for lymph systems'.
I: no 'for', just lymph systems.
MF7: the latest research on lymph systems would be nice. You see the field of Lymphology is growing because umm, for years and years till 1999 they didn't give it a whole lot of credence in terms of its relationship to the circulatory system. And in 199 there was some huge research that came out from some heavy weights, that showed that

the lymph system was really, really important. So now we've got people on 4 continents to be lymphologists and be the first on their block to publish stuff. So I want to know what it is they are discovering. I belong to the international society of lymphology and I also receive the journal of lymphology here in North America, but that's it. There's a lot going in Europe and Asia that I know nothing about unless they publish in lymphology or unless they present at the ISL. So that's one aspect of what I am interested in.
I: Well, there is a good way to get the most current research possible, but for now, let's just search lymph system.
MF7: ok, so I just put that in like that? Lymph system?

Boolean:
I: Yes and hit go. Ok, there are 13385 hits. Let's drill down. How could we get information that might be more helpful? Because there are too many hits… what else could we put with this topic?
MF7: Ok, how about umm, anatomy?
I: Ok, lymph systems and anatomy. The AND basically limits the scope of the topic. If you say lymph system, then you will get anything in the database that says lymph system. If you add AND anatomy, then you will be searching from anything that has both of the terms- which will be fewer.
MF7: Ok, so I put AND? Ok… oh yeah, it does lower it.
I: That's called a Boolean technique. You put in AND OR NOT. That uses the logical structure of language to drill down. And its not Pubmed technical tool.
MF7: Ok, right. Well there is still lots of stuff there
I: why don't we try to put a NOT- how about NOT cancer?
MF7: So, I put a NOT?
I: And this is called a search string, it can grow a lot longer. But we still have too many, how about if we say not lymph nodes?
MF7: Let's see, hmmm, what about ONLY lymph systems and nothing else? B/c some of these I see are in response to immunity, I see renal stuff here which I am not interested in.
I: Well, only doesn't really work. It isn't considered a BOOLEAN term. AND< OR, NOT are Boolean terms. But we can say NOT renal.
MF7: Well that eliminated about 1000!
I: Ok, well, I think I've made my point. Try Googling 'Boolean' to learn more about it.
MF7: Google Boolean? You can do that?
I: Yes, so you can find documents and resources on the topic.
MF7: Ok, let me just write down a note on the side here.
I: So now let's go down the list, and I'll show the components of the page. Click onto the second page and click under number 33.
MF7: Ok, there it is.
I: What's different about this one as opposed to the other ones?
MF7: The green and the orange.
I: Exactly. They signify that it is free.
K: Oh, cool!
I: Yes, the green means free, the orange means that it can be found in Pubmed central, another database of free articles associated with Pubmed. Now I want to explain the citation information to you. (I go through the information with her).
MF7: Ok, well I have another question for you: what if we put 'free' into the search criteria at the top? (i.e. as part of the search string).

I: Great question! No, because the way the search works is that it searches the contents of the article. So unless free was talked about in the article, it would not come up. However, there is a way to limit to free articles: and that is if you search the Pubmed Central database. Remember at the top? We I mentioned there was a number of databases that could be searched?
MF7: Ahh, ok! I get it.
I: Except you might not want to do that, as it would be a little limited. There wouldn't be that much related to your subject in that database.
MF7: Oh, ok.
I: (I explain the components of the citation- the icon, etc.). If there's no lines on the little paper icon, then it means there is no abstract.
MF7: So there is no summary?
I: Yes, an abstract is like a summary. And most articles have them, but some don't. So click on the author, the blue…
MF7: Ok.
I: Ok, so can you read over the abstract, and judge for me a couple of things: whether the article could be helpful to you, and whether you would want to read the full article, or whether the abstract provides you with enough information to be useful to you…
MF7; Ok, well its not useful for me, for what I do, but it does tell me some information that I didn't know before (she chuckles). The cell type that they are talking about, the engagement of it.
I: Ok, that's totally fine- I mean we selected it randomly because it was free. Ok, well let me tell you about the format of the citation. By the way, one whole thing: a journal an abstract an author etc… that's called a citation. It consists of all of the information about the article that you are looking at now.
MF7: Ok, citation is author, abstract, and what else?
I: the title, the issue the year…
MF7: Uh huh.
I: so now let's go look at the article. We'll look at the red button (the free full text).
MF7: Ok, I click on it, and I'll enlarge it because it comes up as another window.
I: now basically what happened was another browser window popped open and went this web site, and here is the article. Notice this web site has a lot of the same tools as in the Pubmed environment… (I explain that more). Therefore a lot journal web sites now have the same kind of functionality as Pubmed. So they have developed it the way Pubmed developed their site.
MF7: So the full text would include the full text as well as the article?
I: well, we are talking about the actual article when we refer to Pubmed. Because most articles, even 'not free' articles have abstracts.
MF7: Ok, I thought maybe they cut that out too (in other words she doesn't really understand the purpose the abstract serves as an indexing mechanism). I find it interesting that they have corrections posted (this article has corrections posted). that's very cool.
I: It is cool, that's the benefit of the web, you get to link up the different things, and publish really quickly. In a print form it would be a little different.
MF7: Yes, you would have to wait until your next issue came out.
I: Ok, now close this browser window, out of the full text, and I'll show you the difference between the two buttons.
MF7: Ok, so I just click out of the window with the article.
I: Yup, and click on the free FTA in the Pubmed.

MF7: Ok, and it has the same blurb …
I: So what would be the difference between the two buttons?
MF7: Let me see… first of all, I'm not sure.
I: Well, look into the left hand corner of the browser what do you see?
MF7: I see free in Pubmed central?
I: exactly, so as opposed to going to the journal web site, we are still within Pubmed, and that's the major difference. You are still in Pubmed. This is Pubmed central, a Pubmed database.
MF7: ok, so green means free and orange means free in Pubmed central.
I: Exactly. So now let's close out of this for now, I just wanted to give you navigation through Pubmed.
I: Let's find the original article that I sent you… and we'll go to single citation matcher on the blue band. (we click into it). Ok, so let's find that original citation in Pubmed by using single citation matcher.
MF7: And it's not a freebie.
I: Great- you're right. I got this article for you from my library and sent it to you.
MF7: Oh, that's so nice of you!
I: Well, it wasn't much, I did it on my computer from the comfort of my home.

Related Articles:
So now we are going to find more article but by using this article as the starting point, because you liked this article. We are going to use the related article tool. Using this article as the starting point, if you hit the button related articles, you'll see what happens.
MF7: 404 articles…
I: and number 2 is free.
MF7: oh, Susan Harris, yes, I know her…
I: Please check out whether it is really related to what you are looking for.
MF7: Yah, it is related. I actually don't care for this lady too much. We lock horns over some issues. But she is really well respected in the field.
I: Ok, and would you want to read something by her
MF7: yah, I usually do…
I: Ok, well have a look at the rest of the list, and see if the list is relevant to you, to see if the related articles really are related…
MF7: Oh, yes ok. Well, there are some that look interesting (number 6) but I would probably have to go somewhere to get it… related articles worked perfectly.
I: Ok, just remember that related articles searches based on the initial article- not on a Boolean search string. It's a good trick if you have something by somebody and you want to find more based on them, then you can use the related articles technique.

Books:
I: Actually click into number 6, and I'll show you something else. Please click on books.
MF7: Oh, this is different, it shows the article and it shows the abstract, and it is really highlighted.
I: Ok, so look where it used to search Pubmed what does it say now?
MF7: Books.
I: Exactly, we are now in the books database. And so, stuff that is highlighted here is mentioned in a book somewhere. It's a good tool if you need in depth information about something.

MF7: So when I read this abstract that is all highlighted, and I clicked on a term in here that is highlighted, it would take me to an article in a book?
I: Well, kind off. It may not be an article in a book, but it might be a textbook or a specialized book about something. It might be mentioned in a book.
MF7: Wow that's great. Yah, I have read articles that are part of books…
I: Well, it would be more like a book, say about cancer, and lymphedema might be mentioned in it.
MF7: right, ok …
I: So if you're looking for information that goes beyond being just an article, this is a good tool because often times, even, libraries carry these books.
MF7: Well I often go up to UBC and use the library there… its great. Now, I have a question. Underneath these publications types I see, not highlighted, but I see clinical trial and randomized control trial. They are not highlighted or anything, but that's telling me what?
I: It is telling you what type of study it is…
MF7: Now underneath that it says MeSH terms. Some are highlighted and some are not. What's that?
I: MeSH is another database. Those terms that are highlighted are in the MeSH database.
MF7: so if I clicked on that to take me to more resources that I would be interested in…
I: Nope, its not that kind of database… it is a controlled vocabulary database. Sometimes the language that Pubmed uses for something is different than the language you or I would use for something. So, if I called my arm, 'arm' they might be more specific by referring to tricep, elbow, forearm. If I were to look up arm in Pubmed it might not come up, because what I call arm, the literature might call tricep, bicep, elbow and forearm. So, then I would go to MeSH and look up arm, and it would tell me that when searching Pubmed I must use tricep, bicep and forearm, to find what I am looking for. MeSH holds all of the terms that might be associated with arm, so that if you are having no luck searching the Pubmed, you can make sure you are searching it correctly.
MF7: So it's not something that I would be using much of.
I: well let's say you weren't finding anything under lymphedema, you go check it out in MeSH and see if possibly you weren't searching correctly.
MF7: Oh I see…

Linkout:
I: well, if we have the time to look at it we will, but we have some more stuff to look at. Click on links, and go to linkout…
MF7: (She reads what it says)
I: This tool provides a list of ways of arriving at the article. It's a list of publishers, centres institutes libraries. Etc..
MF7: It says full text available through Ebsco, Ingenta.
I: Well that's important to know because they are two publishers that UBC has links with. So this tool tells you the publication information about the article, if you ever wanted to get it. You could even order it online.
MF7: Very, very useful. It also says documentary delivery Infotrieve.
I: If you do want to get your hands on an article, you are dying to read it and want to pay for it, that's a service that will get the article for you.
MF7: Oh really!
I: Click on it and see what happens.

MF7: It says if you have an account, you can do it with a credit card. It says at the bottom that service is 12 copyright, and 27 for the service is. So I have to pay both?
I: Yes you would…
MF7: So depending on how valuable the article is…I might be willing to do that.
I: Well, that's how you would do that.
I: Ok, Click on libraries now… this might be where you can go to the library to get the article.
MF7: it says ABCDEFGH…
I: Click on U.
MF7: As in University? [goes through the list to see if UBC is on it]. U of T has this article… that is pretty interesting.
I: Ok, just to move of us along…
MF7: I understand, we have a lot of information to cover in a short space of time, so I understand.
I: ok, so that's a little about links and related articles. So you have an idea of how that works…
MF7: Yes, and I am really pleased with it!
I: Great, I'm excited about that because you're going to write back to me in a little while telling me about how you go on Pubmed everyday to search the literature [joking].
MF7: I'm going to tell you it's pretty encouraging to find this, its not intimidating.
I: great. So we'll go back to the search results.
MF7: Ok, so I'm back on Anderson's article.
I: well, you're back at the abstract- or the citation.
MF7: err..
I: I just want to re-emphasize that these are not the actual articles here- this is information about the articles. Just want to be really clear on that.
MF7: Oh, ok. Oh yes, right…

Limits:
I: Let's do limits. Initially you emphasized how you want to find articles that are current. So click on the tabs up at the top of the page. Click on limits please.
MF7: [reads what the page says].
I: So Limits allows you to drill down in a way that is different than the BOOLEAN way that used at the beginning of the hour. Its doesn't limit by theme or topic, but rather by what we call 'meta data', which is information that all articles have- i.e. a title, an author, a language, a type of study. Pull down the drop menu on 'subsets'. You here have a choice between different journals. Select core clinical journals. This means we will be searching only the core clinical journals as opposed to all of the journals in the index- of which there are many. In our case for example it will exclude all articles/journals in the complementary medicine part of the index.
MF7: I like keeping it to core clinical journals because they are well recognized in the medical community.
I: That's the idea- if you choose to limit to CCJ, then you will be limiting the scope of your search. Do you see why that would happen?
MF7: Right…languages, should we put English?
I: Sure- and we'll put women for gender, and the more we put on the more we will limits and thereby get fewer results. (We put more limits on: Entrez date - one year. We select review for publication types}. Now we have our limits on, so let's do a search.

MF7: Where is the search part?
I: It's in the same place, up top.
MF7: So just hit the go button?
I: No, you'll have to put in Lymphedema.
MF7: Oh, ok.
I: Not bad, we got two! Have a look and tell me whether these would be useful to you
MF7: The first one may be of interest, and the second one I am interested in but I am not sure what it really is about…with the embedded rings.
I: Ok, I have a pop quiz for you… what's the problem with the second one?
MF7: Ahh! No abstract! And you've got to pay bucks for it.
I: Exactly- you have no substantial way to tell what this article is about. But how is the first one?
MF7: (She reads the abstract). It is moderately useful.
I: Ok, well, a few more things: do you see the yellow band across the top of the page?
MF7: Oh, limits, ok.
I: That indicated which limits were on…
MF7: Ok, all the things that I clicked.
I: Yes, it serves to remind you what you have on. So you can take things off if, say, you haven't gotten enough results. Play around with them. You also have to remove the limits yourself- so be sure to uncheck limits if you want to do a completely new search. Otherwise they stay on.
MF7: Gottcha.
I: One last thing on the list: How to save things.
MF7: Oh let's go to the article that you sent me.
I: Which one?
MF7: The article you emailed me.
I: Well, the only problem with that is that…. Well, did you erase it?
MF7: Nope.
I: So its still somewhere on your computer then? Because if so, I would prefer we found anew one, since you already have the other one.
MF7: Oh I see. Ok, how about #19. (It's a free article).
I: Ok, select it and scroll back to the top, and look for 'send to'.
MF7: I gotcha.
I: Ok, click on it, and we'll send to email….
[we do it and go to another screen, we put in her email address]
I: now if you wanted to select more than one, you would just check off more than one article.
MF7: Wow!
I: it sends the info into your email in box. So you can send the information to yourself, and go up to UBC, check your email and find the citations there. Or ask the librarian to help you… no writing it down.
MF7: Cool. So let me just say this back to you: for the ones that are not free, I could take this list to UBC that I emailed myself and get the articles, and bring it to the librarian.
I: Well yeah, it's a good way to send the information to yourself instead of having to write it down. Or when you do a presentation and use information from a paper you often times need the citation information to give to people, so this is a way to keep that information. The other thing that its used for is if you find an article and you don't have time to look at it, you save the information in your email inbox and you go back to it. It

allows you to retrieve it again.
MF7: Wow.
I: So you can go back into single citation matcher and find it through there. Ok, now let's check out another function. Go down to 19 again, and click of a bunch of citations. Go back to your send to function, and slide it up to 'clipboard'.
MF7: Ok, it says '4 items were added to clipboard'.
I: Exactly.
MF7: Ok, but where is my clipboard on my computer?
I: Good question- this is not your computer's clipboard. It is Pubmed's clipboard.
MF7: laughs- ok, I was worried there for a second.
I: Ok, so let's say you have been doing research for a presentation somewhere in Europe, and you are finding these great resources, and you want to put the information somewhere as you go about your searches. So, you save thing to clipboard as you are doing your searches. You might go for lunch for an hour, come back, and you would still have the items on your clipboard. It's a temporary storage place.
MF7: Ok, so now we go to clipboard (the tab up top). And it says items 1-5, and it re-numbers them.
I: Ok, now go to the send to function and go to email,
MF7: Hey, my email stayed in there.
I: It has a memory – so hit mail once you are ready. It'll email all those citations to you. Ok, we are actually done with that.
MF7: Thank you so much, I so appreciate your time!
I: It's my pleasure, you are helping me out…
MF7: It's very cool.
I: Do you have any questions?
MF7: I did have one, but can't remember… shoot. Ok, I don't remember.
I: Well, feel free to email me anytime you want if you have questions. Its better for me if you ask, I want to know what your questions. I might email you too, and ask you for an update.
MF7: That's perfectly ok.
I: Alright, have a good trip bye.
MF7: Thanks a lot, ok, bye.

As follow up:
The next day I sent MF7 a resource on Boolean- clarifying precisely what it does and how to do it, the various rules. She wrote back immediately and as she states: "Boy! You read my mind! I was just wondering about the use of the word OR, and thought I'd check out Boolean on the web, and here's you with the helpful info!".

Interview with MF8:

Initial comments access to the articles:
MF8: [It]would help me big time. It deters me big time having to pay for articles. I don't know if it's a monthly subscription or what, too, for buying some of them.

Current access status:
MF8: No, now that I have graduated I haven't been back in there. Umm, I can't remember if they said they might be able to access some for us or not, but I can't think of any.

The Interview:
I: Have you ever been to Pubmed before?
MF8:I don't think so… I mean I may have originally when I was in school, I just opened it up here, but I don't remember them.
I: Ok, start by going to Pubmed.com.
MF8: Ok… but the URL changes to a much longer one. How come?
I: Good question. It's called a redirect. When you put in the Pubmed URL it changes automatically to this address.
MF8: oh, ok.

Searching:
I: ok, so this is the homepage- there are different databases of information within this one big database, and there are many different points of access to the different databases.
MF8: Ok, gotcha.
I: ok, see for example there is the drop down tab where it says Pubmed? If you pull it down it has the many databases. See there is something called books and Journals and MeSH? Books is a database with information about books in it, journals is a database where you can find info on journals, and Mesh is a controlled vocabulary database?
MF8: Tell me what MeSH is once more?
I: MeSH is a controlled vocabulary database. And what that means is: Pubmed has decided how to index and categorize different subjects and themes according to a particular vocabulary. So, let's say you are a chiropractor and you are looking up "chiropractic'. You wouldn't be able to find anything if chiropractic was referred to by Pubmed as massage. So you would go into MeSH and look up how Pubmed refers to chiropratic. You would find that instead of putting in chiropractic, you would find more results on your material by calling it massage. Not a great example, but it is like a synonyms dictionary. Does that make sense?
MF8: Kind of, I guess.
I: how about we start with a search? What would you like to search?
MF8: We could search fibromyalgia.
(We type it in and find 3600 items. I explain elements of the citation to her. I start with the icon of the paper: she already knows how to distinguish whether there is an abstract and whether an article is free. We find a free article on fibromyalgia.)
MF8: The only problem is that the ones that are free are not the ones I was wanting. Naturally.
(We look at number 28, a free article, but it is not what she wants. At this point it is necessary to 'drill down'. We need to be more specific.)

I: What would you like to search for?
MF8: What massage techniques are valuable for fibromylagia, and what studies have found common causes and links between patients. I read in one of the articles about the trauma that fibromyalgia patients have experienced. Those sorts of things.
I: So links between fibromyalgia and massage therapy techniques. What are the commonalities between people that have fibromyalgia. Pain and suffering and past experiences.

I: (I explain a basic search. I then explain Boolean to her. She has never heard of Boolean before. We try 'Fibromyalgia AND massage OR chiropractic'. We are limiting the scope of the search, and widening the scope of massage. She seems to understand the explanation of Boolean. We stick with Fibromyalgia AND massage. We get 31

results.
MF8: Wow. I see the difference (referring to the difference between the searches).
I: Ok, so have a look at the results and let me know whether they seem useful to you.
(We choose number 8, and look at the abstract.)
I: Does the abstract provide sufficient information?
MF8: Definitely need to read the article. It does not tell us enough here.
(I go over the functions available on a citation: title, abstract, name of journal, and the button to get to the article, should we have access.)
MF8: Can I ask a question: I am trying to remember what the number is (referring to the vol. and issue). What is that again?
I: those are the volume and the issue and the page numbers. The author is in blue and this signifies that they are linked up to other articles who have written other articles. Also, note the different types of publication types: review versus clinical study. This one is a review study.
MF8: Ok.
I: I also want you to notice the related articles link, and then next to it something called links.
MF8: I see.
I: Ok, click on links and tell me what you see.
MF8: Mine says Books and Link Out.
I: Ok, go to link out and tell me what you think this is…
MF8: Well, full text sources: it must be where I can go to get the article full text.
I: Well done. Linkout will show you how to get to the article- like if you have to go to the publishers web site, or even if a library ahs it somewhere. Do you see the libraries list over there?
MF8: That seems useful.

Bookshelf:
I: ok, so what do you think is going on here?
MF8: Its got the abstract with a bunch of words highlighted. Lets see, what if I go to massage therapy…
(I explain books indexed in Pubmed that have keywords linked to other sources. I explain to her that she could order whole books related to her subject matter, and that she look for books in the Books database that I showed her earlier.
MF8: Ok, so they are actually books, not just the articles?
I: Exactly, you might even be able to find the books somewhere else – like on Amazon or something.
MF8: Ok.

Related articles:
I: Ok, related articles is useful to broaden your search beyond just the search terms we put in. It could broaden the scope beyond the initial search so its good when you are not quite hitting your gold mine. (We hit related articles, and I give her a minute to look over the search results.)
MF8: So, it's bringing up other articles related to complementary medicine because that's what the main title was for that article. Umm, but I am sure that if I had chosen a different article in the first place, it would have ones that be more specifically related to what my interests were. There are ones here that are really interesting here, that I am sure I would go searching through because they do seem quite related.
I: By the way, I see a lot of article by 'Ernst E', that was our initial author, that's how this related articles function works. It often does the search by author or by subject matter. But basically, you want to find an article that is almost useful or perfect, and then you

want to hit related articles. Every time you use this function, in relation to a particular article, it might take you in a new direction…
MF8: Yes!
I: Do you see what I mean? You can't just look at an entire list and use related articles. But you use one article to generate a list of related article.
MF8: Yah – I get it.
I: Actually, there is a free one here… are you interested in it?
MF8: Yes, I see it- but it is related to complementary medicine. Because complimentary medicine was in the title of the one we started this search with.
I: Very good! So, you're not interested in it?
MF8: But it is free… and there is also number 14 that is free- that one looks good too.
I: Ok, so put a check mark next to number 14 and then I want you to take a look at number 19.
MF8: Yep- that looks interesting!

I: Ok, so now let's hit related articles on that one, and do you see how it puts at the top the one we were originally looking at …
MF8: Yes.
(We look through this new list for free articles, and find one free article. We click on it.)
MF8: So, when I check marked the article before, I didn't know how to go to them after that …
I: Yes, I am going to explain that to you right now. So I want you to hit this [free article], but you might actually want to read it. And then I want you to go to 'send to'. Do you see it up there at the top? Well, with this function you can send the article or articles you selected in a couple of different ways. You can send it to your email, you can send it to the clipboard, you can send it to a file or a text file. So which would you like to do?
MF8: Umm, I don't know… sure.
I: Ok so drag down and select email. By the way, have you even done this before?
MF8: No … I haven't.
I: Great, I love that! Ok, so now up pops up the option of what information we want to send to our email. We can send the summary, the brief, the abstract, the citation, the XML "basically you can send anyone of those elements to your email. Most people tend to send the abstract, and you send it as an html or a text file. You can put a little reminder in the text box there to remind you what exactly its about. Put your email address in, and then hit mail. And it will arrive in your inbox for you to read… one day.
MF8: Yah… ok, there we go, mail, alright.
I: Then other thing you can do is send it to 'text'.
MF8: Oh yes.
I: Basically doping this takes all of the extra formatting out of the page, and it makes it a lot easier to save on to your computer, or send along somewhere. Now hit back, and lets go back to the abstract, and we'll try out send to clipboard. Are you with me?
MF8: Yup.
I: Do you see a pink ribbon at the top and within that it says one item was added to clipboard?
MF8: Hang on a sec, I am just trying to type out the directions at the same time. Ok, so you said one item was added to clipboard, yep- got it.
I: Ok, so know how you said before that you have trouble because you check things off and you don't know what happens to them afterwards?
MF8: Yes, ok.
I: Well, this is what happens, if you check them off and then send them to your clipboard, those items, citations, remain on your clipboard until you are ready to do something with them. So may be you want to email all of your citations that you have checked off as you

have done your research at once, or may be you want to send them all to text, and send them to a text file onto your computer. Sending to clipboard allows you to accumulate a number of citations, and then sift through them afterwards. Notice that when you save things to the clipboard it says: 'clipboard items will be lost after 8 hours of inactivity.' This means that if you take a break, make sure you get back to your computer 7 hours and fifty-five minutes later if you have left something on the clipboard that you do not want to lose. Do get what I mean?
MF8: Yes, except I don't know how to access my clipboard.

I: Oh, I'll show you- good question! Do you see a bunch of tabs at the top of Pubmed? They say 'limits' 'preview' 'history' and…clipboard details?
MF8: Oh, ok .. I see it.
I: Right so if you click into this area you will be in the clipboard.
MF8:I was thinking, oh I don't know.
I: Yes ... I think we are used to using MS Word, where we don't see the clipboard too easily because we don't save thing to a clipboard- we aren't sending information back and forth, but here you can actually see it. So ultimately you can compile a list of resources as you do your searches and save it and go through it, and there it is on the clipboard.
MF8: Very useful—so then you can put a bunch of different ones on there or something?
I: Exactly... so what you can do hypothetically is lets say you keep on using the related articles function, like you keep on clicking through the searches RA pulls up. Every once on a while you find an article that is interesting to you. All you have to do is check it off and send it to the clipboard and then keep on going so you are not interrupting your flow of work.
MF8: Yah – that is very useful.
I: Ok, so far we not found a totally awesome article. May there isn't anything that suits you, but may be we can search better. One way to do this is by hitting the limits function, located near the clipboard function. Do you see it: limits?
MF8: Oh, ok.
I: So click on that and basically instead of limiting using the Boolean terms we are going to limit by what we call meta-data. Do you know what meta-data is?
MF8: Umm, no.
I: That's ok- you are quite 'normal' for not knowing what it is because it's a pretty technical term. But, in essence, meta-data is information about information. Every article in this index has some basic information to it: a title, an author, a language. Not every article has an abstract, but many do. And these categories are examples of meta-data. Does that make sense to you?
MF8: Yup.
I: So in 'limits' we are going to limit our search by playing around with the meta data. So let's start by limiting by language- let's say you do not want any article in Japanese – or better yet, any articles in a language other than English. So, hit English, and you will get rid of all of those extra articles that aren't useful to you because they are in other languages. You can limit to humans or animals as the research subjects- so humans.
MF8: Yup.
I: Abstracts: this is an interesting one for you. As someone who does not have access to full text information, it might be useful to limit according to abstracts. If you do this, then you will only pull up articles that have abstracts associated with it. And you are reliant on the abstract because it is the only way you have a sense of what the article is about.
MF8: Yes.
I: There is limiting by gender- so if you want to research articles about either men or women. But we'll keep it gender neutral.

MF8: Yes.
I: Subsets – it for people looking for something more specific. Like a separate section of the database. If we choose Pubmed Central, we are guaranteed to find articles with full text as PMC is a full text article database. The caution here is that the nature of the articles are often very scientific, or it is older information. And for publication types, we get to choose, like, the genre of the article. Perhaps you want cutting edge research trials as opposed to a review of the literature or an editorial that is mostly the opinion of the writer. We'll keep it as regular- i.e. no limiting of publication types.
MF8: Ok- I need a little bit of time. Publication types, can you repeat what you were saying about that? I didn't get it.
I: Sure thing – publication types, well you know how there are different types of articles? Some are research studies, where the writers actually discovered something new, and others are some guy writing his opinion about a certain topic. These get classified. Like, the first would be called a clinical trial, and the other an editorial. So the index basically classifies each article according to what type of article it is- be it a research study, an opinion, or even just some guy summarizing the existing literature on a topic after he researched it in the library.

(MF8 is not familiar with the different types of literatures available to researchers.)

I: Now... I left the limits off of publication types because I was concerned that
MF8: We wouldn't find anything…
I: Exactly – because we are running out of time. So, to summarize: we have English, Humans, and Pubmed Central as our third limit. We could also do ages – do you see that?
MF8: Yah- I see it, but, yah, I don't know.
I: Well, it could be good for you because let's say we could look for fibromyalgia only in adults.
MF8: Yah, but the adults are just 19-44, or 45-64 and blah blah blah. But it doesn't really count 19-80.
I: But it does say 19 plus…
(It was in a hidden drop down box that Laura did not see or explore originally).
MF8: Oh there it is!
I: Alright, should we put that on?
MF8: Sure lets do it.
I: Ok, now we'll search fibromyalgia.
MF8: Ok, so we're doing this up in the search thing, right?
I: Yah- once you have out your limits on, just go straight up to the search field and hit go after you have put in your search term.
MF8: Six articles – and they are all free.
(This is because we searched in Pubmed Central. We look through the articles for good ones, and we choose number 5.)
I: ok, so this is free in BMJ. Do you know what BMJ is?
MF8: Oh, hold on a sec. What did I do? I must have gone to the next page. Ok, BMJ…
I: BMJ is a very high ranking journal called the British Medical Journal. Its online, and they make some of their articles available online for free. Now you saw that some of the articles had both an orange and green bar on the icon.
MF8: Yup.
I: Basically the orange signifies that article is available not only online, but online within the Pubmed environment. That is, Pubmed central. So it's not only that you have to go to another web site to access the article. But you can stay within the pubmed interface. When you see a green bar, you can click on the icon, and another browser window will

open with that article open, but it will have taken you into another web site or location. But with this you stay within Pubmed- so you have at your fingertips many of the tools and functions that you would have within Pubmed.
MF8: You said it in a different way now, so doesn't it just mean that um, its within Pubmed so that you can access the free full article staying within Pubmed, and you don't have to pay for and order it through somewhere else an all that?
I: Yes, but a green bar would mean that as well. But a green an orange bar mean so you can stay in Pubmed and get it- it's actually in Pubmed, or what is called Pubmed Central. And when you get more accustomed to the interface, you'll realize how valuable it is to be able to stay in Pubmed, not get lost, and also take advantage of the Pubmed tools that we have been learning today.
MF8: Ok- I get it now.
I: Ok- so, let's take another glance around here. Do you see where it says limits up top?
MF8: Ok, I think I highlighted and went to the abstract, so I better go back to where you might. Are you on page one where it says one to six items?
I: No, I clicked on the icon.
MF8: Ok, there it says limits. Yes, I see.
I: So, I just want to show how the limits that we placed on there stay on. And its just a reminder to you that you've got limits on, so that the next time you do a search you know that they are there. You actually have to take them off yourself. If you leave them on, and forget, then you won't get full searches. So that's just a warning for you.
MF8: So, if you do a different search at a different time, they'll still be there?
I: Well, I'm not sure about that; if you close out of Pubmed, they go away. But some people stay on this thing for hours at a time, so it is always good to remind them that they've got limits on so they don't commit suicide or something if they aren't getting good searches.
MF8: (laughs).
I: Now hit the free full text article. Hit that. And now it is going into Pubmed Central, and it is giving you the article. So take a look around at it.
MF8: right.
I: It gives you the article in Pubmed. Do you see what I mean? They have tried to make the article a little more accessible. So finding something in Pubmed Central is a good thing.
MF8: Nice. (she takes notes on this).
I: So, do you see how this opened up in a new browser window. So you can minimize it for now and read it later. Or notice on the side there is a whole bunch of functionality: "Pubmed articles by Mcfarlane Macbeth and Syllman", who are the writers of the article. So lets do a little test. Close out of this window and we'll go to the other full text web site, the BMJ. See there is two buttons available to us for this article. Got to the blue button and click on it.
MF8: Oh, man, what did I do? Oh, ok… I got it – I thought I had x'd it instead of minimized it. Ok. Here were are. Yup- I opened up the BMJ.
I: So, now we are not in Pubmed anymore, we are in BMJ. And it is the same article. It's the same information, and there is some functionality offered by this web site- its just not Pubmed- its different. You might be able to get some other info from it- it has related articles function too. And it also brings in ways to get back to Pubmed. Does that make sense to you?
MF8: I've just got to find it here- oh here it is. So there is stuff down the right hand side. And I see where you are. Yes.
I: So this is the functionality offered by the BMJ web site. And what they are trying to do is make it as easy as possible to link up between different articles and information. I just wanted you to note the difference between the green and the orange. One is that it takes

you to the journal web site, and one is that it takes you to a Pubmed web site. One isn't necessarily better than the other as it looks like journal web sites are beginning to include similar tools and functions as those available in Pubmed.
Ok, so x out of BMJ, and I now I want you to email this article to yourself, without my help. It's a pop quiz.
MF8: [laughs]. And begins to do that. Should I send the abstract and the citation? I can't send the full article to myself?
I: No, you can send the citation will allow you to look up the article again.
MF8: Ok, I'll just keep the abstract. And ok, it has been sent.
I: Awesome. Ok, now I want you take the limits off.
MF8: Alright, let me see. Ok, I clicked the check mark off. Does that change it immediately?
I: Check it out: click into limits and see if the check marks are still there.
MF8: No, not there anymore.
I: Ok, that covers most of what I wanted to show you. I am having a little trouble with follow up – so if you ever want another session or feel like you need something- even to go over the exact same tools, feel free to email me whenever you want.
MF8: Great. Thanks.
I: Actually let me go over one other thing with you. Sorry- once I get going I can't stop. Do you see the blue band on the left side of the page, and there it says single citation?
MF8: Yes.
I: Ok, so let's say you find the end all and be all of articles somewhere else and you want to find it in Pubmed. Here you would put in the journal title and the author's name and the words in the title, and hit go. So let's say you are trying to find a particular article that you already know about, and you know some of the citation information. This is how you would find it. Does that make sense?
MF8: Yup.
I: Ok, so that's single citation matcher. Then there is batch citation matcher.
MF8: Ok, what was the name of that again? Match citation matcher?
I: This enables you to find a bunch of citations - which I don't really know about. So we can skip it. Let's go quickly to Mesh database. So this is the database with the controlled vocabulary. Do you know what that means?
MF8: It means it's like a synonym… [laughing].
I: Right- I know you know what it is [because I had told her earlier], but as a concept…"different terminology for the same things. Ok, so what you would call exercise I would call fitness. Does that make sense to you?
MF8: Yup- it does.
I: Ok, so now we are in the Mesh database- notice how it says that at the top of the page? And how the symbol is now a tree. …
MF8: Ok, I see it the little tree.
I: Ok, so let's look up all the words for massage. Type in 'massage'.
MF8: one of two articles!
I: no- see these aren't articles.
MF8: oh, right.
I: Ok, so click on number one. These are all alternate ways of looking up massage in Pubmed. Reflexology, therapy, rolfing- I don't know what rolfing is...
MF8: It's a technique of bodywork.
I: So what you can do is if you are looking something up, you can use these terms too. So you can say 'massage' or 'therapy' or "zone threapy' or rolfing, because you know that Pubmed uses all of these terms when they are talking about massage. Make sense?
MF8: Yes.

I: Good, let's look up another thing. What else is good? What about knee problems? You must get tons of knee problems?
MF8: You mean look them up in Mesh?
I: yes
MF8: Well, what about patellar tendonitis?
I: I think that sound perfect.
[we put it in and look it up]
MF8: They said no items were found.
I: what about tendonitis? How do you spell tendonitis?
MF8: Aren't you premed? [laughing].
I: [laughing] nope I am in education.
[she spells it for me]
I: Ok, so look what we get. The blue word is the one we put in. then they give us the definition of what it is. And we say- yah that's exactly what I mean. So, we click on it and it'll show us those other terms that we can use if we want to look up tendonitis.
[it gives us a variety of spellings.] so these are what you would try if you needed to look something up about tendonitis. Let's look up low back pain.
MF8: Ok.
I: Once again it gives you the definition, just to be sure it is what you want to look up. Click on low back pain, and then it gives you the various words.
MF8: Ok, I'll see that in a sec, I am just making notes. Back ache, back pain low, yah I see that. They don't even have lumbego…
I: Oh yah, its there…
MF8: Oh yes, now I see it!
I: Yes, you can't outwit Pubmed!
[we laugh]
I: So that's incredibly useful – how to you capture exactly what you are talking about…
MF8: Yes, that's awesome, very useful- there I said it.
I: ok, so now that is really all I wanted to show you. I hope that was helpful. Again if you want to follow up in a few weeks just shoot me an email and we can do this again.
MF8: I'm sure I'll have a question here and there. You guys just do Pubmed? Not Medscape? Oh actually I do have one other question. I think I looked up on Pubmed, ummm, oh I might not have looked it up that way- it was the layperson way of doing it. It was joint manipulation and development of osteoarthritis.
I: Yah…
MF8: It came up with nothing. But when I Googled it and it came up with a bunch of stuff. But it was more like cracking knuckles or something .. umm, are you still there?
I: Of course! Do you want to do it through MeSH?
MF8: oh yah, that's a good idea…
I: So what is it?
MF8: Cracking knuckles and the development of osteoarthritis.
I: ok, I would do cracking knuckles first because we aren't finding the articles yet we are just finding the terms.
MF8: Yes, it didn't have any items found. What about joint manipulation? I am sure they'll have a zillion. Or, high velocity manipulation? No items.
I: Hold on a sec… I don't see anything here. So it is cracking knuckles and…
MF8: the development of osteoarthritis. But I can't see how they are going to have that when they don't have cracking knuckles. Let's see… no. No items found. You know it doesn't matter umm, it found a whack of articles listed by just doing a general google search, and then reading a few articles, or like ok here is one study done and they cited another so I could probably search for those specific studies and read them myself.
I: Yah, or you could put the study into Pubmed and do related articles

MF8: Yah that would work. I'll just make a note of that (types). Cool, that's helpful. Thank you.
I: So do you think you'll use Pubmed? Do you think it is helpful?
MF8: oh, god yes.
I: really? Like, everyday?
MF8: probably not because I don't have it at work… but I'd say probably weekly anyways,.
I: ok, do feel free to send me questions when you have them. And it was great meeting you.
MF8: thank you so much for your help.
I: Ok goodnight
MF8: ok, bye.

Interview with MM9
I: Is it ok if I tape record you and put you on speaker phone?
MM9: Sure thing.
I: ok, so the first thing to do is to find out who you are. Can you tell me a little bit about yourself?
MM9: Sure, I am a licensed massage therapist licensed to practice in British Columbia. Freelance occasional writer in alternative and conventional medicine having published in several journals of both kinds. I also do investigative journalism.
I: So you've done a lot of research like this before?
MM9: Not a lot…but I've used Medline and Pubmed, Netscape. I don't do a lot of those kinds of research I don't spend a lot of time on those web sites. But I'd like to learn.
I: great you'll find Pubmed useful. Sounds like you would really want to use the literature that already exists.
MM9: Yah, I would.
I: And your computer skills. How often do you use the computer?
MM9: About everyday.
I: do you surf the web everyday?
MM9: Yep, I do. I use Google a lot. I'd sure like for Google to find things faster. I'd like it work a lot faster.
I: Do you ever go to SFU or UBC and the medical libraries there?
MM9: Yeh, I have gone to UBC and umm, there's an extension library on 11th or 12 avenue near the hospital that I use. I visit the university campus from time to time but most of the ones I use are downtown near the hospital.
I: So, basically you do use the libraries?
MM9: From time to time. I haven't had to use them recently though.
I: How long in Massage Therapy?
MM9: In BC since 1983. I was licensed in Ontario in 1982.
I: Do you have any specialties in massage therapy?
MM9: well there is no such thing as a specialty in MT, but if you mean special interests, back pain, orthopedic.
I: ok, so the two articles I sent you… so you found one to be helpful, and one to be too medical?
MM9: No, the other one was too boring. I mean as a massage therapist I'm not likely to be dealing with drugs or hearing much about anybody's drug history and yet we did have a little bit of background and continuing education on painkillers but it was a voluntary course and I wasn't that interested in taking it. I'm not really interested in

pharmaceuticals. I find them I mean they don't help people they kill them by the hundreds. I certainly don't advise people to take pharmaceuticals.
If someone's got symptoms of something, and I know what drugs they are taking, I might go to the extent of looking them up for the patient or if I suspect they might have a problem I would tell them to go call their doctor or pharmacist immediately, I have had that on occasion. So that's my interest in drugs.
I: ok. In general then, in terms of the fibromylagia article, can you just give me some basic thoughts of it
MM9: well FB is easy to write about because there is always something new on the subject. I've read a lot of them over the years. I have treated people with FB with people that either knew they had it or didn't know they had it because of the trigger points. I find it interesting that there is literally something underneath your hands can have so many takes on what it is. And everything from drugs to massage or whatever. I am interested because it is interesting to see how people are looking at the same thing. If I had FB I would be very frustrated because I would want to find the real answer of something that actually works…so I don't find this a conclusive article at all but it is interesting to read about.
I: Ok, great. Sounds like you have good critical reading skills.
MM9: Yah, I do, if you told me I had to write an essay on this I would be coming back with a lot more precise information.
I: No I just want your thoughts, so that's great. And your PS education?
MM9: BA from Reid college, in Portland Oregon. I did professional modern dance training at the Vancouver School of the Arts, two years later I took two years counseling psychology courses in Victoria. Of course I went to the Canadian College of Massage and hydro-therapy taken umpteen post-graduate workshops. I have had Anthony Robbins personal power training.

Homepage:
(We start at Pubmed.com. We start off with basic things, explanation of the Pubmed web site.)
I: Which would you prefer to look up, back pain or Fibromyalgia?
MM9: Back pain
I: Ok, so type back pain into the search field.
MM9: Ok, type into where it says Pubmed, or where it says 'for?'
I: Put it in right after for.
MM9: Ok, I got back pain. There is going to be a billion of articles. Yeh, really, there is 21 000!
I: Ok so there is 21, 000 articles. 2569 are reviews
MM9: So what's that?
I: That's reviews of the literature
MM9: Oh yeh, yeh, yeh…
I: now that's too many. I just wanted to show you how the object of the game is to limit as much as possible the object of your search.
MM9: Yeh, makes sense.

Boolean:
I: So we'll start by using the Boolean search techniques. Have you ever heard of it?
MM9: Yeh, I have.

I: Have you ever done it before?
MM9: I don't think so.
I: Ok, well it's going to sound difficult but its not. If I say Back Pain AND massage therapy, I am limiting the scope of my search…
MM9: right.
I: ok write Back Pain AND Massage Therapy.
MM9: ok. Only 109.
I: we really limited our scope. It means only 109 articles mention BP and MT.
MM9: Very nice.
I: let's say we limited too much -- we do OR. MT AND BP OR chiropractic
MM9: 3423.
I: We widened the search. There are three Boolean terms. And, Or, Not.
MM9: How do you do "Not"?
I: Ok, let's add a NOT
MM9: ok, so we are putting NOT at the very end of chiropractic?
I: That's right. Now what can we do with NOT?
MM9: Your'e trying to make it over, like not drugs or not whatever?
I: right. Why not NOT drugs. How does that sound?
MM9: Ok. Ok, so I have got chiropractic NOT drugs, and it's the same, 3369.
I: umm, it's a little different, we limited a tiny bit more. It shaved off a few. It cleaned out anything that's got drugs in the article.
MM9: Right.
I: So that's an example of drilling down and limiting scope right there. They are basic library skills that do not pertain only to this database but to all. Even Google.
MM9: Yah, this is good.
I: I found a doc on Boolean search techniques for someone else and I can send it to you if you want a visual diagram of how they work.
MM9: Ok, sure. But doesn't Google. Have that? I think I went through some sort of study thing there I can't remember. You know, it's advanced search and then you click on something and then it'll tell you how to set I up …
I: I think you are right, you can do it in advanced search.
MM9: Yeh, I think so. Anything you got that makes it simpler you know that would help because some of this I do late at night, and just going through Googling is just too tiring.
I: Ok, I'll send it along. And you can also play along with other things like brackets and starts.
MM9: Yeh, what does that do? Brackets and stars?
I: Brackets keeps two search terms together. (I explain more using the example of Massage therapy).
MM9: So you bracket massage and separately therapy?
I: No I think you bracket it as one term so that any article that has it as one term comes up… but we should look into that more.. I am not an expert in it. But there are other Pubmed tools that will really help limit searches in a similar way. It is a good idea to know about the basics though … a librarian could help.
MM9: Ok.
I: Ok we'll go back to our initial search of back pain and massage therapy ..
MM9: What, oh, ok I'll just erase the whole thing and start over.
I: Sure thing.

MM9: It’s on my little data bar here at the bottom. (He types). Ok, so what was it again? Massage therapy and?
I: Back pain.

MeSH:
MM9: What would happen if I typed in “back pain” as one word?
I: hmm, try it good question.. using that question as an example lets try something interesting. Click on MeSH database in the blue bar to the side.
MM9: Oh yeh right.
I: This is a controlled vocabulary database. (I explain MeSH). We’ll see all the different ways that Pubmed refers to back pain. See it says search MESH
D: Ok so I a spelling this as back pain?
I: Yeh, two separate words.
MM9: Ok, I got two things, low back pain and back pain.
I: Right, so basically it categorizes back pain in a couple of ways.
MM9: right.
I: So we could choose to write back pain as back pain or low pain. Which one do you want to look at?
MM9: Low.
I: Ok, so click on low.
MM9: Ok, do you want me to check the box or just click on…
I: No, don’t check the box. Just click on the word.
MM9: Ok, got there.
I: Ok, so there is different ways of, well different people would refer to back pain in different ways. If you were a medical doctor you would think of it in one way, a psychologist you would think of it in another way.
MM9: Or more to the point, if you were a chiropractor and a medical doctor you would think of these things in different ways and I am curious, it may be political if they had set this up in such a way where the word ‘subluxaction’[1] does not occur here in any way. Where that word in itself means something quite different to a medical doctor then it does to a chiropractor when it comes to small tiny restriction in motion or small tiny misalignment. But the medical profession still doesn’t believe such things can occur so I see here that there is not such thing here as ‘subluxation’ in the sense that the chiropractor means it – very interesting.
I: Yes, exactly, this is why we want your feedback. So that’s a great point, so, it is political. Ok, so basically you got exactly why there is a MESH database. We are all separated by our disciplines. And therefore we use language in different ways.
MM9: Yah!
I: But sometimes we want to find things and cross from one discipline to another …
MM9: Well, why is there, I am noticing down here at the bottom low pack and I see subheadings, and economics here—I mean what in the world … I mean umm… at the top of my head I don’t see how these would connect up. But as I think more I could see there might be a connection…yes it does take the term far and wide.
I: I don’t see that…
MM9: ok, see below subheadings.
I: Ok, yah I see it.
MM9: I am amazed at the different topics they associate with back pain. I wouldn’t have

---
1

thought they were connected but yeh, economics and curing low back pain!
I: Isn't that interesting. Great well you really got it. Scroll down and see entry terms. We could even combine low back pain in a number of different ways (I explain more). So the Mesh database is a good for finding different ways to describe what you are looking for.
MM9: I am curious: I don't see back pain as one word and I am pretty sure I have seen it that way I wonder if I spelled it wrong! It doesn't show on the list… well, anyways.
I: Do you think you would use the MeSH database?
MM9: Well yeh! Now that I see what it is.
I: Should we practice?
MM9: Well, I don't usually practice because I have an injury.
I: No just practice the search.
MM9: ok, let's see
I: Ok, try fibromylagia.
MM9: ok, just put in fibromyalgia. Ok, I only get two and fatigue syndrome, Chronic. Ok, lots of definitions.
I: Ok, so now click into the first one. It is almost like an encyclopedia.
MM9: yah!
I: So, when we looked up back pain we only got two, and then we clicked into it and we got a greater list.
MM9: Ok, so I am just writing notes here, so one of the things MESH does is give good definition (trying to pin down what it is). Yah, I guess if you are not sure if you should be looking up one thing or another, it helps to distinguish things and so you can go in one direction or another depending on what you want… what category you want …
I: Ok, so lets go back to Pubmed, go to the black bar at the top just click into Pubmed.
MM9: Ok, I am back on the homepage.
I: Great now put in low back pain into your search field and then hit go.
MM9: ok, I have got 10,929.
I: Ok, so lets look at a few things. Do you know what a citation is?
MM9: Yah, something you find in article that says John Smith wrote an article. And then you go look it up.

Citation:
I: Exactly. So you see here are many citations for articles about Lower Back Pain. Every citation consists of basic elements (I explain more). Technically this is all called metadata- information about information and it is what is being searched by the database to come to you… do you see what I mean?
MM9: Yup. You can click on the articles and see what other articles these authors have written.
MM9: Yah, I see that I just did that.
I: So if you are interested in one article by this author you might be interested in more from this author. OK, go back. Ok scroll through the list and try to find an icon with a green bar across the top of it.

Open Access Articles:
MM9: hmm, a green bar. Hold on.
I: I think you are going to like this.
MM9: Ok, hold on, just looking, ok, oh I see previous. Oh yeh, so green bar on number 24.

I: Yah, what do you think the green bar means?
MM9: umm, let me see. Uh, I don't know an abstract that is really recent?
I: Ok… no ... a little better…
MM9: [He laughs] You should have a lecture in video. Can I take the excitement?
I: Ok, click into it and you'll see what I mean.
MM9: (Reads out the article name)
I: Do you see it says full text article?
MM9: Oh! So they are going to give me the article?
I: Exactly! So the object of the game, if you don't have an affiliation with an institution or a subscription to EBSCO or Ingenta, is to find free articles. And when you see a green bar, in the icon in the Pubmed database, it means it is free. And when you click on this icon, another web site will open that contains the article.
MM9: I am just writing that down. Ok so I am going to click on that and see what happens.
I: Ok, yah,
MM9: Ok, I see what you got here … it is a whole article.
I: Yah, it's the whole article, and basically the whole journal, Minerva Medica, you can download the PDF to your desktop and you can even explore the rest of the web site if you want to and see if there are more free articles. Sometimes when you hit a journal that has a free article, the whole journal may have adopted an open access policy. You also have search functions within the journal web site so that the whole web site is like a mini Pubmed.
MM9: Right.
I: Can you let me know whether or not you would find this useful? Can you read the abstract and let me know?
MM9: the one we just clicked into?
I: Yes please. Skim through it and let me know if you would comprehend it and be interested in it.
MM9: Well not really, they are using slightly different terminology. I mean it's a bit awkward for me in a hurry, but I'd have to say, for example, "bio-psychosocial syndrome", I mean I have never heard that term before. They may have made it up, from what I can tell for the purpose of the article by the look of it, but then again, may be not. Umm, rehabilitation and water, probably within hydro-therapy in North America at least. But then again, in Europe, I mean Europe is where hydro-therapy came from. So rehabilitation and water therapy, it's a funny use of terms. But I am not sure what their use of terms is. So, I would be stumbling through this, and thinking well do they mean the same thing and I would probably go try to find something that I could understand right off the bat. Umm, what a massage therapist is looking for, this is a little too theoretical and general, so if I was trying to keep up with whatever this local focus is, I'd be looking for protocols for treating low back pain and massage therapy if there were such a thing and may be trying to get a better protocol than the one the MPA has, I'd be more on 'protocol', 'orthopedic', 'massage therapy', those types of words. And umm, at least, I think I would be. So I would avoid anything that had to do with drug approaches or psycho-therapy, though they would be important to know if they were there.
I: Good feedback. Good critical assessment.
MM9: I mean this is what I think for BC, I am not sure what is true for Ontario.
I: No, I mean I am interested in what everyone thinks. I am looking to understand more about the tools available in the database…what you will use, and what you think of the

articles.
MM9: Well, we always need to learn more, and we are less likely to want to learn more about what somebody else is doing because talk about holistic and all the rest of it, we are still not! And so show me more on musculo-skeletal soft tissue hands on massage therapy, or show me more about neurology and musculo-skeletal if you are a chiropractor, and especially chiropractic approaches. And if you are an orthopedic MD you know, its different, you don't care about the first two!
I: Yes, we shouldn't assume anything, we still have to find out more about it. So, great feedback! Ok, click on #22, go back to it, which is another one with a green bar. Look over the abstract and let me know whether it looks god.
MM9: Oh, ok. Yah this looks pretty good actually. This is the sort of thing that happens to appeal to me and I know something about treating people and from my own problems and my response is that, well first of all, the term 'meso-therapy', umm, they really need to define that, I don't know what in the world they are talking about, I assume its something like massage. We run into this when we are looking for research on massage therapy is, umm, two kinds of problems just around that. There is different, umm, designations for massage therapy. I mean you never see that for medical stuff, you see it for massage therapy. I mean a medical doctor is a medical doctor is a medical doctor. But a massage therapist is meso-therapist sometimes, or a myo-fascial therapist sometimes,
I: Or a lymphologist?
MM9: Those are different hands on work in the general umbrella of massage therapy. And then the are other problems is that massage therapy is so different around the world in terms of the training. You just can't study, one of the biggest problems in research in massage therapy is that most of it does not come from Canada, and the training for Massage therapists anywhere other than Canada and Europe is so vastly less, in terms of number of hours and depth that you can't, you know, read a study, where it says that a massage therapist did this or that because if it was done in BC you can't assume that the massage therapist would do the same thing, or do it as well. Because there is just less consistency between massage therapists then between physios. Just our training: you can go for a 100 hours in the US and become a massage therapist. Here in BC it is 3000 hours. So there is got to be some difference there when they use the word massage therapy. And, evolutionarily, we've got 3000 hours now, but when I went to school in 1981, it was 1500 hours, or 1000 hours. So it has grown and it depends on when the article was written. In terms of the article here, I am not sure exactly what meso-therapy is, but I assume it is some form of massage. But I am interested in it because this article treats a problem that I see in probably about 90% of back pain people, and most of the people that I treat are low back people and it is a difficult problem, it is usually chronic so you are left with what can I do that will help this person that is not merely symptomatic so when I read about sacro-illiac support has the best result here along with meso-therapy, those are approaches, hands on massage, that I have actually recommended, I don't agree that it is difficult to diagnose, or that its primary, and everything else comes out from there – I mean yes it may be a problem, but it is certainly related to something else.
I: ok, how about we look it up in Mesh?
MM9: Ok, that's a great idea.
I: Ok, so go to the side bar there first., so let's look up messo-therapy and then hit go.
MM9: I think it is two words. No items found.
I: Yes, but do you see what its says there? Suggestions, Hydro-therapy… thermo therapy

(and others). Click on hydro-therapy because that's probably the one you know best.
MM9: umm, surprised they don't have massage therapy there … that might be more politics. Ok, hydrotherapy. What about physical therapy techniques? Or does that not have anything do with, is it something different?
I: It is basically like a tree. So it starts off with the major category and it filters downward to what it is. So, basically hydro-therapy is a physical therapy technique …
This tree traces back or forward to the bigger categories.
(the search to understand what messo-therapy is was fruitless
(Interrupted by call waiting)
I: Ok, let's go back to the original article and I'll show you some more tools.
MM9: To the article?
I: Ok, well go back to the abstract for the article – we had never looked at the actual article itself.
MM9: Ok, I have to find it again. What number was it again?
I: number 26.
MM9: Ok, now I am there.

Send to email
I: Ok, check off the little number and we are going to practice sending this article citation to your email inbox so we can read it in the future. Do you see up at the top it has the options "…"
MM9: Ah, what?
I: Do you see at the top display abstract, show 20, sort by, and send to…
MM9: No.
I: Ok, well take a closer look.
MM9: Oh, up to the tab?
I: Up on top of that. Ok, don't click on the abstract, just click on 'send to'.
MM9: Oh yeh.
IW: Ok, scroll down and select email.
MM9: Ok,
I: great now put your email address in and hit send and it'll send the email to you.
MM9: ok I hit mail, and off it goes. OK, so it'll have a link right to this article ?
I: I don't think it'll have a link to the article, but it will have one to the citation in Pubmed.
MM9: Ok, so let's say I was doing research on a topic, say, I don't know, and every time saw something in Pubmed that I liked the abstract and I wanted to kind of put them all together I would go email, email, email, and then I could in my email system all put them into the same file?
I: Well, let me show you another function that is similar so go back to the LBP list.
MM9: ok
I: Ok, so there are three items in our list that are free.
MM9: Which ones are we talking about?
I: Yes, 24,25,26 are free. They all have green tabs. So let's say you want to look at those later on. Select them, scroll up to the top of the screen and send it to clipboard. So again it is the send to function but you are send it to your clipboard instead of email.
MM9: Yup. I did that.
I: Ok, above that it says three items are added to clipboard. So the question is now, how do I look at my clipboard, right?

MM9: Yup.
I: Ok, see on top of that there are tabs? And it says, clipboard? Click on it.
MM9: oh, ok.
I: So here is your clipboard.
MM9: I've got three of them up here now.
I: Now you can send these to your email by selecting them and send to email. So basically you can select your articles as you do your searches, save them to the clipboard, sort through them again afterwards and then send them to yourself.
MM9: oh, ok, I get that. I am trying to write it down so I can get it. So, I click them off and that lets me save them for later.
I: Right you don't necessarily need the exact article, but you need to know how to find it. So, let's say you hit on three amazing articles, all you need is the name of it and the journal info. and you can retrieve it in the future. This sends you that info and you can keep it for as long as you want.
MM9: If I wanted to save the abstracts, is there a way to do that?
I: Ok, well you go to you email preferences, when you are taken to the email screen it asks you what you want to send, what information you want to send. But I am going to ask that we move onto the next tool because we should wrap this up soon. You can always email me your questions.
MM9: Ok.
I: Ok, so selecting number 26 again, back to our original search list.
MM9: Click on the authors or click on the box.
I: Click on the authors to get into the abstract. The box for saving the citation or selecting it to send it somewhere.
MM9: Right. So I am back at the abstract.
I: Ok because you liked this article, let's say you want to find more articles like it. So let's hit related articles, on the right side of the screen.
MM9: Ok,
I: So what this tool does, if you found and article you think is great and you want more like it, and want to find more information like it, we hit related articles. So instead of specifying search criteria this tool uses that initial article or abstract to search for more like it.
MM9: So it uses source article for finding similar ones.
I: that's exactly it. It's another way of searching the database.
MM9: Ok, got it.
I: Ok, so look through the first few and take a few minutes to tell me whether these articles are really related and useful for you. Take a good 5 minutes for this.
MM9: ok. Can I put the phone down when I do this?
I: Sure.
(time passes)
MM9: ok, I've looked at 6 now. How many did you want me to look at?
I: That's great. That's enough. Ok, so what do you think? Give me a sense as to whether this would be a good shortcut to limit your searches.
MM9: Umm, right. I think that first of all, I would have to believe I don't know everything and I would learn something. A lot of us feel that way. Some don't feel they need to know more… but yes, this is interesting. I would have to have a pretty strong reason for doing this. One of the reasons would be that I myself have a back problem and I am not receiving treatment adequately. And the other might be that I have a patient that

I have a particular interest in and I want to find out more either form the point of view of doing something for them or getting them onto someone who can help them out. Even though we are not allowed to diagnose or anything. I have to be motivated in the first place to look up articles. But if I were motivated, then yes, I did like this tool. I would use it because I found some really relevant stuff.
I: Ok, great answer. Ok, now I was hoping we could choose just any article, except for a free one because I want to show you another tool.
MM9: Ok.
I: Ok, so let's go back to our search results. Click on number 3, click into it and then click into Links. So where related articles was, there was a little link called Links, and then choose Link out.

Linkout:
MM9: What does that mean? Link to a site other than Pubmed? Because I have done that before.
I: Ok, what Linkout does, is when you do not have open access to the article when it is not free, it will not show up as green in the little page icon. But you are dying to get your hands on the article… so the question is how do you get it? This Linkout function tells you how and where to get the article. Not every article in Pubmed is free, and
MM9: Yah, that is very disappointing.
I: Yah, it is disappointing because sometime you find that one article that is absolutely golden, but you can't get it. Sometimes, if the copy is an electronic article, it links you directly to the article online and all you have do to is pay for it. SO it'll ask you for your credit card number. The other thing you can do is click on libraries.
MM9: where do I find that? Oh I see it.
I: click on U in the list. This list is of all the libraries that carry this article.
MM9: It says 'the University of Sherbrooke' only carries it…oh, Toronto and Saskatchewan have it.
I: Scroll all the way down. May be UBC is on this list. May be you can ask a librarian in Toronto or Saskatchewan to fax it over to you or something. But this is a list of all of the libraries that carry this article. And the point is to show you how you can get your hands on it. In fact, lets go back
MM9: So the library might send it free or something like that?
I: Well, it's worth looking into. Let's go back to our related articles list of 103. And click on number 6.
MM9: Ok
I: And go again to Linkout.
MM9: Ok.
I: Ok this article is available again through libraries go to U.
MM9: Why U? Why are we always looking at U?
I: University of British Columbia.
MM9: Oh (he laughs) ok.
I: No question is a bad question.
MM9: Well, U goes along with anything. I mean U of Florida, U of Oregon, I mean anything.
I: Well, yes, that's true, but it's not even the longest list. Do feel free to click through the

list and see if there are other places that might be useful. Ok, go back to the search list.
MM9: I'm not getting your terminology. Ok, so the search list is the 40 – odd articles.
I: Yah, that's right, sorry about that.
MM9: But some of them are red.
I: Yeh, they change colours if you have clicked into them. Ok, there is something else I want to show. Go to 45 on the search list. So, forward a page or two.
MM9: Ok.
I: Go to linkout again. Do you see what it says " full text sources?". Click on the full text link. See how it says to view the article click on the Elsevier full science web site.
MM9: Yes.
I: Well, this is one way to get the article, but since it is Elsevier, it might not be free. Let's click on it and see what happens.
MM9: You get the abstract.
I: The question is can we get access to the full text?
MM9: Ok, so I click on full text at the bottom?
I: Yup.
MM9: Ok, yes, they want 30 bucks US. Hey that's really expensive. I can see why students anyways and poor people get ground to a halt. It is nice to have Pubmed here any everything but its hard to really get a hold of the article unless you live right next door to the right university – not just the university. If you go to the university they'll have Medline, they won't just have Pubmed, right? So they can get into these articles if they are willing to do it and then you just pay for the expensive Xerox machine and its less than 30 dollars.

Limits:
I: Yes, possibly. Ok, so that's all the different ways to Linkout. Linkout refers to find the article. Now, I think we have to cut this a little short because we have been on the phone for about an hour and half. So let's turn to limits. Going back to our low back pain search results. (Has trouble finding limits, I explain limits, I explain the concept of meta-data, we limit to English. Male.
MM9: Do we leave animals or human for male?
I: Sure, click on humans. Ok look at subsets.
MM9: A bunch of different databases about male humans in the English language?
I: Uh, subsets. When you click down, you are looking at different databases. I want you to click core clinical journals- that would be something like BMJ and classical journals.
MM9: I did.
I: (I explain more about core clinical journals). For Entrez date choose one year so we get newer articles.
MM9: Right.
I: Ok, choose publication dates, do you see that?
MM9: Hmm, (has some trouble and then finds it).
I: Choose clinical trials. (I explain more about that, what a clinical trial is). Ok let's search with these limits on.
MM9: Ok, so you don't want to make publication date to just one year, eh?
I: Well we did the Entrez date as one year.
MM9: Ok.
I: You can play around with it after we get off the found.
MM9: I got no articles found.

I: oh, I got ten.
MM9: I don't know what's going on. Oh yes, I got ten.
I: Yes, so do you see how that works?
MM9: Yah, and we are down to a reasonable number of articles to.
Pubmed Central:
I: Ok, go to number 4 and see how it has green and orange tab?
MM9: I am a little colour blind, I see orange but, oh, yes, yes. I see it.
I: ok, click into it. You can access article in 2 ways. One by going to BMJ.com, and the second via Pubmed Central – go to Pubmed Central, and basically another window pops up with the same article. So you stay in Pubmed, to access the article.
MM9: Right.
(we look at the difference between Pubmed and BMJ.com. End of recorded interview here).

Interview with CF10

Background:
(In reference to living in Grenoble and being a chiropractor there)
CF10: I was in a terrible situation of timewarp because nothing is as advanced as it is here and the wiring and such. So any of the work that I had done for the profession was done the old fashioned way. So when I came back they said would you like to teach, I realized what kind of gap I was in. So I put myself into everything ... to get with it. For me, I have come along way, but I but by north American standards I have a long way to go.
I: It's a learning curve and it gets easier.
CF10 That is very true because from what I knew before and what I know now is nothing short of fantastic! Compared to my sister who is right up there, I am back down here again (points to the ground).
I: Do you know what a browser is?
CF10: that thing there with an arrow?
I: (I go through the various parts of the computer. I explain about bells and whistles on web sites and why people have trouble using them.)
CF10: Oh that makes sense because I can understand why things don't work for me…
I: (More about research. I talk more about free access to research. Describe purpose of the study). As a chiropractor is research useful to your professional development? How would you use it?
CF10: That raises a very interesting question because patients do come in and say "there is a paper on my condition", but they have gotten it out of Google which is medical information that is very non-specific. You know anybody could put their paper out there so it is not scientifically sound. You know they are just basically narratives so that is another reason why I would like to have access to something decent. You know that research can corroborate.
I: (I talk more about the professional having to answer to the patient when confronted by a research paper).
CF10: Yah, and it's a hard one to answer to and I'd appreciate if you can give me an answer because I tell them things like that and they say 'yeh sure'.
I: Does that happen often? How often does it happen?
CF10: Often enough. You'd be surprised how often it (people showing up to her with a paper) happens. People who are really hooked on the thing they go in there and they'll come back in weeks time on their next visit and say here's what I have found on my condition. So it happens several times a month.

I: How does that make you feel, as the chiropractor?
CF10: Well, I welcome the challenge, so that's not a problem. My problem is telling them that what they are finding in Google is not that sound scientific information because any practitioner out there, anybody can put something out onto Google or their other office web site but its not necessarily valid. And doesn't apply to their case because the scientific reasoning isn't sound, but, how can I say this? They don't buy what I say? You know when I tell them that there are better sites.
I: this is a particular mindset that you as a service provider have to deal with….you have to battle information with information…you can pull rank without demeaning them. You'll see why people have this mindset when you see Pubmed. So how long have you being doing it for?
CF10: 32 years in May. I was in the Alps in Grenoble. I was there for 23 years. I have two sons.
We talk about France. In reference to the article I sent her prior to the interview:
CF10: Well that's why I asked you about the visceral part, I really need that. I couldn't get past the www.science.org because it asked me for a subscription that I couldn't get to the paper, so does that mean in here you have a system whereby you get instant access to it?
I: Well that is because this is basically a database or an index (I explain to her what these are, bring in old library searching as a comparison).
CF10: Well, I was the first librarian at CMCC when it came to the new campus. Books and writing are my passion, when I started as librarian because we knew that we needed terribly because our profession was ready to go to the national institute of neurological diseases major congress in Washington so we realized we had no indexing, so I started CRAC, which is the chiropractic research archives collection, so that was as a result of my work, so I knew that people were going to in that direction. I can relate to this then, but it is methodology that is sadly lacking.

Pubmed homepage:
I: So this is the beginning interface, this is like a gateway to different databases. Do you know what a database is?
CF10: Where you go for information?
I: Right. It contains the information. And the interface can take you there. (I explain the difference between using the index in a book or paper version vs. the online version. We start by looking up visceral pain.)
CF10: May I interrupt here? Am I supposed to know what those mean? (referring to the different databases within Pubmed: Protein, Nucleotide, Genome).
I: (I explain why there are different databases, their specificity and different points of access in web pages.) This is where we would put our Boolean search terms(indicating the field).
CF10: What is that term you just used?
I: Boolean.
CF10: How do you spell that?
I: BOOLEAN.
CF10: I have never heard the word before. Again how do you spell that?
I: Boolean. But that has nothing to do with the web. You might in fact know the principle of Boolean, but not know its name. (I explain using visceral pain and pelvis. AND OR NOT as part of Boolean vocabulary).
CF10: For simplicity sake, probably visceral pain.
I: Yes but that doesn't join two phrases so it's not Boolean.
CF10: Oh, I see.
I: Boolean terms are AND NOT OR. Do you see why?

CF10: Well because it will expand the search.
I: Well, it will refine the search.
CF10: Refine it, right.
I: And that's what your goal is, right? You have to be specific with your searches. So let's say we want visceral pain NOT pelvic pain. Do you see the logic?
CF10: Yes.
I: So what does this phrase do?
CF10: You mean in your terms or in the knowledge base?
I: Not in the knowledge base, just in terms of searching.
CF10: That it'll be only certain viscera not those in the pelvic cavity.
I: Right, exactly. What about visceral pain AND pelvic pain?
CF10: Well, we are enlarging the search.
I: Exactly.
I: Ok let's start with visceral pain and pelvic pain.
CF10: Super.
I: Really?
CF10: Oh yes. This one would be super cool. And this one would be interesting.
I: Oh yes, I read that one in terms of finding out what that meant. So, do you see how important it is to get this part right. (I give her an orientation to the search list describing: clickbox, icon—I explain green/orange bar, when you need subscriptions etc…).
CF10: How did you know that?
I: Well, because I have been teaching people about Pubmed. It is somewhere in the tutorial…
CF10: Ok, so I shouldn't feel that stupid that I didn't know that.
I: There is no feeling stupid!
CF10: That's kind.
I: (I explain the citation set up: language, title, Pubmed information, title, authors.)
CF10: Is there a reason why the authors are highlighted in blue?
I: Here, I'll show you. (I explain how the authors are linked up to their other works).
CF10: I think I can get access (to the papers) through CMCC. I am directly linked to Anne and the research. It is my homepage and I just go.
I: Let's say you don't have access – you don't have connection to the CMCC anymore. You try clicking on related article. (I explain more, what it's function is).
CF10: Would you mind if we did this to the other paper tough? The urological paper?
I: Not at all, let's do it. Ok, do you see its says click here? You would need a subscription to this article even though there is a button right here. If you were looking at this one yesterday and could not get access, it says to me that you did not really have access from home.
CF10: Well, CMCC does not subscribe to every single journal. There is a whole bunch that we have access to so we don't have to pay, and it is linked with UofT. So since I am a member of CMCC I have access. People who are not members would not have access, so I do not know how they would get passed that. But if I take the British Medical Journal, which is very relevant, to our profession- they have made their issue more specific, which means that you can only get their information within the first two weeks. After that, they charge for access.
(We go back to talking about the citation.)
I: (I talk more about browser windows and how many windows can be open at once. I then explain Related Articles, how it is another way of drilling down based on the first abstract). Do you see why?
CF10: Yes, yes, we are good there.
I: Ok, can you look at this list and tell me whether the information is useful to you?
CF10: Oh, those are all fine by me. Those are great. Especially this one would be great

too. That one is quite appropriate. I don't want to read anymore about headaches; I am headached out.
I: It is giving you a headache?
CF10: (She laughs).
I: Ok, do you see the difference between this icon and this icon (one has lines and one does not).
CF10: Yes.
I: Well when there are lines it means there is an abstract, and when there are not there is none.
CF10: Uh huh. What would the reason for that be? … it is very interesting, especially for a journal like that.
I: Well, let's take a look at it. Oh it's a review. That's why there is no abstract.
CF10: How do you flip around in the page so quickly?
I: Oh it's because I am dragging it around. Would you like to try it?
CF10: (she tries it). Oh ok, I got it. (She checks off many articles from the related articles) search)
I: So what do you think of related articles?
CF10: oh, it's great. I did find a lot of article that I want to read.

Send to:
I (I explain the send to function. We send to email. We sent 8 articles to her email. Is how her how to choose what information she can send to her email in box. We send the summary to her email).
CF10: Now the summary is what?
I: It is what you just saw in the list. Abstract, title, author… you can also write something to remind yourself of what the search was about.
CF10: Can you put visceral pain?
I: Sounds good. Do you want to write that in?
CF10: (She types it in). I do 'the seek and yee shall find' method (referring to her email inbox).
I: Ok, and put your email address in there.
CF10: (She does it and sends the information). So basically it would be a good idea to go back to email is to go and use this.
I: Yes, well I want to know those things, whether you do in fact end up using the database and the articles you get from it.
CF10: Yes, yes, ok.
I: Ok, so now do a Boolean search of a particular topic, and we'll replicate what we just did but you do it on your own.
CF10: (thinks for a minute), Ok, visceral pain AND women. (She types it in).
I: Will this expand or limit your search?
CF10: Limit.

Related Articles:
I: Ok, great, that's right. So, find a really excellent article so that we can try out "related articles".
I: So what is "related articles" going to do for you?
CF10: Related Articles is going to give me everything that is specific to the text of this article.
I: Do you see this one?
CF10: Yes, it looks like it has green on it. We did not cover green.
I: Let's click into it and see what it means…
CF10: Oh it gives you the full text. And they are always green?

I: Well there is orange too, which you want to look for… is this a useful article for you?
CF10: It's a bit too heavy; it is going to go beyond what I need clinically.
I: Ok, well let's open it up anyways and see it, at least.
CF10: Oh there is more green here (She scrolls through it). Ok, let's do this one, can we look at it.
I: Sure, click the icon or the name, whatever you want.
CF10: (takes a few minutes to read). Again this is too technical for me.
I: Ok, so when you clicked on the related articles from your initial search it took you on a string you didn't want to go in… so it may not always serve you.
CF10: uh huh.
I: To see the free full text and how it works, click on the red button, scroll down, did you see this? This is the entire article right there.
CF10: Again this would be way too technical for what I need.
I: But do you see how easy it is to get to free articles?
CF10: It is one click away. (She reads more through the list. All the information from this list seems too technical for her).
I: Ok, let's go back.
CF10: How do I go back just click here? Oh no… what did I do?
I: It's ok, anything that you have done can be undone.
CF10: Yes that was my biggest fear, I am always afraid I am going to lose something or that something will blow up or something.
I: It's hard to delete something off the computer. They are designed to help you not delete. I am going to show you some more tools right now, but I basically I want to make clear that when you arrive at this site to find information you want to drill drown and down and down because there is so much information on this site. There are many ways to do this. One way is to place limits on.

Limits:
(We go to limits and click off many. She understands what and why we are clicking off.)
CF10: Oh yes, complimentary medicine.
I: (I explain Pubmed date versus Publication date).
CF10: Yes, I understand, but if we don't fill it in it'll still understand it …
I: Yah, it just ignores it. Ok and let's say you want items with abstracts. So do you see what these limits do?
CF10: They refine and limit the search.
I: Right. So what does this mean?
CF10: That the search will become specific to my needs.
I: Right- like Boolean is a limiter and 'Limits' is a limiter. OK right visceral pain and women again, let's try it.
CF10: Ok. Wow – one article.
I: From a complimentary medicine article, so it might have a lot value.
CF10: Yes, yes very good. So do we want to read it?
I: Sure… go ahead and take a look.
CF10: Ok, it's interesting. That's about all I can say. I am not that interested in this aspect of visceral pain.
I: Ok, so now I want you to look up anything you want, whatever way you want to. Note that the limits are still, so you can leave them or take them off, and I'll be here if you have a question.
CF10: (She looks for something and find nothing, and laughs).
I: Don't feel bad, may be you put too many limits on the subject. Remember, complimentary medicine is still on. If you were to try core clinical journals… sorry.
CF10: Oh yes I was going to do that.

(We find many articles)
CF10: This one looks interesting, and this one is really cool. (She looks through the list some more.) Ok, what would you like me to do? Go into Related articles?
I: What would you like to do? Remember, if you really like this article you can check it off and email it yourself, but you don't have to.
CF10: Ok, I think I'll do that. (She actually sent the article to text by accident and got very nervous).
I: (I explain the send to text function).
(She has a little trouble sending to email, but she practices a few times to get it.)
I: Do you think you would use the limits when you were searching?
CF10: Probably not every time. I think I would use the limits if I have a problem patient who comes at me with a Google paper, like if she was a 45 year-old female who found something and said, why don't I try this? But when I am browsing I wouldn't put the limits in.
I: And what about related articles?
CF10: Uhh, when I see the word Spinal there I certainly would go hunting. I would definitely go for related articles there.
I: And boolean terms? What did you think of that process?
CF10: Yes, that was very useful knowledge. Anne explained that to me before but she did it in the principle of MeSH. But I found like I don't think I can remember readily…
I: What the Mesh Stuff?
CF10: Yes.
I: Yes, MeSh is tough.
CF10: ok, don't confuse me…

History:
I: Ok, let me show you a couple of more things about Pubmed. Do you remember how I said it is hard to delete things? Well, history gives you exactly looked for at the different points in time. It is a record of your work. (I explain in detail to her the entire page).
CF10: Does it keep it forever?
I: You have 8 hours of inactivity. And you can combine your searches…
CF10: ohh…
I: What would happen if you combined your searches?
CF10: It would narrow it down even more.
I: Right. It even tracked your related article search. It's great because it is so easy to get lost in your searches.
CF10: That is great. (she plays around more with it, seems impressed).

Clipboard:
I: (I explain the function of clipboard – saving them while researching instead of sending to email. We select a bunch of articles, and we send them to the clipboard. They are random files. We go step by step. I explain the functions of it as well.)
CF10: The CMCC librarian provides me with an enormous amount of information now on evidence based practices because that was another one of my weak areas and that happened while I was away. So I put all her stuff into a special folder. Once a month I go in and see what's applicable and not applicable. I read them all. I went in yesterday and I think I must have deleted a bunch of them because they were not relevant. So, what happens when I want to keep a few of those permanently?
I: Good question, what should you do?
CF10: Can I call you?
I: Hmm
CF10: I would send it email? But that's not the right answer.

I: Why not? I think that's a great answer.
CF10: Yes, because then from my email I can send it my folder or I can print it.
I: Ok, so what would you do to send it to your email?
CF10: (She goes through the process… and sends them to herself … and without my help).
I: Great well done. Well, I think that's about it for today. Do you know the Pubmed URL?
CF10: I draw a blank on what URL is…
I: It basically means the location. And the url for pubmed is µwww.pubmed.com§.

Interview with CM11:
I: Ok, first, the object is to drill down. You want to narrow down your topic as much as possible and get as specific as possible.
CM11: Uh huh.

Boolean:
I: you don't necessarily want to be large- you want to be small. In order to do this, the first thing we are going to go through is a Boolean search. So let's say you want to look up 'effectiveness of chiropractic'. So you want to do effectiveness AND chiropractic. So you are basically limiting your topic this way because your topic in general is chiropractic but by putting the effectiveness in, then you are making it more specific. You are drilling.
CM11: So the AND forces them together?
I: Exactly, it forces them together –putting them together makes them more specific.
CM11: uh-huh.
I: So if you put effectiveness AND chiropractic AND government, you would get even fewer, but in a sense that is what you want. Ok, you want as few as possible. You can also do effectiveness AND chiropractic NOT massage therapy – you know like make it more specific that way. That's what called a Boolean search- I am sure you have done this before.
CM11: Well, government is not necessary. My problem is with this work is that I get a lot of people talking about this but the information is related to tiny little projects like a case study or something.
I: Yes.
CM11: Doesn't have great impact. So I am looking for big studies.
I: Well, you can limit by the type of studies that you want to get at. But that's a little further on in our study.
CM11: ok, ok. So effectiveness and chiropractic is ok then…
(We do the Boolean search).
I: And you know about the rules like if you put the * symbol after chiropractic…
CM11: You know, I don't- I sort of know the rules, but I don't really. So, what is the rule?
I: If you put the star after chiropractic, it is basically like truncating. And it'll then find anything with chiropractic in it – like, well what else is there?
CM11: Well, there could be chiropractor!
I: Exactly, so that's what that does.
CM11: So, it'll take the root?
I: Yes, exactly.
CM11: Actually, I didn't know that – I had seen it before, but I didn't know what it was. (he looks through results), is this the total amount on the list?1-2?
I: Well, let me take you through things first, and I'll show you all around the web page.

These here are the authors, in the blue. And when it is blue, you know that you can click on it. This is the title of the article, name of the journal, and then this is the Pubmed number.
CM11: This looks like a good one (referring to the article).
I: There is actually a lot about children and chiropractic…
CM11: Yeh, I just read today that our director of research, is also the editor of the chiropractic association's journal, has an article going into the journal of pediatrics about childhood chiropractic. And just this morning I looked over the article.
I: Wow, pretty impressive. We should get your journal linked up with Pubmed. But that's a whole other story…
CM11: Yes, he's trying to, but it's not that straightforward.
I: Yes, it takes a lot of time. But this is basically what you've got here. The author, the title. The name of the journal. And the reference number. Here you have a little sign. And I want you to notice what this is. This is an icon, and these lines within the icon indicate that there is an abstract that you can read. So, if there is no lines there, and let me scroll down to show you what it looks like, (We scroll down and find one with the orange and green labels, free full text in Pubmed.) oh, here is an interesting one, this icon indicates that is available online and open access within the Pubmed interface/database .
CM11: Oh Humphries (referring to the author of the article), I know him.
I: Oh, do you? So just note those differences in the icon.
CM11: Ok, so this one means that it is freely available?
I: Exactly. In fact you can click into it. See, it says free full text article. And it is available in Pubmed Central. In other words, you don't have to leave the Pubmed web site to go and get this article. Pubmed central is another database with full text that is associated with Pubmed. All you need to do is click on it to get to the article.
CM11: Click here?
I: Well, not yet. Have a look at this abstract and let me know whether it is even a useful article to you.
CM11: No. It should be but it isn't, its… now this is interesting. And I don't know how a Boolean search could control for this but quite often we have folks who publish things and they discover that such and such a diagnostic tool is good, or that more research is needed.
I: Oh. So they write something and the actual findings are not so great- are ambiguous.
CM11: They don't take us anywhere. This one here (He quotes from the paper) doesn't advance our agenda.
I: You might find that more with chiropractic and emerging medicine because it is an emerging field and is only now being written about in peer-reviewed journals.
CM11: Yah, yah.
I: Let's look at the next one by your buddy Humphries.
(he looks over the next Humphries article)
CM11: Again, no this will not be useful.
I: Do you think if we found an article that was "related to" this, it might be useful?
CM11: That's quite possible.
I: Ok, let's then try another little feature of this web site.
CM11: Ok, but I don't understand why there are two. (referring to the green and the orange bars on the icon).
I: Ok, well why don't we have to look at both… (I show him the difference: we click on the journal website button to get to the article, and then we go to the PMC web site. We

evaluate the differences- going over the added functionality in Pubmed central as compared to the other journal web site). So, just to be sure the one will take you to a journal web site, and the other will keep you in Pubmed …
CM11: Oh, ok. So for my purpose it sounds like the Pubmed option is more robust.
I: There are costs and benefits associated with both.
CM11: In my case I am not the end user; I am a conduit. Chances are the people I work with can understand it better and find it more easily than me (referring to the research- not really answering my question.) So it seems to me that Pubmed will serve as a meeting place for … you see I might write a letter to the minister of health, and he or she, it happens that there is a HE and SHE occupying these roles today, might read the letter and then click on the link to Pubmed, and that would make sense to them. I think Pubmed also is known outside.
I: You would be still be finding all your info in Pubmed. It's just a matter of getting at the Open access or free article in Pubmed. Would you prefer to stay in Pubmed or go to another web site to find it? Even if its one click away?
CM11: My feeling is that I would stay in Pubmed.

Abstract:
CM11: The other thing about my kind of work is that I am not working as an academic, I am working as an administrator and I am mostly interested in, when you ask me to look at an article, I would go immediately to what did they find to and then if that has meaning for me, then I would like to go into the article to see what they did. For a couple of reasons. One is no one ever does an abstract that is exactly right. No one who has ever done research has ever done an abstract that exactly covers the detail of what's done inside. So when you actually read the research paper you see more. Of course you see more, but you see more in different ways. So, you really do need to look inside. But if this doesn't give me an indication of what's inside the article then I don't look. What's interesting though I would sometimes read this conclusion, and find that it doesn't really help the chiropractors cause, but the article does…

I: So using the abstract to judge whether the article is useful isn't really the best way to determine this…
CM11: No, it's only marginally effective. There was a study done in the New England Journal of Medicine by a number of people, one of them was Ted Krauger, and they were studying chiropractic and its effectiveness for relieving asthma in children … they did discover by certain criteria that it didn't absolutely do it. But when you read the article you discover the children were healthier and coughed less. But you had to read the article to find out, because the criteria they established for the conclusions were of this less, Actually reading the quail[itative] stuff you realized they were better off. They didn't take drugs anymore.
I: So stuff is left out of the abstract that is really important.
CM11: In terms of that case they definitely were and that happens quite often. So the abstract is a good indicator for me but it is never enough, and it never provides that robust meaty quote that I need, because it sort of homogenizes it all. Abstracts are wonderful, they give you a start, but you have to read the damn thing before I know if there is actually stuff in there for me to use.
I: Very interesting. That's the type of stuff we are trying to figure out.
CM11: Well I also read Men's Health Magazine, and in MHM they have some pages

where they have snippets of highlights of research. They just take a little piece of an abstract and just give you the broad overview. But that's ok for that context. But if you actually want to impress people to make decisions about health care dollars in Canada you really need to know what its saying. So for my purposes I do need to have access to that stuff.
I: Ok, very interesting. Good commentary- good info. Let's try something else that's pretty cool. We are trying to get better information or more information.

Related Articles:
I: Now instead of doing a whole search over, we are going to use this article, and do you see that link (related articles link), and basically that pull up articles that relate to your initial one. So here you see the initial one, just in case you've forgotten, an then all of these afterwards were found, miraculously, pulled up another 19 articles that related to this initial one. So if you are starting with a good stepping point and you want more like it, then you hit related articles and it find more articles for you.

CM11: Now, you did send me …this article appeared before in what you sent me, and I noticed that when I clicked on this we got 20 or something, but originally we didn't have those 20. When I clicked on an article the related articles to that weren't necessarily all referenced in the original bunch.
I: err, I lost you… sorry.
CM11: Me too. Ok, let's go back.
I: Ok.
CM11: there are 20 items here. So these are related by Virtual search. Now you would think that if we clicked on this one, we would get all the same ones again… (asking why we don't get the same results as with our Boolean search – effectiveness and chiropractic).
I: Ahh, I see, no well, you see it searches the info in this abstract. It's not searching for effectiveness and chiropractic. So, effectiveness and chiropractic is found in all of these abstracts,
CM11: Yah…
I: But what Related Articles did, in a much more complicated way, is searched the metadata and the abstract and put together a list based on what was in this…
CM11: Oh, so its no longer controlled by the "effectiveness and chiropractic."
I: Right, exactly
CM11: Just articles related to this one... so you could have 400 related to this one.
I: Yah, well we only got 20, but you can search over and over again.
CM11: Ok, but that means that our related articles can be much better than this one, or totally different from what we are looking for. So, what would I do with those?
I: Well, you have to look through the list and let me know if it actually worked for you.
CM11: Ok, there's no way I can say take these articles and let me know if I can do anything with these.
I: Umm, I see what you mean, is there any other way of automatically filtering out the articles.
CM11: Yah, is there some sort of filtration thing, I mean, I would look through the list but …
I: No, the assumption would be that the initial Humphries article was so perfect that you wanted another 20 articles like it.

CM11: And then whatever they are, I would then look inside and see there was no other
I: Or the Humphries article was almost perfect but was missing particular dimensions. I am going to hit related articles so that may be the perfect article would come up. It is also good at finding open access articles so if you scroll down a little bit see there's another one that is open source. And they seem all to be from Biomed, or complementary and alternative medicine. Which may be good or may be not.
CM11: It's interesting how often we do get this kind of result. (Referring to research results within the article we were looking at). "the lack of acceptance reference standard contributing to the weak sensitivity findings". Means nothing. Its just we did all this work we can't tell you anything.
I: Right. Well, it does mean something.
CM11: its good for the academic world… but
I: Right. It doesn't help you make your case, for your point
CM11: Yah, it's just …

Send to:
I: Well, may be lets try some other things. So basically I want to make sure that instead of finding awesome information, I want to make sure you know what to
CM11: Right, how to do stuff…
I: So, let's pretend that Humphries is a good article, and you want to recall it in the future or send it to somebody. So click off this radio button right here, and then pull down that tab there. Then hit send to. Ok, what you might do is put a little note here that says something about the article- could be a note to yourself or to the person you are sending it to. "dear Me, xyz". Put your email address in there. And then hit mail. Ok, this pink ribbon here denotes that the email was sent to you.
CM11: Oh, I never used this facility.
I: Yeh- and this is all free by the way. I don't have any special rights or anything. This is available to you at your home, at your desk anywhere you are… so you don't need the librarian to help you find things. You can call them up and send them to yourself…
CM11: Well, I need her to send me the PDF of the article…
I: Right. Of course. Ok, now if you are at your desk, Hit send to text. Ok, now it has turned this information about the article into a text file. No images or anything, there is only text which has no formatting. You can save this on your desktop in a Pubmed file that you save all of your research in… the idea is that it strips it of all formatting and allows you to hold onto just the information very easily.
CM11: But when I have done that, I have saved sufficient information for me to find the article again.
I: Exactly. But you haven't actually saved the article.
CM11: No, what we often do when we come across an article that is useful we think chiropractic folks are interested in knowing about is put it in a reference library on our web site and if we can we'll have a hot link to something. Technically that's enough to have in a library. But if a person could click on something that's free and see everything themselves, that's better.
I: Yes, but this is more for you. Like, if you have a folder full of information about articles, and they are interesting to you, but you want just the information about it. Then this function is useful. It gets you to the information easily for future reference. Its not really hyperlinked or anything. It's not active. It's just the info about the article you liked.

CM11: I've got to look at them again for my own mind
I: Sure … if there's five of them- well let's go back to our list and try it again…
CM11: I just want to make sure what I did there …
I: Let's do something again- I promise it'll help you get it.
CM11: Ok.
I: Ok, please select 4 or 5 of the articles, now 'send to text'.
CM11: (Does it). Oh, that was easy. I really didn't know how to use it before I came in.
I: Yah, and there is more stuff too. You could actually send that to the clipboard – see the pink? It says four items were added to the clipboard. You can store 500 up there. So let's say you have been searching and searching, and periodically you kept on finding articles – instead of stopping every time and sending them to your email, you may save them your clipboard and thereby amalgamate them on the clipboard, then you would send them all to your email in box or to a text file.
CM11: They aren't alphabetized are they?
I: I don't think so… now you are asking too much! It does say sort here- so may be you can. So go to clipboard. So you can actually sort them alphabetically by author.
CM11: Or Pub date, or, but hey, why is it doing that? (Playing around).
I: I think you have to actually select them…
CM11: Ok, I am working in my clipboard now though… and I am sorting by, oh well let's see will anything change if I sort by author? Well yes it will
I: Ok, now hit display...
CM11: Oh – there you go. So, I selected sorted by author and then changed the display? (Trying to understand the process).
I: Well, you asked it to 'sort by author' and then you told it to 'sort by author' when you hit display.
CM11: (to himself) Ok, so I do this and then I change display, and
I: Uh huh, and then change display.
CM11: Wow! So you said up to 500?
I: Yup.
CM11: Over the course of the day I can sort it and then I have my list in order. Now I can take that, display list and send it to text?
I: Yes, try it select them all… oh, you don't even have to select them…
CM11: well they were there already…
I: That's true.
CM11: Ahh I think I am in love again!

History:
I: Another interesting thing is if you hit on history.
CM11: Where's history.
I: I'm not telling you!
CM11: oh my god, I have got to read it too?
I: I'm sorry.
CM11: history, history, ahh found it, history! (Reads over instructions.)
I: Ok, should I explain it to you?
CM11: Yes…
I: Basically, this is what you have done in Pubmed. So our first search was number 1: "effectiveness and chiropractic". Them we chose the "Humphries" article, then from

there we did related articles, and then the clipboard thing. Now if we did search chiropractic as one search, and then we did search- here lets try something. So just do a regular chiropractic search.
(He types it in)
I: Ok, so here you have 3124 results. Now let's do another search. "Spinal cord".
(He types it in)
I: Ok, we get 9000. Now go to history and now we are going to combine our last two searches. Number 10 and number 11. Click on number 10, hit AND and number 11 and hit go. Ok, before you look, I want to know something. Do you think you will get more or less articles now?
CM11: Well, first I thought more, but I peeked…
I: When you combine the searches you are making the topic more specific.
CM11: Yes, of course, we said that already.
I: Yes, it is tough to get at first because you think, 'combining something= more' but in this case it is 'combining something= less'. So you specify, and that's what you want to do.
CM11: Yes, you combine and then you are looking at overlapping sets. This is where the sets intersect.
I: Yes. It's like a Ven Diagram. See this first icon? What do you think it means?
CM11: It means that's what I am seeing. What do I see? (i.e. doesn't know).
I: It means that there is no abstract available. So if you were to click on this, you wouldn't have an abstract. It would just be ...
CM11: That's an awful lot of what I get. Generally, I get this when I use Pubmed, and I just throw it away because it is just too much work finding everything, so I just don't bother. I need an abstract to get me started.
I: The way [the librarian] teaches her students to use Pubmed is by using history. Instead of asking them to figure out the Boolean, she asks them to do big searches and then combine them like we just did. SO, click over there: you see it gives you all of the Boolean options.
CM11: I have never heard of that. It is like brainstorming. Its like, you think of all the things that could be, and wow… its wonderful because you don't have to have a tightly organized search pattern. It could be this and this and this could effect and blah blah blah…. The difficulty is the prejudice you take to your search question. May be you missed something because you describe it badly.
I: You could do many combinations using history. You could do search number 12 and 13…
CM11: Let me do that... I'll do 12 and 1. So to do that I add this and this … (needs some help). It's not intuitive to me. I struggle with it… (referring to figuring out how to use the website/computer.)
(We accidentally deleted number 11)
CM11: It sounds like it needed us not to get rid of 11 if they wanted us to combine using number 12.
I: Yes, you need 11. See you are getting the hang of this.

Limits:
I: Ok, let's do something totally different now. We'll do a regular search. Put chiropractic into the search bar… but do not hit go. I want you to click on limits. (I want him to find it by himself).

CM11: Ok – this is a little test now.
I: Yes (He finds it). You might find this interesting and useful because it is another way of limiting your search without having to do Boolean. No brain teasing logical rules. Instead of limiting through keywords and subject matter, you will be limiting through the meta information. Meta information is information about information – what all journals have in common: a journal name, an author, title of the paper, an abstract. Information that is common to all journal articles. Here we see all of the fields of metadata. We have languages- and we could limit to English. Humans or animals: people being the subject matter of the paper, say as opposed to rats…
CM11: It's interesting you mention that because lots in our profession have done lots of stuff on animals. But it doesn't impress anyone outside of our profession. They don't care how well cats and dogs do …
I: ok, well what if you are only interested in men? Women? Gender…
CM11: ok, that's never really an issue usually, but …
I: Ok, ages.
CM11: (he chooses one)
I: Subsets is an interesting one… so these are all different databases full of in formation. So you can limit by database …
CM11: If I don't pick subsets then I get stuff from lots of different areas (as opposed to limiting by subsets). Right?
I: Right.
CM11: Pubmed samples are pretty big and reliable when it comes to - you get lots of stuff…
I: You can actually cut out the complementary medicine journals. So, if you already know that literature, or you want to search outside of that literature, you can limit by subset… Publication types…
CM11: So now we are getting into types of research- look at this – (reads options out loud to himself). So if you look at Sackit (?) and his different levels of high qualities you would find meta-analysis at the top and RCTs and then some of the other…
I: A lot of the chiropractic students I have been working with like the RCTs.
CM11: Yes, well I wrote an article about people being so married to RCTs that they can't think without them. But it's more impressive, and meta analysis is even more impressive … and I am stuck with an incredible conundrum in my profession writing about it, simply because it is more or less impossible to do an RCT of chiropractic since you have to do a sham adjustment. You can't do a sham adjustment – because people would know. You can't blind a guy who is almost not doing it. You can't blind a practitioner. Its more or less impossible to blind the patient so any effect of randomization is nonsense. So it makes it real hard to do a meta-analysis of RCTs.
I: You can't give them a placebo.
CM11: No, some people try. But it's ludicrous.
(We found no RCT or meta-analyses).
CM11: You have to laugh at some of this stuff. This is the kind of thing that may be useful inside (i.e in the text), but is totally useless as it stands. It doesn't tell you anything about anything, it's too big.
I: Do you think it would be a good article?
CM11: Might be, but in order for me to know I would have to look here … and go beyond the first paragraph, and at the whole text. This stuff needs the light of day.
I: Well, this one is free… can you scroll down the list and see anything you want?

CM11: well this is the one that is fabulous, it came out in October which had 700 000 people… remember I mentioned it. This one is potent stuff because they actually give number s of cost per person. I did some calculations base don this stuff and we would be saving billions per year.
I: Well, you should get this study sent to you.
CM11: Yes I have it and we use it over and over again. We have a bunch of stuff and we really use it. (Continues looking through the list). Where in the world are these published?
I: Great question- I was just going to go over it. Choose an article you are interested in-not the open access one.

Linkout:
I: Hit links, and choose Linkout… this is basically a map to the actual article. It gives you information to where the article actually is. So, EBSCO, OVID and Proquest all have the full text of the article published.
CM11: Oh, right. (very excited)...Well, some medical journals protect their stuff for a month or 6 months.
I: Right- that's true. So you want to go Linkout and it will give you basically-
CM11: Linkout- I can't get my mind around why it would be called Linkout?
I: Oh, click on libraries … and then on the letter G- or any other letter. So here you have a list of the libraries and organizations that have this article. Some have it print. Some have it electronic, and some have it in both forms.
CM11: what about us? Are we called U of T or Toronto?
I: How about checking if CMCC has it?
CM11: Oh they'll have it for sure…
I: I'm not sure they have it… may be you have it through UofT?
CM11: I'm surprised CMCC is not listed.
I: It's only the libraries that have it that are listed.
CM11: But they get the Annals of Internal Medicine…
I: They seem not to have it here, unless CMCC is not listed with Pubmed …
CM11: Interesting. This has been very useful because – this has been magnificent. A couple of the features of Pubmed, I didn't have time, or was too lazy or stupid or whatever- I didn't use it.
I: Some times people are afraid because it is so complicated…it takes sitting down with someone and have them go through it with you…
CM11: Well, I think that's the only way to do it. I mean you can't write a book about it. You have to fiddle with it and do it. I should've dragged some of the folks form the office down…. we do want to know the literature. But we want to know what Annals has to say like once in 4 years, and we aren't going to spend the money on a membership for that…
I: Ok, let's show you some other stuff. Do you this yellow thing here? It shows you what is clicked on in terms of your limits. ALL, Child, 0-18, English, Human. If you unclick Limits here, this goes away. Now, lets just do a regular search for what you want to do.
CM11: ok, umm, how about chiropractic and effectiveness"
I: Again? We already did that one. Let's choose something different.
CM11: What about comparing chiropractic to other forms of treatment for back problems.
I: What other forms of treatment are there?

CM11: Well, there is taking drugs.
I: Ok.
CM11: It is often compared to anti-inflammatory drugs.
I: Ok, how about we try, treatments for back pain.
CM11: Ok, treatment back pain. Should I add an AND to it?
I: We can try it both ways. We could even do it the [reference librarian] way.
CM11: We get 13, 000. That's a lot.
I: Ok, let's try adding the 'AND' and see if it limits it. So treatment AND back AND pain.
CM11: A few.
I: Hmm, we need to refine some more. I wonder how we should look up compare. Let's first look at your results and see what is going … may be we could add "not acupuncture" … what else wouldn't you want.
CM11: Actually, I would want NOT complementary and alternative medicine because I would like to compare chiropractic to ordinary medicine and medical procedures.
I: But I am afraid that chiropractic would be complementary. So, you cannot eliminate it.
CM11: Yes, but it's both.
I: Yes, it fits in between the two…
CM11: Yes…
I: Well, lets start by saying NOT acupuncture and see…
CM11: I wonder why, did I spell it wrong or something?
I: Hmm, looks like there are two "C"s. Let's also go to limits, and then subsets and then core clinical journals. And now hit go. (We get fewer results). Ok, that's better.
CM11: It's actually better by about ten times.
I: Ok, well there you go.
CM11: But there's a lot of flab in there just the same. Oh, you know why? Because back pain can be a function of any source. We should be looking at musculo-skeletal sources of back pain. So one way of doing this would be to add AND musculo-skeletal [figuring out a search strategy for himself. Adds musculo-skeletal]. Oh, it doesn't help…
I: You know why? Its back and pain…that's our problem. May be take out the AND in between back pain. Oh we get more …hmm, what is back pain. Let's go to the Mesh database and search back pain. So here they call it…
CM11: What's Mesh mean?
I: Mesh means …it's a controlled vocabulary database.
CM11: Ok, like when you say back pain you are meaning this…
I: Yes, like what the literature calls it, so we can try 'low back pain' (Suggested by Mesh) if we want- that'll get us more specific.
CM11: What happens if I click on this.
I: Go for it…
CM11: Oh, is this the way to delimit terms- (looking at the Mesh list).
(explores)
CM11: So if I were to click these sub-menus for instance, if I am interested in comparing chiropractic efficacy for back pain, and I want to compare that to non-steroidal anti-inflammatory drugs, for instance, do I have to have both things that I am comparing to in the headings? Say I want to compare back pain solutions, chiropractic solutions, surgical, I would have to have surgical in there, wouldn't I?
I: Yes! Ok, so if we are going to try that we got to go back into the Pubmed database, see it says here we are in the Mesh database?
CM11: Ok. So I am in Pubmed now.

I: Ok, now search back pain …
CM11: This is making some sense if it works. And then I've got to add chiropractic AND surgery. (He does the search). I did come up with one study- the one that I love. But that's probably the only one there is.
I: May you should conduct your own studies ...
CM11: Well, what it means is that our search criteria worked because it came up with the study I was looking for.
I: So the Mesh worked…
CM11: The Mesh worked…so what I did was go to mesh and
I: So now you would start over. Or go to history for a second. Your most recent query within the Mesh database was low back pain. Now if you can, go back to Pubmed (We go back) and it doesn't show us that the Mesh terms were applied to the search…
CM11: But it did show in the Mesh history that it did that work…
I: Hmm, I need to look into that issue why the Mesh history and the Pubmed history were separate.
CM11: But it does show that there were two searches in the Pubmed history that were not named. Did you see this?
I: Hey- you are right.
CM11: So, Mesh is in there. But it wasn't part of this…
I: Click on number 30 for a second- we'll see what happens… and scroll down to details – yup there you go (we see our Mesh searches there).
CM11: So I guess whatever you do over on that side in that way will be buried under Pubmed's heading number 30 (it was our 30th search as recorded in the history tab). And the number is kept there so we know something is missing – showing we did like a sub-search. Redefining the terms.
I: There you go.
CM11: Well, that's very nice.
I: That's it, we did everything. So that's talk about a couple things. What did you find to be the most useful?
CM11: Umm, well, I think you do a good job at explaining things and coming to a quick so that's very useful. I think that finding my way around the site and understanding its power and its variety is everything. So that's extremely useful.
I: Do you have a favourite tool? Like, related articles? Or history or linkout?
CM11: I think my favourite tool would be searching with AND (Boolean) and history together.
I: Come to think of it there is one more thing that I wanted to do: take that study that you like, and hit related articles form it. So go to number 30 (marks the number of the study that he liked that we found) – and go to results, and now click on related articles.
CM11: Ok, now, that's got to be useful, what have got here? 1-20 20 of 110.
I: One year follow up comparison of the cost of effectiveness of chiropractic and physical therapy as primary management for back pain. Journal of Spine- or Spine journal. What's that? (We read the abstract). Ok, I would suggest- these are pretty good.
CM11: I think so too. And I don't remember seeing this fellow. I remember seeing the others, but I don't remember seeing this one.
I: So what would you do next? Because I need a coffee and you want to go home?
CM11: Ahh, well we can get a cup of tea … (he laughs) is that what you meant?
I: No, I meant what's the next step?
CM11: I will try to see if there is any indication here of … (then mumbles to himself)...

reading through the list for the results.) Hmm, that's not a result of any interest. Ok, you are testing me aren't you?
I: Yes, a little bit…
CM11: So now I have to remember how to find out whether ... let's see I have to take that.
I: Let's say you do want to sort through all 110 of these but you don't want to do it right now…
CM11: Oh! Well, I can send them to text
I: Well done- or even better send them to your …
CM11: Email!
I: There you go! But first you have to select them.
You could do this two ways: you could select all 110, and email them to yourself or, you could email just the titles- as opposed to all of the information related to them.
CM11: I didn't know you could email just the titles.
I: Oh, yes, you see where it says summary?
CM11: Yes.
I: If you just wanted to email the citation to yourself. Click on citation, and it would give you the citation - which is the author and the title and the volume.
CM11: Ok so I would send to text?
I: Send to email actually. It looks like you should really be sending the summary- not the citation. I was wrong.
CM11: I had forgotten one other thing and that is how to find out whether I can get this full article or not. You taught me, but I've forgotten. So I like this article, and I want to find out if it's available…
I: Go back to the list for a second, now scroll down the list. There, what's the difference between the icon at number 9 and the icon at number 10?
CM11: Oh, ok, this one is available.
I: Now go to Linkout, Then to links.
CM11: I got to create some sort of neumonic for the brain to try to remember that. So that's a critical feature for me to know …
I: Linkout.
CM11: The Linkout because I have to call (the reference librarian), and she had to give me that.
I: Now, if you don't send yourself the list, but you want to find it again, you could use the starting point of your favourite article, and hit the related articles button. And you would again pull up the list here.
CM11: And History saves for a day? Or eight hours of inactivity…
I: Right, so if you take a nap then make sure to wake up before those hours are up.
CM11: this is wonderful stuff… I really like the idea of lots of [chiropractic] students learning this because I like the idea that they are engaged in research. Interestingly, I called up the VP of the CMCC and talked to him about our organization getting memberships to the CMCC so we could access their library. And he was into it. And I spoke to him yesterday and he said it was ok for us to get membership so we can access EBSCO and the others. But it was enough of a need for us that we were scrambling around even with {the reference librarian) helping us. We really wanted to get at the information. And our social issue, our membership expects us to know things instantly if they are in the public domain. They say: why don't you know? Everyone else knows…
I: Yes, knowing things is invaluable to day.

CM11: This is lovely – thank you!
I: Thank you- you did great!

<u>Interview with CF12</u>
I: You have heard of Pubmed before?
CF12: Yes, I have heard of Pubmed
I: So what have you used it for?
CF12: I don't know that I have sued it before. I know I have heard of it, and I think I've used it, but I haven't intentionally used it.
I: Ok. And so long have you been a chiropractor?
CF12: Since 2001, 5 yrs.
I: And you went to CMCC?
CF12: Yup.
I: And what did you do before that? Do you have a BA?
CF12: I have a B sc. from McGill university.
I: What we are going to do is I am going to teach you how to use Pubmed, which is a free online index. In it you can research anything you want to in relation to chiropractic, or for that matter medical sciences… any type of scientific knowledge that you are looking for. And the thing that is good about it is that a lot of the information that you will find in here is now becoming open access. And that refers to it being freely available online. So you'll see when we do searches you'll see we can access some of the full texts of the articles … so what do you want to search for first?
CF12: umm, I would look up strokes and chiropractic.
I: Ok, let's go for it. So this is the search field so just enter it… and hit go. So here we have the search list. This is a citation, here is the author, the name of the article, journal, year, and so forth.
CF12: Yes…
I: So this is the icon and it shows that there is an abstract available. So let's click inot the article. Can you tell me if you would be interested in reading this paper, and what it would do for you?
CF12: (reads) I'm not sure… yeh it would be interesting to me.
I: do you ever research chiropractic for your own professional practice?
CF12: I do in relation to patients. So I'll have something come up and I'll look it up.
I: And where do you look it up?
CF12: I often would use my own textbooks, things like that. I would Google it. I have gone to Medline in the past but then been frustrated that I cannot get into papers.
I: Ok, so, Google and Medline. Any subscriptions to chiropractic journals?
CF12: I get, the journal of the Canadian Chiropractic Association, the JCCC.
I: and are you a member of the association?
CF12: I am a member of the CCA.
I: ok, so this article is available at BMJ.com, which is sometimes free and sometimes not. Is this what you are looking for?
CF12: I would be interested in larger studies actually.
I: ok, one way to do that would be to create some sort of search strategy is to hit related article… if you like this article, it'll help find articles more articles like this one. So notice, there is an article here that has a green bar, and that signifies that you can access the article for free online. So click into that one.
CF12: ok.
I: And here it is. Click on that button. It takes you to another web site… but what's the problem?
CF12: It's in Spanish!
I: Ok, so close out of it. And then just read over the abstract to see if 'related articles' was

useful.
CF12: The translation is pretty rough … like osteo-muscular pathology.is not a phrase anyone would use but (time passes as she reads). Yes, I would've wanted to read it.
I: So here again you have another case report. So, if you are looking for something bigger it is a bit of a problem. A lot of articles in chiropractic tend to be case studies as opposed to large clinical studies. But we can look for larger ones by limiting and strategizing better about how we search. Go back to the search list. I also just want you to scroll through and have a look to see if there are any other ones you are interested in.
CF12: (looks through the list and chooses one to look at). It's a retrospective review of the cases. (Reads some more).
I: So lets look at a couple of more things at the interface…. Whenever something is linked in blue, its links up to something else. If you really like these guys (the authors), you can technically click on the links and get more articles that they have written. If you feel like they are really experts.
CF12: So I have heard of him before. I would think it would be interesting, I would be interesting to know what his perspective was on other things. So can I click him?
I: Sure…some of them are just authors, and usually you see here they don't have abstracts in them because you wouldn't summarize an editorial.
CF12: but this relates back to this first article, so, like this is just an abstract, right?
I: So I'll show you where to get the article. Here is the publication type – its review…
CF12: I find a review is really helpful for me because I don't need to, a lot of what I would research is not groundbreaking its not like a new medicine that's come out where I need to know the newest thing on what on an adjustment does, and I don't need to read each study which says the statistics, so a review helps me…
I: So for your purpose as a practicing professional this is really good because it gives you access to what is going on without being too think and clinical.
CF12: Yup.

Linkout:
I: So one of the problems is that this is not available free, but you can get the full text if you want to. I mean, its available online, but not for free. If you did want to get it, you would hit links, and another menu pops up, and you'll hit linkout.
CF12: I would have no idea what this was if you wouldn't be here to tell me about it.
I: yes, it could be complicated.
CF12: Oh, we have to sign in…
I: Yes … but, just notice that a lot of these articles now have similar functions to Pubmed, so when you are on their web sites, you can find related articles (similar articles), the abstract is there, etc... and it even links up to this Highwire Press database. Ok so close out of it and go back a few times. So we have 68, (referring to the list now-68), and that's a good thing that we have fewer because we are trying to whittle the list down so you don't have to go through thousands of articles.
CF12: ok.

Boolean:
I: Before we totally close out of it, I want to show you a couple of things. I want to teach you how to do a more strategic search by showing you how to use Boolean. Do you know what Boolean is?
CF12: I might have heard of it.
I: Ok, it uses AND, OR, or NOT to try to specify what you are trying to get at. So you were interested in strokes and chiropractic. By Putting the AND word in you are putting them together – like in a Ven diagram. So put it in strokes and chiropractic and we'll see if it works better. Oh, one more thing the NOT operator works to exclude. So if you don't

want something then you would say x AND y NOT z… if that makes sense.
CF12: Ok, umm, I don't know what I would take out, I think its pretty inclusive … but we can do another search…
I: Yes, sure another search…
CF12: Do you want me to be very chiropractic specific?
I: Sure, it's for you actually, you are going to send this to yourself. Ok, great, so look at the search list and tell me if you like the articles.
CF12: Ok, I would probably read this first one here. I mean it is a little bit specific. I would probably read it, and this one I would find really interesting, from JMPT.

Send to text, email, clipboard:
I: What I want you to do is select one, and select two, and see it says send to. Click on the tab. And then just scroll down to email, and we'll just send it to your email. You can put what ever you want into that little field. Notice how it says that you can choose what format you want for it? So sometimes when you send it as Html it is a little bit bulky for your in-box so you can send it as text.
CF12: Ok, do you want me to send it to an email I can get from here?
I: Well, do you want to see it? We don't need to access it right now but in the future.
CF12: (She sends the docs to herself)
I: Ok and then you hit mail.
CF12: Ok.
I: Ok so the pink ribbon up here tells you that you have been able to do it. Ok so select 2 again, and we'll do another way of sorting and organizing which is another big part of using this. Hit clipboard, actually, go back for a sec. Hit 'send to', and see it says clipboard. Ok so it is sending it to your clipboard.
CF12: Ok, so it is putting it aside for you?
I: Yes so if you hit clipboard, these are the two that you just saved …
CF12: So you can look up tons of searches and put them all in your clipboard, and then you go back and then they are all there, so you don't have to write them down or bookmark them or try to find them again.
I: Or emailing them to yourself…
I: Right
CF12: Hmmm
I: This way you don't have to send a bunch through your email and wade through tem afterwards. You can return to clipboard and go through them before you send them, so you can look at them and say oh this one isn't really for me, I don't know what I was thinking… oh but this one is god I'd like to save them as a text file to computer so I have them, which is the next thing we are looking at. So select this one (a paper) and then select send to, and then text.
CF12: Ok
I: Ok, so notice what you see here is just the text
CF12: Right
I: And so it is a little easier to save these, you can copy and paste these to a document of citations and have them forever, save them on your computer. Ok, now click into this one because it is interesting..
CF12: Here?
I: Click into the title.
CF12: What is the difference between clicking into the title?
I: Nothing actually, they are the same thing there is always different point of access on a web page to the same place.
CF12: Ok.

Free full text, Pubmed Central:
I: This one is interesting because it is a free full text symbolized by the green bar, and also the orange bar shows us it is free in Pubmed central. Pubmed central is free. Basically if you find articles that are available in Pubmed Central, you can access them right away no problem without even leaving the Pubmed environment. So here is an article you can have access to and read. This is the abstract, and then if you click this button, we get to Pubmed Central: you can get it in PDF or HTML- and this is it basically.
CF12: Very cool.
I: So if you do want to download this is the PDF right here. Go all the way down and have a look at the references. So here are more references that might also be able to help you out. Some carry full text, and so you can do more research by looking here.
CF12: And then when it says Pubmed, can I click it?
I: Yah go for it.
CF12: This takes you to the abstract…
I: Yes.
CF12: So can I get these articles?
I: If they are free full text, which would be indicated in the citation, then you can get it. If it is full text online, it is the same deal- you would have to pay for it or find a library that could get it for you.
CF12: Ok.
I: Actually, this raises another interesting question. Do you know what the difference is between an index database and a journal database?
CF12: Would an index get them from multiple sources and a journal is just everything published in that journal. Is that right?
I: It's close. An index is basically a database of all the information about the journals that exists. So if you ever want to find an article you wouldn't really look it up in a journal web site, unless you knew about the article already…
CF12: Oh but an index is still particular to a journal?
I: Umm, an index is a database of information about journal articles. So when you want to look up a subject, you would say, I need to go to Pubmed the index to find articles about the subject- so if an article is in the index it would be really hard to find it. Yu wouldn't really be able to know about it, unless you were browsing through the journal…
CF12: Ok,
I: Yah, you probably knew that already, but its kind of hard to articulate it…
CF12: Ok, with this one, (one that was in the references) didn't we think that we would get the whole article? This is just the abstract…
I: Well, it is available online, it just isn't available for free. So, it'll cost you 30 bucks but you get it online. But basically the green ones and the orange are free. And if you wanted to you could try doing all of your research in Pubmed Central, to get constant access. Does that make sense?
CF12: Yah, ok, so can I do a search that I did today on google?
I: Yah, do it in Pubmed central and see if anything comes up.
CF12: It's slightly different- I mean it is acupuncture related.
I: It's fine…
(Does another search)
CF12: Should I do a Boolean search?
I: Yes please!
CF12: So these are all the full articles.
I: Yah, I am pretty sure. In fact click on it just to be sure and have a look.
CF12: Ok, I have to go to the publisher's web site. Ok, this isn't really clear though, it's more of a paper than a research study. Ok, this would be interesting.
I: Do want that one? You could try emailing it to yourself… do you remember how?

CF12: Ok, send to, email… (She mumbles to herself and does it on her own).
I: Well done! Ok, before you close out, I just want to show you one thing. In order to get to Pubmed you just go µwww.pubmed.com§, but then it takes you to this very complicated link. Pubmed consists of various databases, so if you pull this down, you'll see all of the different databases it is divided into. There is one specific to proteins, very specific scientific stuff in here. You were in PMC, and you want to go to Pubmed – so go back and select pubmed. So, sometimes you get disoriented so you would just look here to see where you are and get back there…
CF12: so I could do a search in Pubmed, and there were 4 that came up last time (in PMC) and may be more would come up – there's 13 this time.
I: right except that they are not all full text. But here some of them are but they link to a publisher's web site, they aren't in PMC.
CF12: Oh, ok.
I: Feel free to put these on your clipboard or email them to yourself.
CF12: can I click on this one?
I: Sure, anything you want…
CF12: I want all English. Oh this is exactly what I want, this is good. (Takes about 5 minutes to look through the result). This is great.
I: Well, choose where you want to send it.
CF12: Ok, so I can do it from here (from the search list). This is really nice- I mean I had no idea about this.

Books did not work – (possibly disabled at this point)
I: Great. Ok, so there's another thing: let's say you are really interested in Bell's Palsy… .but you need a book on it. I want you to click on LINKS to the side over there. And then hit books.
CF12: (she scans down to look at the MeSh terms) I don't understand, what is this? Is this everything with the key words?
I: What mesh terms are basically are synonyms. Or a list of how the research literature might refer to your topic. Say you are doing search on acupuncture or acupuncture therapy, but other people refer to it in a different way in the papers. So the Mesh database, and I can show you how to get there to look things up, provides you with other ways of searching for your topic. I have commonly used the examples of back pain when talking about this to chiropractors. People weren't being specific enough in their searches so we looked in the MeSH database and got more ways to find back pain: first instance looking up low back pain. So we can look up stuff in the Mesh database but I was trying to do something else which was to ... but to didn't work for some reason. Ok, go to links again, hmm I don't know… anyways

Limits:
I: I've got something else to show you anyways. This one is actually quite good for you because you have already experienced getting a Spanish article... so here you can choose to limit according to the meta –data of the information. So, for languages, you would select English. Let's say you wanted articles not about rats but about human beings, you would choose humans- women or men. And here they have divided this up into core clinical journals. So let's say you wanted to find out more about acupuncture, but you aren't that interested in what the alternative journals have to say but you want to see other perspectives…
CF12: Right because that's valid too because sometimes I don't want to know like a really alternative journal says, I want to read the larger stuff.
I: Right, so here's where you can choose what type of article you want, we got a lot of reviews and case studies. If you just want to find original research that's what you would

click.
CF12: (chooses some limits).
I: Date allows for the most recent research.
CF12: I could use stuff from like the past 5 years.
I: Ok and you could choose articles with abstracts…
CF12: So that would mean…
I: You would get articles only that only have abstracts associated with them. So this is one way to make things more specific.
(we get no hits!)
I: Yah, we put a lot of limits on… if it too many its limit overload! Ok, lets take some off.
CF12: Ok, core clinical journals, English and 5 years.
(still no hits).
I: Hmm, we only got 4 articles the last time when we put no articles on, for this search…
CF12: Oh yes, that must be it.
I: Ok, well I just want you to know if you ever do find something using limits, there is this yellow band up at the top that indicates what limits you have on.
CF12: Ok.
I: Let's try looking up anything else.
CF12: Ok, so do I redo here or do I reset my limits?
I: Good question- the limits stay on because they are checked.
CF12: Ok, so I can do, …oh I didn't do a Boolean.
I: It's ok. Oh interesting (We get nothing again), go back to limits…
CF12: What's going on here? I don't have any other limits…
I: Ok, unclick core complementary journals and let's try the search.
(we get 34 articles)
CF12: Midwifery, midwifery, they are all, none of them are core journals..
(We look at one of them)
CF12: This is something I would be interested in.. I can't believe there are no core journal articles about chiropractic and pregnancy…
I: Well there is JMPT and manipulative physiotherapy…
CF12: I don't know if that's considered core…
I: Ok, so what are your thoughts?
CF12: Hmm, thoughts are I had no idea this is way more effective than just Googling.
I: definitely
CF12: Yes, and I can't believe there wasn't anything in the core clinical journals. I mean chiropractic and pregnancy is so effective… I see so much of it, and I can't believe. There wasn't even anything in the chiropractic journals about it.

History:
[The recording was very poor for this part of the conversation.]

Mesh:
I: Ok, so here is your mesh database. Let's look up acupuncture and see how the literature might refer to acupuncture.
CF12: Acupuncture points, therapy, and ok…
I: Ok, so if you click these off, you can use these in your search to help you find specifically how acupuncture is referred to in the lit.
CF12: Ok…
I: ok, so the search box will send it to there (search field) so
CF12: Oh I see, so its basically saying that what she means by acupuncture is dah dah dah.. ok, so what was I looking for? Acupuncture and Bells Palsy.
I: Right. (We get nothing).

CF12: Hmm, I could see using this like a dictionary- so oh, because then at least it would tell you, look up acupuncture therapy ... right.
I: So let's say you like this article and you want this. Hit links, and then Linkout. This is where you try to find the article. You might have to pay.
CF12: So I click on either one? Whoever I want to give my money to.
I: Ok, so we go to their web site
CF12: Actually I was interested in the seasonal variations.
I: So scroll down and hit article full text. The other thing you could do is hit libraries
CF12: Oh you can find out what it is and get it. Make a day of it. It seems really inefficient to have it by library. Why not by city or something. Oh, look McGill is here… What is the thing to find it again? Linkout, and libraries. I think that's neat.
I: Ok, I think we have covered everything.
CF12: Like literally today I was looking this stuff up and like, I was Googling with my acupuncturist. It was taking us to acupuncture.com and it was really inefficient.
I: Great. Will you use it in the future?
CF12: Yah, we were going to work on this thing together so, yes, definitely.
I: Excellent. Can you tell me what your favourite tool was?
CF12: Favourite tool, what do you mean?
I: What will you use the most?
CF12: Umm, I'm not sure. I liked the Mesh because I don't know how to look for things – like acupuncture. I liked seeing how to look it up.
I: ok, great, thanks.

Interview with CM13
I: Ok, so these are the articles I found for you just by doing a simple search of Pubmed.
CM13: Ok, so this is on child obesity – in Canada too?
I: Some are, some aren't but we'll look at all three (We look at one of the articles). Ok, so now you are outside of Pubmed.
CM13: Oh, I see we are in the journal right now…
I: Exactly and this article happens to be free online. So how does it look?
CM13: It looked ok.
I: Right. So, to get to Pubmed go to www. Pubmed.com (I explain the homepage to him, multiple points of entry, different databases, etc… the notion of limiting the scope of the search, refining the search to get what you want, we put in a general search for childhood obesity. I explain what a citation is, and the various elements).
CM13: So the whole article will be there in Pubmed?
I: No. this is the information about the abstract, some articles are in there. Some citations don't even have abstracts. You need to look at the icon to see, do you see that?
CM13: Yes. So that would be the whole journal is there?
I: Well, with this one (an OA article) its not that the whole journal is there
CM13: Oh, I mean the whole article is there.
I: Exactly, there is a link to the journal article.
CM13: So all the rest its just the abstract or no abstract at all. So what the heck is this? What's the use of that? (referring to one with no abstract – probably an editorial or something)
I: So, do you feel you need an abstract then?
CM13: Well it's telling me the same thing that's right here- I like abstracts. Because it'll tell you, you can make your mind up about how the research is done and stuff like that. Like it'll give you more information – like this is not telling me if it's a good journal or anything. If its going to be reliable, like how they are going to conduct the research. Like

give me a little hint or something.
I: Ok, a lot of people do like an abstract.
CM13: No, I'm not saying I wouldn't read it. Like if I had an article in front of me, I wouldn't necessarily write it off. But the chance of me reading it is a lot higher if it had one.
I: Ok. So do you see any articles that you like?
CM13: ok, this might be good. So I should just pick and we go a step further?
I: Yes, if you don't mind.
CM13: Ok. (he reads for a while). Ok, I'll just go with the first one.

Related Articles:
I: (I explain the tool to him).
CM13: But some of these articles may be present in the initial search, right?
I: yes, that's true,
CM13: Ok I see.
I: So will you look through the list and let me know what you think of the results?
CM13: Ok (time passes). It looks like good results. It's not exactly what I might have looked for but some of these articles look ok. It would be good to do this to find things I might not be looking for.
I: Ok, well, looking through the titles, is this what you had in mind in terms of your topic.
CM13: Well, not exactly, but they still look interesting.

Boolean:
I: And there is another way of doing a search to make it more specific, it is called Boolean.
CM13: Right, I have heard of it. It is when you use and, equals…
I: Ok, so we put child obesity AND Canadian or Canada*, do you know what that does? (We type it in)
CM13: Yes, it fills in all of the, like Canadian…
I: Yes, right. Good. So you got 189 articles. This list is a little different than the related articles search since it is a little more specific.
CM13: So does it pull up like the term itself exactly or does it pull up child AND obesity AND Canada.
I: Yes, not the exact phrase. You could do that if you used quotations.
CM13: So that would narrow it down a little more.
I: Should we try it?
CM13: Ok, and use truncation in it as well.
I: Oh, it seems to be the same- hmm, may be I was wrong…
CM13: That's ok.

History:
CM13: Ok, so this is al of the searches that I did
I: You can do the Boolean strategy using history as opposed to creating a search string
CM13: So I can choose 7 and 9 and put them together.
I: right. So let's try that. (We combine some searches).
CM13: Four articles. So this is similar to the EBSCO, eh? The one we use at school here. I'm not an EBSCO expert myself but I saw Anne about a tutorial and she gave me one a couple of weeks ago.

I: right but this is available any time
CM13: Oh yeh, with EBSCO you have to get passwords and stuff. Which is very confusing. So can save these searches?
I: Well once you have found articles that you love, you want to get the information to you so you can follow up on it. So, first select the article. So, you don't want number 2? What's wrong with it?
CM13: It's a little above my head.
I: Ok, so after selecting, pull down send to email
CM13: Oh, that's how you did it before, eh? (with the articles I sent him)
I: Exactly, and make sure there is the pink ribbon there to make sure it was sent. And now it'll be in your inbox. Remember if there is a green bar in the icon, you can get the full text of the article.
CM13: What do you mean?
I: Remember the icon before, if it is green the article is available.
CM13: Oh, I was looking at the pages in the back there, there were lines in the icon, I thought the pages (i.e. the article) were there.
I: The green bar is what you want to look for.
CM13: Oh, here we go (find a green one). Oh, I see, but there is a different one here- there is a green and an orange.
I: Yes, I will explain that in a second.

Send to text:
I: (I explain how to use this tool – that it is good for saving to the computer, strips the formatting)
CM13: Oh yeh, I know what you mean, all these different characters and stuff like that.
I: Right. Ok, let's send the three articles to the clipboard. See it says three items were added.
CM13: They don't have a place on there where you can save it?
I: Well, they have my NCBI, but I am not sure if you can save your stuff there. But you can amalgamate your searches in the clipboard and go back and decide which ones you want to keep.
CM13: So I lose them 8 hours after I start doing this?
I: No it says after 8 hours of inactivity… and you can't forget to send them or save them before logging off
CM13: Ok, that's pretty straightforward.
I: Great.

Limits:
I: (I explain limits; another way of refining the search, we go through the different options: humans, animals.
CM13: Well, I would think that if the subject is childhood obesity then the subject says it should be humans and the age is already originally in there.
I: Yes, that's true its inherent in the subject. But that's not always going to be the case. … but it's a good point.
CM13: Ok.
I: Ok, so we have a few more things to choose from here: publication dates…
CM13: So we have to be specific here?
I: Well, you can choose to be if you want to.

CM13: Ok.
I: And here you can choose what kind of study or research you want to find.
CM13: Ok.
I: So we are searching now child obesity and nutrition (Search 1 and search 10 combined), but we only got 4, so we may not find very much, so may be we should take the Canada out and just to Child obesity with the limits on.
CM13: Yes, ok, that is fine with me. Umm, can we reset the limits when we want to?
I: Yes, definitely. So where would you have to go to take of the Canada part?
CM13: Oh, I would have to delete the 10.
I: Right. Very good. 147 articles.
CM13: Ok.
I: All of them have abstracts, are in English… so do you see what the difference is between limits and combining using history?
CM13: Definitely
I: What would that be?
CM13: Limits narrows them down more – you set limits.
I: Right it affects things around the article- not necessarily the subject matter. With Boolean you are creating or recreating the topic.
CM13: Oh, yes, that true, I get it. Ok, and what was the term you were using? This is open access? And EBSCO isn't?
I: Well here you don't necessarily have access to all of the article, but to information about the articles..
(We turn to look at green and orange article)
I: (I explain the difference between a green and an orange article: one being part of Pubmed plus, and one being as part of another journal web site.)
CM13: I like the idea of staying in Pubmed to look at the articles. I do think I would prefer to find the Pubmed Central articles. Now the quality of the databases, how similar are they?
I: Well, the information and Pubmed and in Ebsco are pretty much the same. But Pubmed does not necessarily provide full on access to the articles.
CM13: Well that's the same thing as in EBSCO- you don't necessarily get access to all the articles form there either.

History:
I: Ok, now go to history and look at the searches again.
CM13: Ok, so this gives you all of your searches.
I: Ok, and you still have hour limits on, so you might want to take them off.
CM13: Ok, so if you want to search something else you have to take them off. Oh, ok.
I: So, go to Links.
CM13: Ok, so this is going to give you papers by the same authors and stuff?
I: No, but if you do want that, then, do you see how the authors names are underlined,
CM13: yes
I: Ok, if you click into the names then you get articles by the same authors.
CM13: Oh, ok, that's cool. (He looks at them).

Books:
I: Ok, so click on links and then books.
CM13: Ok.

I: So do you see how all of the words are now highlighted?
CM13: Yes.
I: Ok, what do you think that might mean?
CM13: well, I don't know. Umm, is it connected to other articles?
I: Very close- well these are words that are found in books.
CM13: umm, ok…
I: So all these terms appear somewhere in this book (We click into one of them).
CM13: Oh, I see (he looks around some more).
I: So, if you were interested in finding a book on obesity then you could go to books. And notice that the database that you are in changes. You see it says
CM13: Bookshelf. Yah. (He looks through it some more- clicks,)
I: Do you see that is the table of contents.
CM13: So these are all online?
I: Well, the entire book is not online, but information about the book is. So you can find books on a subject- may be go to your library to see if they have it.
CM13: Oh I see… ok.

Linkout:
I: Yes, so if you have an author you like particularly, then you can find more articles that they might have written. But, actually, Linkout is something different. Can you guess what it might be?
CM13: Hmm, not really.
I: (I explain that link out is where you go to get the articles, see how to get at them, sometimes you can find the article for free, or you can check to see who publishes it)
CM13: that makes a lot of sense. It would be useful. I guess when I start needing to get the articles and stuff it'll make a lot of difference to me.
I: Well, when is your project due?
CM13: on the 28th. It's really small, we pick a topic and do a commercial or skit or something like that. But I need certain facts and stats for it.
I: Ok, so for that will you let me know if the articles I sent you are helpful.

Favourite tool:
I: So what is your favourite tool?
CM13: I guess the linkout. Because, I am comparing this to EBSCO, everything else they have the same thing but I didn't see the linkout before. Its a lot harder on the Ebsco to find the article.
I: What do you think about access:
CM13: the full article is important. If the abstract was there, I would need to have a subscription, and I would have to go to Anne to find it and stuff, so yes access is good.

<u>Interview with CM14</u>
I: What year are you in?
CM14: Second year.
I: Do you use Pubmed a lot?
CM14: Yes, for sure at least once a day.
I: Why? What is your behavior around it?
CM14: Usually just because I have lots of questions so I like to answer them. I use it as a method to have access to articles. So, I don't really read just the abstracts. I just use it and try to figure out does our school have access to the journals.

I: So you don't use the abstract to weed out…
CM14: The good from the bad articles? Well, you can to some degree but the abstracts usually don't show enough about the methods of an article, you need to look at the full article. It depends on the method if the paper is actually good.
I: So what is your starting point? You do a search, and then what?
CM14: I will do a search, often times I will look at the authors. Like now I am starting to become more familiar with some of them, depending on the topic. So I'll see lets say I am looking at low back pain, XXX, and I say ok this guy is the man for low back so I will trust this paper. Versus some guy who has got a paper that seems really interesting but I have never heard of this name before, the abstract might tell me a bit of like methods but it is usually one or two lines, so you need to really get in there, read the methods and find out of this paper is actually good or not.
I: Do you use mostly complementary medicine journals?
CM14: Actually … I have never ... through Pubmed you don't have too many complementary medicine journals. They have JMPT- the only chiropractic journal really. There aren't that many. There is another search engine – SINAL – I don't know if you have heard of it but that one has a lot of alternative medicine papers. I never really use it, I only use Pubmed.
I: Ok, I sent you 4 articles and they were all about lateral epiconditis –
CM14: Also known as tennis elbow.
I: I found a lot about tennis elbow, so let's look at Miller S, because you are interested in treatment, right?
CM14: Yes.
I: [We open up his email inbox, click onto the emails I sent him, and open up one of the open access article from BMJ. The Pubmed window opens up when we open the email I sent him. He did not have time to read the article before I sent them to him].
CM14: Will you be talking to me about the different icons that open up?
I: yes, I will be going through those differences. [We open up the article and I explain more about the Pubmed environment]. Here we have an article by BMJ which is free
CM14: Yes I have heard about and used BMJ – I like it a lot. It's good.
[we look at the full text of BMJ]
CM14: Should I get the PDF?
I: Hmm, do we need to get the PDF?
CM14: No… it just looks nicer. I'll just get the abstract.
I: Let me know whether you think it is useful…
CM14: See like right here, it says (reading from the abstract) "evidence which is based on the Cochrane review of the subject." This review is like topnotch, so … the purpose of it, its called a Cochrane collaboration, and we don't have free access to that … it would be phenomenal if we had access to that, the purpose of it is to specifically answer questions that are clinically related. Its only about clinical stuff. Its just like this tennis elbow- it comes up with all the research that's been done out there. Like the RCTS on the subject. Its all based on a strict protocol- like how many people were in the study. It is well designed and then it comes with its conclusions its like a place where a lot of medical doctors and chiropractors, or whatever, if they want to know something, if you want to know the vast information about a subject you should go to Cochrane reviews.
[He reads some more]
I: Ok, what do you think, useful? A good article to start with?
CM14: Definitely, especially since it is a review of all articles out there. So when you look at something like this you can read it and then you can use the article to look at all the references and get more from there. So the readings are nice for that reason.
I: Ok, now go to µwww.pubmed.com§. Ok, put in lateral epicondilitus.
CM14: (He explains to me where the lateral epicondil is and why it gets inflamed).

I: Ok, we'll start by looking at one citation and I will explain things as we go. (I talk to him about the importance of limiting searches. We start with Boolean principles: lateral AND epicondilutus.) That's called Boolean searches. Have you ever done this before?
CM14: Yup.
I: Oh… I wanted to teach you something.
CM14: For some reason Ebsco does not let me do more than one Boolean at the same time. It's kind of weird but anyways …
I: (I explain to him what the icons symbolize—abstract vs. no abstract). So from first glance you can see whether there are abstracts or not…
CM14: If there is no abstract I don't know anything about the paper. Unless, if the title is interesting, I'll check and see if we have access through the school- that is if I think it is interesting… without an abstract you don't have anything. Can I just ask you a quick question: when you were doing Boolean, would you get the same results if you did 'lateral epicondilitus AND treatment' versus 'lateral epicondilitus treatment'?
I: The logic will be a little different. Let's try out the different ways of doing it. And we'll also use the history function. Have you ever used this before?
CM14: No.
I: Actually [the librarian] taught me how to do this.
CM14: She's awesome.
I: Yes. So you see there is a green line there …
CM14: Yes, can we click on it?
I: Well, let's go through some more unless you are interested in it.
CM14: No that's fine.
I: Ok, because there are actually some more down here to look at. Sometimes there is one with an orange bar too, and that means something different. Hopefully we'll across one soon.
CM14: What is it?
I: It is a green bar and an orange bar, and the orange means it is free within Pubmed central. So you don't have to go outside Pubmed to get it.
CM14: Ok.
I: Ok, let's do 'lateral epicondilitus and just put treatment in there without the AND. So we got items 1-20 out of how many?
CM14: 30?
I: 592.
CM14: Oh there it is… oh this means 30 pages…
I: Ok, so let's put the AND in.
CM14: Does it have to be capitalized?
I: No, ok, 592- that's interesting. Let's put 'Not' in there. Oh, 24…
CM14: Let's say … oh no, that's ok. May be it's because, well, when you search it does it search the whole abstract or the article?
I: The meta-information.. (I explain what meta-information is). So I think it searches the meta-information.
CM14: So may be for some reason a lot of those article use the word treatment and not therapy. And I don't want therapy I want treatment.
I: So let's go to one of the articles that's useful to you – go to history, and go to 803. Let's try to find our original article by Miller.
CM14: Does it matter if we click on that versus that?
I: No they are they same thing. Ok, so this is our initial article. Look it over, and if it looks good to you we will click on related article…

Related Articles:
CM14: Yes, this seems really interesting.

I: Ok, let's go to related articles and see if it brings you to other articles that are good for you. It works in a different way than Boolean, so we might find something better.
CM14: Ok. I think I have seen sometimes they actually show keywords. So I just assumed that when we hit related articles that it would search all of those keywords…
I: hmm, I don't see them here...
CM14: Yah, me neither…I'd say, may be 75% are related to what I am interested in. Some of these have to do with surgery, which I don't care so much about, which is fine, They are all related to lat. Epic. So overall, I'd say 75-80 of these are related to what I am interested in reading.
I: Great. So, related articles compared to Boolean search … what do you think?
CM14: So, which one came up with more relevant articles?
I: Which one would you use more? How would you do it?
CM14: I think I would do a mixture of the two. If originally I only saw a couple that seem interesting then I would obviously check related articles. Or if I saw an article and saw that it was exactly what I was looking for, then I would hit related articles and see what comes up.
I: Great! Good answer! Let's go to history. This feature basically tells you what you have been doing and what you have done (We look over his searches).
CM14: What if I were to do things like chiropractic? And I wanted to get them all?
I: Put a star and it will bring up the root of the word.
CM14: It's a star? I knew it was a star for Ebsco…
I: The star isn't really specific to databases it is a general search technique for anywhere.
CM14: Ok… I got it…
(We do a regular search for chiropractic and call up a large search. We go back to history and combine the search with others….basically Boolean using history)
CM14: This is the exact same way that EBSCO works.
I: It is?
CM14: This is great- it was one of the reasons why I like EBSCO and not Pubmed. I didn't know you could do this on Pubmed. Its good. That's what Anne tells us to do to do search: she tells us to find the MeSH terms, and then go back and it will all be saved in your history which I did not know they had in Pubmed and I would say Lat. Ep. Mesh term, hit search, and then go back, I would do treatment, Mesh term, and hit search. And then I would go here and then combine the two and then go, instead of doing the Boolean. I don't know what the difference is, but that's the way she likes us to do it. May be for history purposes…like it's easier to go back and know what you did… I don't know.
I: Well for Boolean it can get really complicated. The reason why she wants you to use the Mesh terms is to be certain that you are suing the right search terms in your searches. And then I assume she wants you to stay organized, which is the benefit of using history which is much more visual. This maps it out for you to make sure you don't get lost. (We then conduct another search, we get three articles). Do any of these articles look useful to you?
CM14: Yah, definitely. This one would be interesting. I have never read anything like this before, but it is in German.
I: Ok, well you know what we can do, open it up …
CM14: Yah, this will be really interesting because sports medicine doesn't really publish chiropractic stuff, so it would be interesting to see what they would have to say.
I: Oh. Well then go back.
CM14: It's all in English here – but may be it's only this part here ... not the whole journal.
I: Instead of hitting on the article, go to related articles. May be we can find another one

like this one. Scroll down, we are looking for a green one. And let's do some email stuff. Choose a few that you love and email them to yourself.

Send to Email, text and clipboard:
CM14: Ok, here we got a couple … (he reads) … this could be interesting.
(It is from BMJ. We select it and go through the email function)
I: have you ever done this before?
CM14: Not through here I haven't. (We send the email). See, that's the nice thing about Pubmed too, is, with Ebsco, if you use the back button up here it screws everything up. You have to use the back button in system.
I: And it's not intuitive to you?
CM14: No, it takes a lot of practice. (We go back a few pages to our search). So, I could have also used history to get back here …
I: Yes. Definitely.
CM14: I was just wondering.
(We go through send to text and clipboard).
CM14: So, this isn't my computer clipboard. This is a clipboard in Pubmed..
I: Right. So, you can do as many searches as you want, say them to clipboard as you are searching, then send to text and save them on your computer.
CM14: So, let's say I sent 4 items to my clipboard. How do I send those 4 items to anything? How can I view my clipboard?
I: Scroll up… do you see there is a little tab?
CM14:I actually never look at those…
I: yah, I know but they are actually the steering wheel of Pubmed so it is good to know those. Ok. So hit clipboard.
CM14: Alright. And then I can go from here and send it out. Oh, you can order papers…. I don't have money to order papers.
I: So this is a way of organizing searches and sending them to yourself.
CM14: great!

Limits:
I: [I explain Limits – ability to drill down re: meta-information]. Something like 'languages' is meta-information.
CM14: So all this is meta-information (points to the limits fields).
I: Exactly. So here you can be specific about your meta-information. You can say 'I only want articles about English, or about humans. So put a couple of limits on and lets do a search.
CM14: Can I put more than one limits on?
I: Yes, of course, but only one per tab.
[we are looking at the limits, and we see an option under type of study and we ask what one of the types refers to – he theorizes what it means]:
CM14: I imagine that would mean it refers to suggestive guidelines – how you should treat it.
I: Ok, so you have chosen your limits and now it is time to search. Choose a topic.
CM14: Does it have to be the same topic?
I: No, you can choose whatever you want … choose something useful so you have a good experience.
CM14: Do you know how to spell placebo?
I: That's right…
(We do a search and find nothing.)
CM14: Hmm, it is probably because of this (points to clinical trials limit). Let's do randomized control trials…

I: Ok, so you found a bunch of articles … how useful are they?
CM14: umm, not really that useful.
I: No? How come? Is it the search or the limits?
CM14: I think may be I should re-define my limits… this one here, seems like it is going to show that putting a saline solution, which is basically like salt water, works just as good as putting a actual cortisone shot in your knee. I think I have seen this one before. Some is useful and some is not. That's sort of, may be what I would do on that one is hit the related articles button. Like the other one, wherever it went. Let look at this article it looks interesting.
I: Ok, just a couple of things: look up and see the pink ribbon across the top of the screen. There it shows you what limits you have selected so that you always know what's on- that way you do not forget. So, see that they are still on and they remain on till you take them off.
CM14: Ok. What's next up?
I: Can you tell me whether the search worked well? Do you get what happened? Why things came up? And are the articles relevant to your interests? (We find an open access/ Pubmed central article).
CM14: The search does show some interesting things, but I was particularly interested in the placebo effect and none of those really were specific to placebo effect, so I guess it did not find anything.
(we turn to the free article which is an BMJ article.)
I: Ok, so click on the free full text article in Pubmed Central.
(We compare the free full text in BMJ against the text in Pubmed Central). I wonder if BMJ is now Open Access?
CM14: Actually there was an article that I was interested in looking at and it said that it would not be available for about a year or 6 months, or something. I think it was this journal, I am not completely positive.
I: Ok, so that was Limits, and once again related articles. And another way to drill down in your searches.
(CM14 reads some more on the web site… and browses around …)
CM14: I imagine this would be only in BMJ, right? (pointing to the various features available in the BMJ web site).
I: Yes, that's right (He looks around some more). Ok, so click out of that and let's go back up. So, we have gone over limits, history and clipboard, Boolean related articles. So let's go to links and Linkout. You seem to know about Linkout …
CM14: I have used it before, but I am sure there is stuff on there that I have used before..
I: Let's start with books. Check out what happens with this abstract when you go into books. See how everything is highlighted?
CM14: Yes,
I: Ok, well that means that these terms actually appear in books. It doesn't necessarily mean that the books are about this term, but that they appear in the book somewhere. So choose one that will be interesting … how about acupuncture?
CM14: Ok.
I: So, in HSTAT there are 200 occurrences of the word acupuncture. (We look at the TOC and then some more info about HSTAT). Books function takes you to books that reference your subject…
CM14: Umm, that's interesting. But I don't know… once you get into reading research articles, they are much better than reading books. Books are always behind a few years.. by the time its published and stuff, there is a lot of bias behind the author of the book – reading an article you get the actual truth. Certain books are good – like that McGill guy (an author he has read) that I was telling you about before. He has a book on

lower back pain and so you'd believe more stuff that is in the book and he heavily references in that book whereas some books might, like a book on low back pain might be about how to treat your own lower back or whatever, and it may be a good book, but you want to look at all the references. It might just be someone's opinion… you know what I mean? And the person's opinion might be good but you want to have some references. So that's why books, you gotta be careful with reading books.
I: So you find that books are more subjective and biased than regular articles?
CM14: Yah, and even articles you gotta be careful with. Sometimes you can see, even on Pubmed, there are certain journal articles, well there are certain journals that are highly regarded an you think they don't let any crap in their journal. And there are sometimes some articles that are published and you look at the references and there are sometimes like four… and the paper is like 6-10 pages long and it is all, like personal opinion.
I: And not research …
CM14: Sometimes its fine, it'll say this is a commentary, and in that situation you know… but sometimes they don't classify it as a commentary when it is one. So its kinda sketchy. But those types of things are similar to books.
I: The commentaries…
CM14: Yeh. It's good to some extent. It's a good place to have general knowledge… like, if you know nothing about a topic, research papers are generally very specific, so unless you know something about the topic already looking up research papers may not be that useful for background information. I think I would buy the book if I trust the author. But how would I trust the author? If I saw his name in Pubmed a bunch of times. If I have never seen his name before, then I would be very skeptical.
It's confusing. Pubmed is a trusted search engine, so anything on Pubmed you would consider trustworthy regardless of the journal because if it is in Pubmed then the journal is trustworthy. But you still have to use your brain. You have to look at the methods. And somehow for some reason some of these papers get published and they are crap. It happens, I don't know why … do you know, are all the journals that are in Pubmed, are they peer reviewed?
I: Yes ...
CM14: Yes, there is a guy here, Dr. XXX, who is a reviewer for one of the journals, actually a few guys on this floor review articles for journals, but anyways, I want to talk to him because there is this one paper that came out in a journal and it is just awful. Like, I want to say did you read this paper? It is just awful. I don't know how the process goes but I want to find out from him for that particular journal, what's the process? The conclusion made did not reflect the results that were found. It was just, how it was even allowed to be published is beyond me. So I want to find out what's going on.
Because, once you are in a practice you don't have time to read all the methodologies of every journal. You gotta trust something or someone, you know what I mean? Because you don't have time to read everything in detail… ok, I have said enough about it …
Like now that I know XXX is trustworthy, you never know there might be a paper that he throws out there that may not be that good, you might find mistakes here and there… and whatever. Even though the person seems trustworthy you still have to read the paper. Be critical no matter what … but when you are practicing how do you work full time, read and then, not only stay up to date but critically appraise this stuff. It's a lot of work … it's a full time job just to keep up to date.

Linkout:
I: ok, so you know what this is, it is like a roadmap. (I explain more).
CM14: Ok.
(He was already familiar with Linkout so there was not much to say. Also, the library of

the CMCC already had subscriptions to the major publishers.)

Favourite tool:
CM14: Most useful tool is the alert one – we didn't check it out together. Out of the ones we did go over, definitely the search strategy using the history.
I: And number 2?
CM14: Umm, the Linkout button is easy to use.

Interview with CM15:

I: Did you get the article?
CM15: Yah, well, it was very long. So the big article was like 100 pages. So I printed the summary of it, and just looked at it, some of the diagnosis stuff was not quite what I was looking for. I'm not looking for the diagnosis stuff, I am looking for the clinical stuff that we would do in every day practice, not so much the ultrasound and MRI because we do not have access to that. So I am looking at those kind of tests.

In terms of understanding the stats, I'm not knowing too much about the stats but I kind of got the general idea of it – specificity of .96, that's obviously quite good…

I: So why were you interested in the specificity stuff?

CM15: As part of our school work, with our school, we try to research the different orthopedic tests and see if they do what they claim they would do…

I: The orthopedic tests are different than the MRI's…what are they, X-rays?

CM15: No, they are like, we would take the arm and move it around in a certain position and if that causes pain then that would be indicative of a certain problem. It is more of a hands on type testing with the knee or the shoulder…

I: Ohh, I misunderstood then. So, then the article I sent you didn't really suit your needs?

CM15: It actually had a summary of clinical assessments which was good. But it didn't elaborate too much on what the tests were, but then it went on to the more medical type testing which was stuff we don't have access to directly.

I: So you need more about the practical tests, right?

CM15: Yah, I guess, I'm not too sure how we go about researching them, like would you just put in orthopedic tests, or clinical? I'm not to sure how one would come up with that…
I: Ok, well let's give it a shot.

On to using Pubmed:
I: So, the whole point of doing a search is to refine and drill down to specifically what you want to know more about. The point is that what we see right now (pubmed home page) is just an interface, and behind it is tons and tons of information. So, up top, are all of the databases that this index, Pubmed, searches.(Protein, genome, structure, are all

databases).
CM15: So, I see…when I would come to use this sometimes I would always wonder what these were…

I: Usually on a web site, there is more than one point of access to the same thing. So you can access the databases is provided here too…

CM15: ok .. I get it.

(I go over the different things we are going to run over. We start with a simple search of orthopedic tests.)

Boolean:
I: Ok, do you know what Boolean searches are?
CM15: Not exactly.
I: Well, just with words you can make your topic much more specific. We will start with "Orthopedic tests AND shoulder or AND rotator cuff" it would make our search more specific. You can also change thing by adding OR or NOT.
CM15: Yah, I see how that changes things.
I: and so just by that, without any of the fancy bells and whistles of Pubmed, you can actually specify and limit more.

Limits:
I: We can do something like this as well with limits. [We go to limits and select it]. So here you have all of the different forms of meta-information. DO you know what meta-information is?
CM15: Kinda combining everything, is that what it is?
I: It is actually information about information. "What is common to all entries in a database? Authors name, title, abstract, and every medical article has a methodology.
CM15: Ok, I got it.

I: So meta information is name for information about information. So here, you could further limit and also do a Boolean search…
CM15: Uh huh-
I: So its further and further drilling down and limiting scope. Now why don't we try just orthopedic tests?
CM15: Ok.
I: Ok, so look through this list and tell me whether these at all pertain to your interests that would be great. And then I'll show you so more stuff.
[CM15 Looks through the list]
CM15: Something like this will be good.
I: Ok, let's take a look at this one then,: [I explain the components of the citation, including the icon indicating whether the article is free or not]. We open up the citation and look at in detail. This is not a free article.
CM15: Well, I could get the article for free through the school, but if I wasn't attending the school, I couldn't. Once you open a practice you need to be a member of the school to get the articles. If you don't have access to a library, like a hospital library or something, then you cannot get it.

Abstract:
I: Exactly! So, the next question would be whether or not you actually need the article to read, or whether there is enough information within the abstract – may be some abstracts are involved and complex enough to give you enough information…
CM15: It's a good summary, but in terms of some of these tests that they are doing, I'm not really sure what they are so if the article had pictures and stuff, cuz may be these r new tests that have come out that the school hasn't taught us yet, and they might be better than the ones we learned…
I: So access to the pictures would then be really important.
CM15: Yah- if the article has pictures. It looks like this study is a meta-analysis study where they just take a whole bunch of studies and look at them. In which case, of course the abstract is not enough information- I would really want to get my hands on the whole article.

Related Articles:
I: You're right- it's a review study. So if this looks interesting to you, we do have another option in Pubmed.if you click on the related articles button it brings you to articles related to that initial study.
CM15: oh yes, that's a really great one. Should I click on it?
I: sure!
CM15: Glenoid laberor tears- that's tears in the shoulder, in the cartilage and stuff that gets torn, and they are comparing it with MRI's versus what we are doing in our offices basically to figure out if it is torn or not. (so related articles is really useful)
I: Oh, so it is exactly what you are looking for… ok well then I have another tool for you.

Send to:
I: (explain the send to email function to so CM15 can send this article to his email box). I explain all of the options to CM15, and explain how it would be used.
CM15: (puts in email address and information—and sends off the article. }
I: So that was related articles. Some times related articles takes you in a whole other direction than you might want to go, because its work off an initial article as opposed to search terms that you put in the search field. Its one way of drilling down, but could take you the wrong way. So, if you can go back to conducting a better Boolean search…
[we look up orthopedic tests and shoulder, articles that we got already tend to come up]
CM15: These are all really good to, eh?
I: well, take your time and look through them. Click into them and read the abstracts to be sure we are getting the exact one you want.
CM15: oh, so I clicked on this one, and even though it did not say it was free, when I clicked into it, it said 'click here for the full text article'.
I: If you were to click on it outside of the college environment, it would ask for a password or it would ask you to pay. So, in as sense you have access to it, but its isn't free.
CM15: They make it available but it's not free through Pubmed.
I: Try clicking into it and see what happens.
CM15: It says: "access to this article will depend on your personal or institutional

entitlements."
I: Which basically means you need to be a subscriber- whether through membership in a school or other org. or via a personal membership.
CM15 emails the article info to himself to try to get from the CMCC library later on. Uses the 'send to' function that he just used.
I: well done—you're an expert!
CM15: well, I have used Medline before and Ebsco, so I am kinda familiar with how they work.
I: Ok, great. Do you want to finish looking through the list (from before, the related articles list).
Some time passes while CM15 looks through the list of articles to endure that the ones pulled up are useful to him.
CM15: Are these sorted by date?
I: Well, I think they are sorted by relevance, and date is a form of relevance because usually it is the most up-to-date research that is most relevant. Does that make any sense to you?
CM15: Yes it does.
I: Lets take this article and research it by putting the author's name in. This author has written a lot about diagnostic tools. Because she is the first author on our original article.
CM15: Right, because the first person on the author list is the one who put the most into the article.
I: Right. Ok, so using this article, let's go to related articles form here and see whether or not it gives you more articles that you like as compared to our Boolean search from before {orthopedic tests and shoulder}.

[we then hit related articles from this article to see what happens. CM15 reads through the list. We find two free ones.]
CM15: it seems like with this list there is a lot more radiology stuff. Like MRIs, diagnostic ultra sounds. In the other search there was more on what I would be doing in my actual practice. Plus there were 4000 hits.

Links:
I: Links is the next tool we are going to look at. We have two options: Books or linkout. Let's start with books.
CM15: Oh, it's a database we are going into…
I: Exactly. So, notice we are going into to the books database (show him the change up at the top). It is a database of information of books about health science, and about your subject.
CM15: So these are textbooks?
I: yah, probably more textbooks than anything. It basically called up the book, because within this book, are your terms: 'diagnostic tests' and shoulder pain…. Now there is no guarantee that it would go into detail about it, but we can look at what we do talk about because we have access to the table of contents.
CM15: [enthusiastically] Oh, ok!
I: So if you scroll down or hit any of the blue underlined terms, [CM15 clicks on clinical exams] So these books have the term clinical exams in them.
CM15: ok, got it.
I: Anything underlined in blue {clickable}, has a book that relates to it. It may not be

about clinical examinations, but it'll at least be there.
CM15: So these are books, or databases of books? (asking about a book that has come up that has everything underlined).
I: Nope- this is one book, that is probably a general health sciences/medical book. Actually whenever I do this, this book always comes up… it means the topic you clicked on is mentioned in this book.
CM15: Ok. I got it now.
I: Let's take a closer look. Let's click on 'Cancer Medicine'. And this is what happens. This book is completely online, and this is the table of contents of the book. So
CM15: All the books that are in the database are full, online books, that are free?
I: Well they aren't all online books. Some might be some may not, but there is info about them online, and then you might be able to order them. This one is only $427.00! {We laugh}. Let's try opening some other books.
(We open up 'shoulder pain').
CM15: So these are all the books come up?
I: Yep. And this is how many times shoulder pain is mentioned in the book (point to the number next to the term.) Have a look at it to get a sense of the type of information is in the books. {He looks at it}. Do you think you'll use something like that?
CM15: Yah. Especially if you are looking to get if it comes up that you want in your library. It may help me find a book that is already in the library. I could look it up here, see what's mentioned in it, and then see if the library has it. That way I don't even have to look at the (actual) book to see if it has what I want.

Limits:
I: Let's try limits: we'll do this one combined with a Boolean search. Is there any other body parts you want to try this time?
CM15: Let's try clinical exam and hip. I know with Medline I can put a star and it'll pull up anything with exam in it…
I: Yes, that is a pretty universal search technique—you can do it with any database. That star thing has more to do sentences and search techniques than with Medline or Pubmed. The more you know your techniques, the better you will be in general with databases.
[Some time passes while CM15 looks over the articles. He finds a Danish article that he likes, and so CM15 clicks on related articles for that article, and it does actually find articles related to that article.]
I: It might have been a little difficult though because there was no abstract for it to work with so it relied on the title.
CM15: Oh I see, but there are a couple in there that I would like to see. All in all, Useful!

Linkout:
I: ok, so let's chose one and go through another tool. Linkout- it's the other tool that links goes to.
CM15: Ok.
I: Basically, this gives you a map to where your article is. So if you click on libraries, and click on any letter in the alphabet, you will see this is a list. This is telling you that this article is available in print at the following library "Affinity Health etc…"
CM15: That's kinda neat. So I might be able to request through the library an article, it might be cheaper to get it. That's a really cool thing, its good if your in a big centre…
{We look up to see if UofT is listed in Linkout and also if the CMCC is listed}.

I: this is a really good tool for a free article, but sometimes it is confusing to find the actual free article, and if you hit linkout you can find it easily. It might be the fastest route.
CM15: So, if it was online, or free online, would it indicate here that this library has it, or would it just be?
I: well, let's have a look at a free one and see what linkout does for it.
CM15: Ok, so with this one, it means we can get it for free here? (Points to the link to the free article in linkout)
I: Yes- and also that the other companies have the same articles; the proprietary companies, Ebsco, Ovid and Ingenta.
CM15: The proprietary articles meaning? (puzzled)
I: Well, by that I mean to say that someone owns the articles.
CM15: So someone owns Ebsco?
I: Well, they don't provide free access to their articles…
CM15: oh yeah- you need to subscribe to it.
I: Exactly.
CM15: Ok, so when I click on the proprietary ones, I can download the PDF. Yes, that's a useful link.. it is a way to clarify what is going on in terms of access to the article.
I: Exactly.

Limits:
I: Ok, so let's go to the homepage. And we'll start a new search but limits on it.
CM15: ok, let's try some more test: but this time sacro-iliac.
I: Sure thing. {CM15 types it in…} And now, hit limits, and we are going to limit the search but not in with the Boolean technique. This is a different way of limiting the scope. It limits by being more specific through metadata. Hit limits…
{Together we do to the Limits function, and check of some limits. We choose men in terms of gender, we look at 'subsets' and choose core clinical journals, for languages we choose English, and we choose humans out of the humans/animals function}.
When we arrive at the "all fields" field, he expresses confusion and mistakes it for being able to use the fields as search criteria.
CM15: What does affiliation mean?
I: Which research centre the author is affiliated with.
CM15: So you can even search by research centre?
I: no it will only show you that field. This deals with what field come up when you do a search. You can limit to a particular place- but it wouldn't be here in limits. Limits is about the meta-data- the information about information. What is common to all journal articles.
CM15: Oh I see.
I: in terms of limiting by publication types: you can choose to see only lit. reviews, clinical trials etc… you can limit your search by choosing age: that would be participants in the study only between particular ages.
[we go through the rest of Limits]

I: Ok, so now, note that when you hit go, you have, by using the Limits functions, limited the scope of your search. We are only going to get articles that are:
written in English

-only articles in core clinical journals
-Only articles about men- where research is limited by gender.
-Only articles with men between 19 and 40
So you are really funneling and funneling your original search topic.
CM15: That would be great if I had a patient come in with a problem, and I had to really specify my search according to his needs.
I: Right, may be you have specific search needs, and don't know how to create a logical search string in the Boolean method. So you will ultimately, you will get less information than originally.
CM15: So what is a core clinical journal, exactly?
I: its basically the major traditional journals- like BMJ…
{we find nothing] Ok, so we have limited too much. Do you see how after we finish the search, there is still a check mark next to limits? Well, that indicates that the limits are still there. You have to take them off...
[we take subsets off, and find 50 articles. CM15 looks to see if we they are good]
CM15: Yah, these ones are good, this one we have online through the school, so that's good. {in other words it found relevant articles}. Oh, that's a good one.
I: Well, then click on it, and we'll do related articles from it.
CM15: Yes, this is getting into more treatment and stuff, and outcomes of treatment and stuff. All these journals are ones we use readily in school.

<u>Send to, Clipboard, and Save as text.</u>
I: Ok, now just click on a bunch of them, and I'll show you another little feature. This one is not about searching but about saving. Because when you use Pubmed, you have to do two things…
You have to find and to save. So, hit the save to text button, hit that one…If you were in the library, this would be a lot easier to print out than the whole html page.
CM15: ok.
I: But if you were working on your own computer, you could just save it as a text file ontop your computer.
CM15: Oh, ok. Does it save it as a..
I: As a text file. And now save it on the desktop so we don't lose the file. And we can do a similar thing, but we'll send it to the clipboard.
{We click into clipboard and save three articles to the clipboard.}
I: Do you see what it says about the clipboard—saves up to 500 articles, and keeps there for up to 8 hours. Well, with clipboard, you could save a bunch, and then afterwards look over the list and delete the ones that may not be useful. Or you could send them to email or text. It's a way to amalgamate searches instead of doing it one at a time.
CM15: So right now, I can just click in, and then send to email – oh, ok. Is there a feature that selects them all?
I: I'm not sure. I'll look into it- but I don't think so..

History:
I: Let's look at another tool. Are you familiar with history?
CM15: Yes, it is good to combine terms.
I: yes, that's one way to use it. But you can also look over your previous searches. If you want to remember your past searches, you can look them over.
CM15: That's a really useful one – it is great for combining the terms. It is an essential

tool.
I: So you use it mostly to combine the terms. IS that correct?
CM15: Yes, say if I was using EBSCO, I would type shoulder exam, hit go, it would come up with 100 articles, I would type exam and combine the two through history.
I: Oh, so it is like a Boolean search but high –tech?[2].
CM15: Yes, I guess so.
I: So it is provides two basic functions- to relocate you in your searches if you have gotten lost or disoriented or you ant to back to some, and also to combine searches to mimic a Boolean search and create a search string.
CM15: OK. So when you research you might just take a left turn and you meant to take a right turn so you might use it to go back.
I: Right.

Favourite features?
" I really liked the related articles link. That was, I have never seen that feature before. Especially if you have an article already, its really great."
"the clipboard is essential too – to save your work and your searches."
" and the send to stuff, with the email, even the send to text was a great function."

Future use?
"Now that I know more about it I think I'll use it more. I think I'll be able to manage and navigate through so I'll be able to use it. I also hear that Pubmed has more information in it so it would be smarter to use Pubmed because I could find more. Every time I would go to Pubmed I would have trouble figuring it out because I was used to ESCO. But now that we have had this session I'll be able to understand it a little bit better. Especially this related articles."

"My clients may come to me with questions and information, and I would have to look things up to help them. I could see that happening. I definitely think it is important to read the literature. I for sure plan to do that. They (the school) thrust that down our throats), make sure you are up to date on that. And trying to as a student, subscribe to the etalks and such."

"Abstracts are definitely important. They don't give you all the information you might want, like pictures and stuff, but they give you a general idea of the article. 'Pictures' is a big issue – because you can't just describe these tests. You have to see them and the picture."

Interview with CM16

I: (I explain homepage to him) (We search fibromyalgia to find the article I sent him. I explain the search list to him and the idea of drilling down, I explain Boolean.)

Boolean:
CM16: So do you have to put AND between each one? Is that if there is more than two?

---

[2] This is the technique that the school's librarian drew form in order to teach the students Boolean searches. It could explain why CM15 was familiar with the history function and why it was an "essential" tool for him.

I: Yeh, well you are going to want to write it in to create a more complicated search string. (I give him examples)
CM16: oh yes, it is one or the other…
I: It is called Boolean searching… (I then explain to him the differences between the different icons… lines, no lines, green, orange).
CM16: Yes it's annoying to find articles that do not have the text…
I: How much do you rely on abstracts?
CM16: It's difficult to say… most of the articles… well I don't bother thinking about the abstract because I need to read the articles I receive through the service.
I: well, let's look at an abstract, read it over, and let me know what you think- if it is enough information to judge..
CM16: Ok, so just any abstract?
I: Yup.
CM16: Just from scanning it looks complete enough to know what's gonna being the rest of the article.
I: Does it give you a sense if you want to read the article?
CM16: I think it does. This will tell you right away. It says the conclusions and… well, let me look at a different one. I am trying to find one that is not as bio-chemistry to read, those ones are terrible to read. Here is one: physical activity in women with fibromyalgia. (He looks at the abstract)
I: Ok, let's try to find more articles that are similar. First of all, this one is not free full text. But you could see if CMCC had access to it. And follow up from there. You would need to send the information to yourself first. Let's do that first. (we send to email).
CM16: should I do that? (referring to the emailing.)
I: ok, do you see it says email sent to…
CM16: perfect.
I: ok, let's use the email you sent to yourself and hit related articles. (I explain the function to him).
CM16: how does it relate them?
I: From information in the original abstract. It is good for browsing to find more form an already good abstract.
CM16: I see, its one click away.
I: ok, will you look over the list and let me know if they are ok?
CM16: Whether they are great or not? Actually these ones are very good. They are ones that I would want to read. "exercise for treating fibromyalgia" that's very good, they are ones I would want to prescribe.
I: Do you want to send them to yourself?
CM16: Do I have to get into it to do that?
I: Nope
CM16: oh, just click it.
(He sends them)
I: Well done. Ok, go back, and we'll cover links. (I explain books to him). It is good to find a whole book on a particular subject.
CM16: hmm, that makes sense.

Linkout:
I: have you ever used this before?
CM16: No
I: Ok, this shows you where to get the article. A map to the where the article is located.
CM16: Ok.

Limits:

I: ok, you can do Boolean searches. But add more limits to the scope of the subject. (I explain all of the limits to him).
CM16: lets start with English, and humans is good. Most of them are females.
I: really why?
CM16: the disease (Fibromyalgia) is prone to women more. Actually the article we read before said it is three to one.
I: it sounds like a really uncomfortable disease.
CM16: Yes, it is a strange one. A lot of times its just, they don’t really know what’s wrong and so its just fibromyalgia. Here is the- what you were saying RCTs. Let me just try this one.
I: right and then you must put your terms in.
CM16: So the same thing as before…
I: Well, because you chose men.
CM16: yah, I’d be surprised… oh, actually it found some.
I: ok, and just to remind you it says up there what your limits are. Ok, so look through the abstracts and tell me if the limits actually worked.
CM16: Well they are all about humans but I don’t see anything specifically about men.
I: well, you might want to look at the abstract- this is just the citation.
(Takes some time to look through them)
CM16: They don’t really say who it was.
I: it might be that since they don’t mention any gender that … you know
CM16: Yes, it could have been either or.
I: Try another one.
CM16: Yeh, again they don’t really say. But I am sure there were men in the study. I would be surprised to see if it was really only men.
I: Hmm, may be if it was because it was men and humans, but I don’t really know. But it did bring up only RCTs.
CM16: yeh, same articles (We took ‘men’ off). I have never heard of men having it before. So, if unclick this (in limits) it gets rid of all of them?
I: yes, but go back in an look to see how it works.
CM16: ok, I see.

History:
I: Ok, do you see what this is?
CM16: that is the first one I punched in… and the second one and then the limits. So its our history.
I: Exactly. (I explains the interface to him … all of the numbers and such.) Ok if you hit number 18 and then hit AND and then hit, try that one (another search) and then hit GO (this is Boolean via history – we combine a search for low back pain and fibromyalgia and get 109 articles)
CM16: (looks through the results) What does the orange mean… free full text … so I don’t have to go anywhere else to get the article?
I: Exactly, very good. It in PMC. Now lets compare this one to just a free text one at another web site. Here you are in Pubmed and here you at BMJ…
CM16: Oh, ok. I see, its good… if you can find one that is free in Pubmed.

Clipboard:
I: ok, hit CB. Ok it contains no items. Now select and abstract and hit send to clipboard. (we then click into clipboard to see the abstract there – I explain why how he might use this to aggregate information.)
CM16: Before you email them to yourself you would probably email them to yourself. That’s a nice one actually.

I: you can send hundreds of citations to a clipboard and send the ones you really want to the email. Any questions?
CM16: nope, I think I am good.
I: Do you think you will remember everything?
CM16: I hope so…

I: So what are your thoughts?
CM16: I like that it is free. They (the school) talk so much about evidence based care in medicine and chiropractic and you have to have research articles in order to attempt to that. You need a way to do it and Pubmed is the only way I know of.
I: Is it easier or harder than you thought it would be?
CM16: well, I have used it a few times, but it is fairly simple. Everytime I have used it is has been Boolean. But these ones (the other tools) are easy so now that I have seen it.
I: Which is your favourite feature?
CM16: Favourite? They are all so good … but I liked the RA button for speeding things up. Umm, emailing stuff was very quick and handy, before I would've selected everything and then emailed the copy.

Medline Plus:
CM16: I have heard of Medline, but plus I don't know.
I: (I explain what MP is)
CM16: I'd imagine a lot of people would use this after they had gone to their doctor and they just want to check up and make sure, for themselves.
I: do you think you would use this?
CM16: Well definitely because Pubmed doesn't give any answers as to what is FB, it give you 5000 treatments for fibromyalgia without ever saying what it is. But let me see what happens when I click it. Yeh, this is much more useful for finding out one specific thing, it'll give you the definition.

Further use:
CM16: well now that I have done this, the next time they give us an article I will go to Pubmed and try to find it myself just to use it a few times. Thank you, this was good, as long as the article is in Pubmed… I guess.
I: or the CMCC has it. You know how you would find out right…
CM16: you would go to that little button called. What is it again .. let me go back to Pubmed. To see if Pubmed had the article? Is it the links button? Linkout… I would have to sign in through the CMCC web site. For us to use EBSCO you have to sign into the CMCC web site and then you have everything. I'll have to remember that, links, linkout.

Interview with CM17:

BACKGROUND:
I: Why would you use Pubmed?
CM17: this school is very evidence based. So a lot of the assignments are pretty much looking up research- sensitivity, specificity, tests, prognosis. You want a lot of answers to a lot of questions they give you. If you are efficient with any of these search engines, you can find the information quite quickly. I have used it in university as well, but not to a great extent. When I got here I had to use it much more. It depends on the clinician that you get, some are really, really, really, evidence based meaning they follow everything the research says- other are more in between. The guy I had was very evidence based. So,

he expected everything you said to be referenced.

I: I don't mean to sound like a therapist or anything, but how does that make you feel? Do you think that is a valid attitude or is it a bit overboard?
CM17: It's a bit along the way I feel as well. I follow that ideology a little bit. I think the best evidence is better than, saying that I am going to everything by the evidence, looking for an RCT all the time, is probably the best care you can give somebody.
I: What's an RCT?
CM17: Randomized control trial
I: So, in the past, you've turned to Pubmed specifically to look up different articles, or have you used EBSCO?
CM17: Both. I don't know why. May be Pubmed I have used more, if I am stuck on EBSCO I will go to Pubmed to see if there is anything different but usually I find them pretty synonymous in terms of…
I: What you can do with it?
CM17: Well, that's the thing. I haven't manipulated either program, meaning Mesh terms and all that. I just go in and do my searches. And I do a lot of cutting and pasting with those so it is very easy for me to do my referencing – I do very little typing. I have a word doc. Open and I cut and paste like crazy. So, that saves me crazy amounts of time in terms of putting together a bibliography.
I: Yah, you must manage your information.
CM17: I wish I had an, what's that again … a program that you can go back and forth and manages your PDF's, uhh, reference manager.
I: So why are you interested in participating in this?
CM17: Umm, (the librarian). I work with her a lot and with CE groups and stuff. I thought it would be cool. I thought it would be cool. I am not going to be in academia much longer. It would be great to know as much as I could before I leave. I plan on using this when I leave as well.
I: That's a good point. Ebsco is proprietary, so you'll need to pay for access. You get it now through the college. Pubmed, doesn't give you access to the articles necessarily. But sometimes you have free articles …
CM17: What happened to …before the linkout used to work a lot. I used to get articles ..
I: That's what linkout is for…
CM17: Well, it doesn't allow you to get free articles as easy as it used to.
I: Hmm, I'm not sure. …

The session:
I: Ok, well, so we'll go over a few functions and features available in Pubmed that will help you with your searches. You actually might already be familiar with these features and functions, but we'll see. We'll also go over Medline Plus.
CM17: I have used Medline for patient information if I am not informed on a topic I will look into it.
I: Ok so you know what the difference is between Medline Plus and Pubmed…
CM17: Yah, like Medline Plus is the little dictionary that goes to all the Mayo clinic…
I: Right- the more colloquial version of what is in Pubmed. Ok, now, let's start off with a basic search. What should we search for?
CM17: How about dizziness?
I: ok, so put in dizziness. Do you have anything more specific than just dizziness?

CM17: what causes the dizziness.
I: Ok, so first of all you have related article and Links.
CM17: I have never used related articles, but I have used links, often.
I: ok, do you know what the difference is between doing a Boolean search and using the related articles tool?
CM17: The AND/ OR features?
I: Right. It uses the language of the sentence to limit or specify your search.
CM17: No.
I: do you know what a boolean search is?
CM17: The AND/OR features (referring to the ones that come up in history). I use AND and ORs but I never really find them useful. I prefer using related articles.
I: When Anne teaches it she teaches it using the history function. So she'll ask you to perform a bunch of searches and then she'll put them together, she'll combine them- she's actually much more schooled in this than me! But I can explain it by saying whenever you are in the pubmed database, the object of the game is to try to limit and specify the search because there is so much information in here. RA might help you if you find something that is initially decent. But if you don't, then it could take down a different path. If you start with a specific search term, it might be a good way to being.
SO, we can say "dizziness and symptoms". (I then explain Boolean using language as opposed to history). So, what would 'NOT' do?
CM17: It rules out anything involved in the last term.
I: And do you know what the database searches?
CM17: the title and the content.
I: right, and the abstract.
CM17: Ok.
I: Any meta-information (and I list examples). Ok, se we have 'dizziness AND symptoms".
(Results: 4889.)
I: Does anything catch your eye?
CM17: I would look at these here. They concern me this one might be more specific as to what I want …
I: ok, so click on it.
CM17: Often times I find I get most of the information I need just from the abstract.
I: So, you don't need access to the full text?
CM17: Well, that's not true. I do (Need access). Often times you are stuck (meaning you do indeed need the article), because they tell you 'in the article we describe such and such'. And then you do everything to get it. And we have different ways to get articles..
I: So how will you use the abstract?
CM17: If I want the gist of the article. Usually they have the findings in the abstract. So if they say, 'there isn't much research on this we decided to look at this and this is what we found.' So, often times if I am rushed, I'll just have a general idea of what they are taking about, I will scroll right down to the conclusion and see what the results are.
I: Will an abstract help you decide if you are going to use the related articles tool?
CM17: yes.
I: So, let's find an article that is free – you do have access to the text by Elsevier- b/c CMCC is a member. So, let's hit related articles and look for an icon that is green (we find one).
CM17: This one looks good.

I: So green means free …
CM17: What distinguishes whether something is free or not? Who decides if this one is free and another one is not?
I: Good question- there are lots of factors: length of time they have been around, model of publishing… it depends on the journal's policy.
CM17: how will the researchers make money then?
I: Well, researchers don't really make money off of writing an article…authors don't get paid to submit their work. Ok, so, do you see how the article opened up in a different window (we continue looking through the article and the website).

Send to:
(He sends himself the free article that he liked… - knows the send to email. Don't explain it to him).
I: oh, here is another free one- but they are asking you to log in..
CM17: So they'll give it to me if I log on?
(we browse around a little, find the article. Didn't really need log on).
Send to text:
I: SO let's say you don't necessarily want to email it to yourself, you send it to text, it strips it of all of its formatting. You can save this really easily.
CM17: that's cool. I think I will use it in the future.

Links:
I: Ok, let's head to links, quickly. We'll start with books.
CM17: I have never used this.
I: (I explain book).
CM17: ok so it means it is 4 times that they have mentioned the term or something…
I: ok, scroll up for a little bit… know that you are in the books database –(I show him the bookshelf tab up top).
CM17: ok, all this stuff here, I never know what any of that is cuz I have never used it.
I: Oh, ok… those are other databases as well – medline, genome – if you are into other disciplines or fields of study you go into that database.
CM17: If I have something I am stuck on, then I probably would use this, if there isn't much articles. Especially at the hospital I am at now. Luckily its UofT, they have sharing privileges- we get pretty much any book or article. So right now, it would be useful- but as soon as I am done, no, unless the book is free online.
I: Ok, so let's go back to our original article and go to Linkout. (we go there). Do you understand what this is?
CM17: I use it, so I guess sometimes I try to get the articles that way. Or I go to Medline to get more general information on the topic
I: I explain more about linkout – (we to the libraries tool). This is a roadmap to the article. You'll see which libraries carry the article.
CM17: before, the people that supplied it, there would be more availability for free if you went to linkout. Like if you went in any way, it might not have it. But if you went to linkout it would have it. It was like a glitch in the system that everyone would use but then all of a sudden, boom, you would need a password. Like they cut down it.
I: really,
CM17: uh huh.
I: well, linkout is to provide access to it. I mean you could also get access to it from the

logo. But the point is that there is always more than one point of access. So that's the idea…
Limits:
I: Ok, do you know what limits is?
CM17: What, the human, and years and all that stuff.
I: Yes! Oh, why I am surprised?
(We type some stuff in)
I: have you ever sued this before?
CM17: I have seen it used, because we have done research projects before. So, the research people use all this sort of stuff.
I: Well, this is another way of being more specific like as with Boolean. (I explain about all the different options… English…).
CM17: the only reason why I don't do that (referring to putting on abstracts) is because we don't have availability, but it's a good way to judge between good and crappy articles. There isn't opportunity to that. Like if I had the PDF for al of the articles I would just look at them.
I: What do you mean you don't have access to all of the articles?
CM17: Well, we don't have access to all of the journals.
I: Oh, right.
CM17: or if they aren 't in English and stuff. A lot of medical research is done in German and Dutch…
I: Would you use an article that does not have an abstract?
CM17: I used one because .. well if every article had a PDF I would just go to that. I would scan it quickly and go to the parts that I like.
I: Ok, so its not important to have abstracts on…
CM17: No.
I: OK, do you know what subsets is?
CM17: nope.
I: it divides the field of research up into more topics and stuff. You might want as an example, CAM Articles and journals. (I go over publication types, RCTs,,,)
CM17: What if you want all?
I: Just put all. (We go over the rest of the options).
CM17: So, what's Mesh?
I: (I explain controlled vocabulary).
CM17: we can put chiropractor, chiropractic… like when you put a colon…
I: Actually that is a little different. We can go through that after.
(We do the search)
CM17: What I meant was, why I rely on abstracts, because it takes so long to get the article or it is not available at all. It would be great if they had standardized ratings for articles. A standard they had across the boards. It would be nice to have a rating for Pubmed.
I: Well, that's in a sense already been done because the articles have been peer reviewed.. but it's a good suggestion. Ok, so look at the list and let me know whether limits did a god job of finding things for you.
CM17: yup- there's a few.
I: Any you might want to send to yourself?
CM17: So, I'd have to open it first to send them?
I: Nope just select it, and send it to your email. And notice that your limits are still there

you would have to remove them ..

History:
I: I am sure you have used this because of the librarian…
CM17: No, not really. I am trying to figure out this one… the 'my Pubmed' – so you have your own reference manager.
(We try it out)
(we go back to history)
I: (I go over the tool, combine search tools…)(We try combining a few searches).
CM17: Ok, history is pretty useful- Iwould use that.

Clipboard:
I: (We read over clipboard rules)
CM17: oh that's great- so I don't have to copy and paste like crazy… so I can put them on this clipboard and send them all to text and … oh ok.
I: Yes, well why don't we try sending what's on the clipboard.
CM17: oh, that's good, cuz I copy and paste on a word document and its waste of time and the strategy that I use is that I have to do each of these individually.

I: So, what's your favourite feature?
CM17: I think that last one is going to save me a heck of a lot of time.
I: And in terms of searching, what's your favourite feature?
CM17: I think limits is pretty useful but I use Boolean quite a bit. I think all three. Related articles I use after I Boolean. So I use them sequentially.

<u>Interview with CM18:</u>
I: Have you ever used Pubmed before?
CM18: Yes but we didn't really have to do much of it, and I didn't really learn it, until fourth year and I was given the basics on Cochrane, Pubmed and the other databases – there are quite a few and I just found that I would use them and I wasn't that successful with them. Except for Pubmed. Pubmed just worked for me. I would get the journals from upstairs in the library, and I have almost forgotten everything else. Its just what's worked for me, what was simple, what I needed to get done for deadlines, was Pubmed. And I probably don't even use that to its full extent.

The session:
I: you know Pubmed is a more of an index then a journals database. Which means that you don't really have access to the articles. You have access to information about the articles.
CM18: Some are online though …
I: (I explain the project – trying to understand how people use public research, publishers' role in publishing paper journals, different online… I explain what we will be doing in the session).
CM18: This is great because after this year I won't have access.
I: And that's a good point... unless you keep up a membership with the CMCC. (We arrive at the Pubmed homepage. I orient him to the homepage – explain the different indexes…. Why they exist, and how to access them). Let's start by searching nutrition.

CM18: (types nutrition into the search field).
I: There are a lot of hits that we got. 19,0000. Ok, let's start first by explaining the interface – or citation - to you. The first thing is that, do you see the authors? Next to it is an icon- the icon shows whether there is an abstract or not, and whether something is Open Access or not. You'll see a green bar if its open access. You've seen that already?
CM18: yeh, I've printed some that are …
I: So then it says the name of the journal, issue, year. Then you have a select bo- its radio button which you can click or unclick. Over to the side over there you have Related Article and Links. So that's basics. You know that already, I'm sure.
CM18: Yes.
I: Now let's do a Boolean search. Do you know what a Boolean search is?
CM18: No, but I have heard the word before.
I: Ok, so the object of them game that we are playing is you want to have your search, and because there is so much info out there, you want really be very specific about what you are looking for. You don't have the time to read 20,000 articles, or even read 20,000 articles- your time is precious.
CM18: Thank you, I'm glad someone understands.
I: ok, so the way to do it is, be more specific about your topic. Start with nutrition- but what about nutrition.

Boolean:
CM18: ok, umm, nutrition and disease prevention, let's say.
I: Do you see how if you put an AND between nutrition and disease prevention you would actually be limiting the topic?
CM18: Yes
I: So if you did nutrition and disease prevention NOT umm, I can't think of anything…
CM18: Oh, ok, I get it. NOT longevity or quality of life.
I: So, do you see how that further limits it?
CM18: Yeh
I: so the more you add to your search strong the more specific it could get it- picture a funnel effect. So let's add another search string.
CM18: Ok, so you can use AND or NOT?
I: And or not
CM18: Oh, I see, ok
I: ok, let's start with AND, it is always easiest.
CM18: Upper case? Lower case?
I: Doesn't matter. Ok, let's try for nutrition and chiropractic
CM18: (types it in. Gets 37)
I: Nice. Ok, did you see what happened? So scroll through the list and have a look. Choose one you think is interesting.
CM18: Pick one that's full (open access).
I: At this point pick one that is relevant to your interests.
CM18: Here's a good one.
I: Ok, so that you would see ordinarily – because you have a membership.
CM18: Right so this means I need a membership with Pubmed? Or this particular company?
I: Pubmed is free
CM18: Ok

I: But you need a membership to Elsevier.
CM18: Right.
I: So you can always find information about articles, and most always have access to abstracts and information about the article because Pubmed does that for free and everywhere you are in the world, you can always access the information about the information. And actually, a good question is how important an abstract is to you and would you need access to the full article, or does this provide you enough information?
CM18: Right, your asking me? Oh, umm often the full article is useful for me, because whether it applies to my patient or not, the abstract does not tell me enough. The abstract sort doesn't often have that detail. But it sorta depends. Sometimes it's enough. Like treatment stuff- I need the specifics- like how you would actually do something. But if its advice or recommendations- this would be enough. Are you asking in a particular case?
I: Well, sure, whatever, and even the article that I sent you –
CM18: Usually the full article. The abstract- I can't break down the methods they used and if there's any flaws in it. I won't get it form the abstract. So I would like to see the whole thing.
I: And the article that I sent you- did you understand it, read it?
CM18: Yeh, it was good. It was comparing different kinds of foods and vitamins on their own. Spinach was better then other phyto-chemicals that were available in foods.
I: oh- so I should eat my spinach?
CM18: Yes! Definitely eat your spinach.
I: Ok, so you now you understand our object as kinds of drilling down, and you know what I mean by drilling down as a metaphor. So, what we are going to do next is where are going to use another tool that facilitates drilling down but without that Boolean aspect to it. In other words, we won't be combining search terms.
CM18: Oh, ok,.. Boolean is the combining of search terms.
I: right, that's what we call it. But scroll up for a second, and we'll use another feature, and its called related articles. So if you click on related articles
CM18: (He clicks).
I: Ok, so here is your first article that we looked at, and basically all of the articles that follow were found based on that initial article.
CM18: Yeh, I do that sometimes, and I find they pick something and I find that what they think is similar is not what I think is similar.
I: That's a good point. Which is why the Boolean might be a little more effective in terms of finding exactly what you are looking for.
CM18: Right.
I: Here you have all of the information in the citation and it finds article that relate tot hat in this automatic way. So you have used Related Articles in the past?
CM18: Yes. Often its quite helpful but I'll be surprised with what they got. But I'll use it quite often. I haven't used links.
I: Ok, so scroll down and let me know whether this was an effective tool?
CM18: (looks), some yes some … no, definitely. Some just go on to obstructive lung disease and diet- so about half? May be…

Links: Linkout and Books
I: Ok, let's take the first one, and we'll go to links and then to link out. Links are comprised of two thing: books and Linkout. Let's go to Linkout first. Linkout provides a sort of road map to where the actual article is located. Here is where you will get access

to the articles.
CM18: And these are sites you would have to pay for?
I: I think so, but not all of them. It depends… (there is a link to Ontario Scholar's portal – we try for it but get nothing). So, Linkout provides information as to where the article is. So if you wanted to check to see if your library had it, you could go to libraries, and scroll down and choose, umm..
CM18: Would we be on there? Private ones?
I: I'm not sure. Only because
CM18: Well, the naturopath (Naturopath institute) is here… Well we have our own list… well, if we can get on, we have a library page that opens up, we can check as students what's available here… not through Pubmed.
I: But this list is a little different because it tells you about other resources linked up to this citation.
CM18: Ok, I got it
I: Ok, go back to links, and go to books. So first of all, do you see how everything is underlined here?
CM18: Yes, (he looks). Ok, so this is what I would click on and then it would give me what book I want?
I: So here is what your search topic is, so you don't forget (nutritional supplements) and here you see what database you are in- books. You are no longer in Pubmed.
CM18: Right.
I And these indicate all of the books that contains your phrase, nutritional supplements. And here is also how many times it is mentioned in the book.
CM18: Oh really, that's neat.
I: But it doesn't mean to say that the book will be incredibly useful. It comes up 39 times… but if you find that you are looking for fibromyalgia for instance, and want to know more about it, beyond the scope of a journal article, then this is a good way to find a book on it.
CM18: There's too many items or words, there's no other words – I don't know what my theory is on that. Does it say where the book is?
I: yes, well sometimes you can find them online. Click into HSTAT, the first one.
CM18: These are books that come up online? These aren't free the books…
I: Sometimes they are online and sometimes they are not. Click on full contents…
Ok, so the name of the book is "Health Services Technology Assessment text" and if you scroll down a little bit these are the chapter names.
CM18: oh, ok.
I: And if you click into one of the chapters…its more information on that- it summarizes
CM18: So it's not really a [whole] chapter on it ….

History:
I: Ok, so can you go back? I want you to click on history. So this gives you your history. SO you see your first search. Number one: nutritional supplements. Keep your eye on number one: it says time – 10:59, Then it says results- we got a ton of results, and then our second search was nutrition and chiropractic- that was when we played around with Boolean- it gave you 37 results. Then we chose related articles, and it found and article related to number two. So where did number 3 go?
CM18: Oh yeah, here?

I: Well number three is contained in number 4.
CM18: oh, ok, because it was Boolean?
I: This is where the history of what you do is recorded…. Ok, now just do a regular search under chiropractic.
(He does the search)
I: Now choose number one (in history), and then hit AND now choose number 6, and then hit go. You just searched number one combined with number six. Number one was nutrition and number 6 was chiropractic. And that was basically a Boolean search.
CM18: So I should get the same number: 37.
I: As the first one- it's a bit of an easier way to wrap your mind around Boolean. You could then add another search and combine all of them
CM18: Right- just keep adding them.
I: so let's say you were sitting at your computer for a few hours. And you were say to yourself, I want to go back to my first search and combine it with my last one: you go to history because it is an unlimited record of all of your searches.
CM18: Is it limited by time?
I: I don't think so.
CM18: may be when you just turn it off.
I: well, if you click out of Pubmed then you lose it.
CM18: that makes sense.
I: Every time you log on it is a session, so it keeps the session for as long as you want it.

Send to email:
Now go back to number 7, number one and number six. And let's say you really like this article. Select it.
CM18: ok
I: So when I say select it I mean click into that button by the side. And now we are going to practice sending it to yourself in various ways. The easiest way is to send it through your email. So I want you to guess how to do that.
CM18: Ok (looks over the screen and thinks). Would I have to have my email on? Oh no it would just send it automatically.
I: Well, you would put your email address in there.
CM18: And that would actually work huh? Obviously. For some reason I thought … so that's sent.
I: Umm, go back- no back, and now you have to hit mail..
CM18: So you don't need any type of email version already set up.
I: well you see it says ' email sent to …"
CM18: Oh, so its done.
I: there you go. So make sure to see that pink ribbon up there.
CM18: Can't wait to get that, its quite a surprise…
I: Right, so you'll learn more about menopause (the article he sent was about menopause).

Send to:
I: Ok, so let's say you are at your computer at home. And you want this article, But you don't want to copy and paste, you want it simple and easily. You want to try send to text. So select it first- you never want to forget to select it.
CM18: What is text again?

I: Well, you'll find out in a second. You see how this looks kind of like type writer?
CM18: Yeh, I've never seen this.
I: Send to text strips it of all formatting so what you saw before had all of the formatting in it. It had links and stuff- if you copy and past a page from your browser into a word document you get all of this formatting and it messes it up.
CM18: Oh, ok.
I: the point is that this strips the formatting out of it and gives you straight text. So you can copy and paste this into a word document and save this in a folder on your desk-top. It gets more useful- once you do more searches you realize how to use this. It helps to eliminate some of the steps involved in emailing if you are at your computer or you want to print out.
CM18: Oh, ok, so it is just like, you would save it like this? What would you do with this information?
I: It is very good for printing. It really helps to organize all of the information. You could save it in a file under the search headings…
I:ok, so go back now and select four articles..
CM18: Just any four?
I: yes.
CM18: Ok, and now send it to the clipboard. (Finds clipboard function. Reads the instructions about clipboard).
I: So you can keep your stuff on the clipboard as long as you don't leave it for more than 8 hours – you can take a 4 hours nap.
CM18: nearly a full night's sleep.
I: But let's say you have done 300 searches and for each search you have found one article that is useful. Each article that is useful you select and send it to the clipboard. Now you have 300 articles on the clipboard- what do you do with it?
CM18: Clipboard to text?
I: Exactly, and you see how it says clipboard over there?
CM18: Yes..
I: (We open up the clipboard)
CM18: I would select them and send these to text?
I: you are really catching on. Or you can send it to your email.
(He does it)
CM18: OH that's useful.
I: Do you see how this might be important?
CM18: Yes. It would be very efficient in terms of search strategy because the next step is to have access to them- how to find them (referring to the citations).
I: Right.
CM18: But if I could get them for free, wouldn't it be more useful to print them off right way? Like when you do it, you do this and then you go to the library to photocopy.
I: Or you go (the reference librarian), and say, I want to print these off!
CM18: Right but I wouldn't be able to do that all the time (Displays intention to use the research after schooling).
I: So, if you find your articles online all the time, even so you may want to go back to them, or if you are writing a paper you might want to cite them, or if you want to send them to a client… that's why you would do it?
CM18: Can you find article and turn them into to text? Turn them into word (i.e cut and paste them into word).

I: you can .. but that's another lesson- more about computers and less about Pubmed.

Limits:
I: basically, what limits does is provide another way of limiting your research topic in a non-Boolean way. So what you are doing is adding more parameters- but not the subject matter. So you are not saying chiropractic AND nutrition NOT longevity NOT xyz. What you are saying is the information about it I want to limit. I want info from core clinical journals, about women, people between 18 and 26…
CM18: Right.
I: So that's what all these limits are and that's why they are a little different than the Boolean.
CM18: oh I see, right, I often use Human, gender not necessarily. I haven't really looked at the other ones. This is just subsets. Ok… sometimes review sometimes date. Ok, so you can just do authors…
I: And another good thing is: only items with abstracts…
CM18: Why would I want that? Don't they all have abstracts? No, actually some don't – I thought abstracts was the minimum you needed to have something show up, but some of them have just titles. So abstracts would mean that you would get items that, like the PDFs have abstracts, but they at least have the abstracts? (Here I am concerned that he does not get the difference between a citation, found in an index, and the full text…).
I: It has at least an abstract
CM18: oh, ok, so I might as well click on that.
I: Usually, there are some things that every journal article will have. All of them will have an author
CM18: right
I: every journal article will have a language… because obviously its written in some language.
CM18: Right.
I: every journal article will have a title, a publication date… but not every journal article will have an abstract.
CM18: Right. So by clicking on this I will cancel out full journals
I: No, you will only cancel out article that don't have abstracts.
CM18: that don't have abstracts ... ok, so this is a good one to click on.
I: And I think English is incredibly useful … I don't want articles in German
CM18: Yes, that would make sense- there not translated are they?
I: Sometimes the abstracts are but…also, its could be useful to limit to humans- so we don't get articles about let's say rats or something
CM18: yes that would be useful…
I: So now with these limits on, now go ... so we are still doing search 1 and 6. So do you see how it says in the yellow band there, English and humans?
CM18: Yes.
I: Ok, so go to 'History' do you see how it says that your last search was searching number 1 and number 6 with Limits English and humans, so just to see how that was recorded.
CM18: Yeh, got it…
I: Ok, now do you see how in your limits checkbox there is still a check in it?
CM18: Yes
I: Well that shows that your limits are still on

CM18: Yeh.
I: So you don't have to keep on putting them back on. You can choose your limits and then just keep on doing more searches.
CM18: Right, and just click it on or off. Back and forth. I see,
I: Useful?
CM18: Yes. Definitely especially to summarize stuff like meta-analysis or reviews or something, and any other stuff too. I don't use details..
I: click on it and see what it is. It explains to you exactly what the search was comprised of.
CM18: Oh, ok, that makes sense.
I: If you didn't really understand what happened you would look at details to understand more about your own search. This hour went by quickly.
CM18: Well, I have sort of done it all before… is there anything else you wanted to go through?
I: Ok, so that's Pubmed, any questions?
CM18: No, so I can access this, Pubmed is free? IS that where a lot of health professionals get their information.
I: Actually, yes many doctors and researchers use this…
AC: Sometimes there is not enough articles

Medline Plus:
AC: I have never heard of it. I would use it as a new grad to convey information that is in layperson's terms to communicate with clients and stuff.
